# *N‐*Heterocyclic Silylenes as Ligands in Transition Metal Carbonyl Chemistry: Nature of Their Bonding and Supposed Innocence

**DOI:** 10.1002/chem.202001062

**Published:** 2020-07-27

**Authors:** Mirjam J. Krahfuß, Jörn Nitsch, F. Matthias Bickelhaupt, Todd B. Marder, Udo Radius

**Affiliations:** ^1^ Institut für Anorganische Chemie Julius-Maximilians-Universität Würzburg Am Hubland 97074 Würzburg Germany; ^2^ Department of Theoretical Chemistry Amsterdam Center for, Multiscale Modeling (ACMM) Vrije Universiteit Amsterdam De Boelelaan 1083 1081 HV Amsterdam The Netherlands; ^3^ Institute for Molecules and Materials (IMM) Radboud University Heyendaalseweg 135 6525 AJ Nijmegen The Netherlands; ^4^ Institute for Sustainable Chemistry & Catalysis with Boron Julius-Maximilians-Universität Würzburg Am Hubland 97074 Würzburg Germany

**Keywords:** *N-*heterocyclic carbenes, silylenes, silylene complexes, stereoelectronic parameters, transition metal complexes

## Abstract

A study on the reactivity of the *N‐*heterocyclic silylene Dipp_2_NHSi (1,3‐bis(diisopropylphenyl)‐1,3‐diaza‐2‐silacyclopent‐4‐en‐2‐yliden) with the transition metal complexes [Ni(CO)_4_], [M(CO)_6_] (M=Cr, Mo, W), [Mn(CO)_5_(Br)] and [(*η* ^5^‐C_5_H_5_)Fe(CO)_2_(I)] is reported. We demonstrate that *N‐*heterocyclic silylenes, the higher homologues of the now ubiquitous NHC ligands, show a remarkably different behavior in coordination chemistry compared to NHC ligands. Calculations on the electronic features of these ligands revealed significant differences in the frontier orbital region which lead to some peculiarities of the coordination chemistry of silylenes, as demonstrated by the synthesis of the dinuclear, NHSi‐bridged complex [{Ni(CO)_2_(*μ*‐Dipp_2_NHSi)}_2_] (**2**), complexes [M(CO)_5_(Dipp_2_NHSi)] (M=Cr **3**, Mo **4**, W **5**), [Mn(CO)_3_(Dipp_2_NHSi)_2_(Br)] (**9**) and [(*η* ^5^‐C_5_H_5_)Fe(CO)_2_(Dipp_2_NHSi‐I)] (**10**). DFT calculations on several model systems [Ni(L)], [Ni(CO)_3_(L)], and [W(CO)_5_(L)] (L=NHC, NHSi) reveal that carbenes are typically the much better donor ligands with a larger intrinsic strength of the metal–ligand bond. The decrease going from the carbene to the silylene ligand is mainly caused by favorable electrostatic contributions for the NHC ligand to the total bond strength, whereas the orbital interactions were often found to be higher for the silylene complexes. Furthermore, we have demonstrated that the contribution of *σ*‐ and *π*‐interaction depends significantly on the system under investigation. The *σ*‐interaction is often much weaker for the NHSi ligand compared to NHC but, interestingly, the *π*‐interaction prevails for many NHSi complexes. For the carbonyl complexes, the NHSi ligand is the better *σ*‐donor ligand, and contributions of *π*‐symmetry play only a minor role for the NHC and NHSi co‐ligands.

## Graphical abstract

**A study on the reactivity** of the *N‐*heterocyclic silylene (NHSi) Dipp_2_NHSi (1,3‐bis(diisopropylphenyl)‐1,3‐diaza‐2‐silacyclopent‐4‐en‐2‐yliden) with different transition metal carbonyl complexes and calculations on the electronic features of these NHSi ligands are presented and compared to NHC (*N‐*Heterocyclic Carbene) ligands. The reaction of Dipp_2_NHSi with the [Ni(CO)_4_], [M(CO)_6_] (M=Cr, Mo, W), [Mn(CO)_5_(Br)] and [Fe(*η* ^5^‐C_5_H_5_)(CO)_2_(I)] leads to the mono‐ and bis‐silylene complexes [{Ni(CO)_2_(*μ*‐Dipp_2_NHSi)}_2_] (**2**), [M(CO)_5_(Dipp_2_NHSi)] (M=Cr **3**, Mo **4**, W **5**), [Mn(CO)_3_(NHSi)_2_(Br)] (**9**) and [(*η* ^5^‐C_5_H_5_)Fe(CO)_2_(Dipp_2_NHSi‐I)] (**10**).

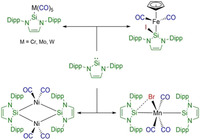

## Introduction

**Scheme 1:**
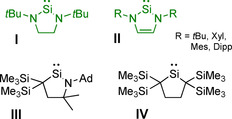
Examples for *N‐*heterocyclic silylenes and related molecules.6b, 6c, 6d, 6e, 6f, 6g

The isolation of the first *N‐*heterocyclic carbene (NHC), 1,3‐diadamantyl‐imidazolin‐2‐ylidene, by Arduengo in 1991^1^ led to the opening of a wide field of research utilizing the new class of ligands, which was further substantially expanded by Bertrand *et al*. in 2005 with the synthesis of cyclic (alkyl)(amino)carbenes (cAACs).^2^ The efficiency of NHCs^3^ and cAACs^4^ as excellent ancillary ligands for transition metal complexes and for stabilization of low‐coordinate transition metal centers is the result of their strong *σ*‐donor properties and their sterically demanding structures.^5^ The silicon analogues of NHCs, *N‐*heterocyclic silylenes (NHSi)^6^ and related compounds,^7^ however, have attracted less interest over the last few decades compared to their carbon counterparts. Due to the divalent silicon atom of NHSis, these silylenes are Lewis acids and bases simultaneously (*vide infra*), which opens up a multitude of different reaction pathways. With the “Arduengo‐type” *N‐*heterocyclic silylenes **I** and **II**, cyclic alkyl(amino)silylene **III** and dialkylsilylene **IV** there is a huge variety of compounds known containing an active silicon(II) center in variable electronic and steric environments (Scheme 1).^8^

Herein, we focus on “Arduengo‐type” *N‐*heterocyclic silylenes and their similarities and differences compared to the NHCs widely employed in transition metal chemistry. For the saturated and unsaturated *tert*‐butyl substituted silylenes *t*Bu_2_NHSi^H2^ (**I**) and *t*Bu_2_NHSi (**II**), which are the most studied *N‐*heterocyclic silylenes in coordination and organometallic chemistry thus far, several transition metal complexes are known. However, their application seems to be rather limited, as from their first syntheses in 1994 (**II**) and 1996 (**I**), respectively, only a small number of transition metal complexes have been reported (see below). Moreover, complexes of *N*‐aryl substituted *N‐*heterocyclic silylenes Mes_2_NHSi and Dipp_2_NHSi are even more scarce. Compared with the numerous complexes and applications of NHCs in transition metal chemistry, organometallic chemistry and catalysis using NHSi compounds as ligands is not as well developed and, at the outset of our work, we wondered whether there is a specific reason for this.

**Scheme 2:**
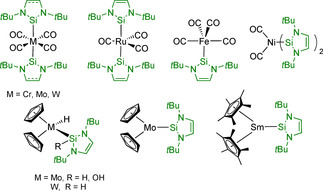
Metal(0) carbonyl and bent‐metallocene complexes of the *N‐*heterocyclic silylenes **I** and **II**.

For the *tert*‐butyl substituted NHSis *t*Bu_2_NHSi^H2^ (**I**) and *t*Bu_2_NHSi (**II**), several heteroleptic transition metal carbonyl complexes have been reported, namely [M(L)_2_(CO)_4_] (M=Cr, Mo, W; L=**I**, *t*Bu_2_NHSi), [Fe(*t*Bu_2_NHSi)(CO)_4_], [Ru(*t*Bu_2_NHSi)_2_(CO)_3_] and [Ni(CO)_2_(*t*Bu_2_NHSi)_2_] (Scheme 2).^9^ Silylene ligated group 6 bent‐metallocene complexes [(*η* ^5^‐C_5_H_5_)_2_M(H)(*t*Bu_2_NHSi)] (M=Mo, W) and [(*η* ^5^‐C_5_H_5_)_2_Mo(*t*Bu_2_NHSi)]^10^ have been prepared, which were obtained by irradiation or prolonged heating of a mixture of *t*Bu_2_NHSi and the metallocene dihydrides [(*η* ^5^‐C_5_H_5_)_2_M(H)_2_], or from the reaction of the silylene with phosphine‐stabilized [(*η* ^5^‐C_5_H_5_)_2_Mo(PEt_3_)].^10^ Another representative of NHSi‐stabilized bent‐metallocene type complexes, and the only silylene lanthanide compound known to date, is [(*η* ^5^‐C_5_Me_5_)_2_Sm(*t*Bu_2_NHSi)], which is seemingly not especially stable, as the silylene ligand is easily substituted by THF giving [(*η* ^5^‐C_5_Me_5_)_2_Sm(THF)_2_].^11^

**Scheme 3:**
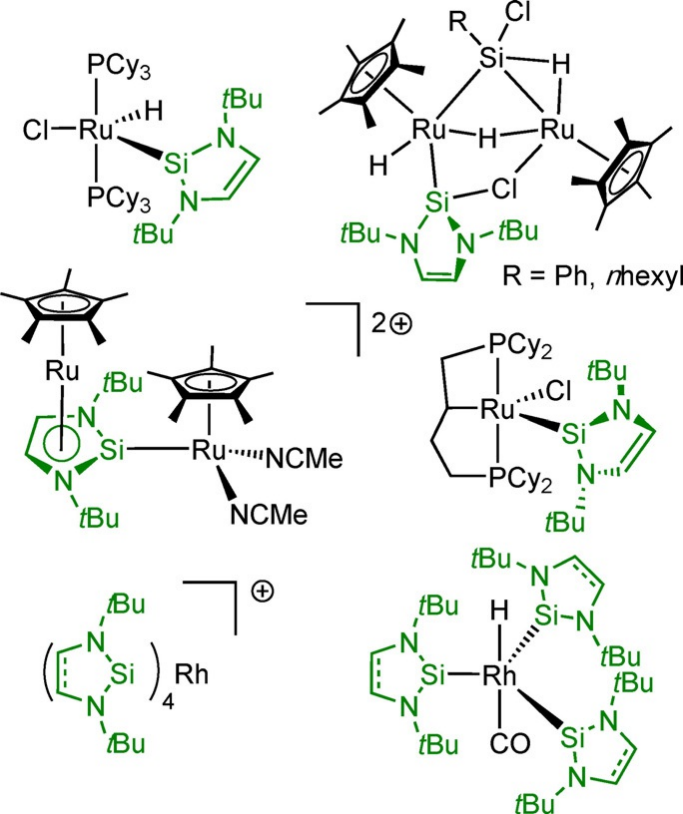
Neutral and ionic ruthenium and rhodium complexes of the *N‐*heterocyclic silylenes **I** and **II**.

With the d^8^ and d^9^ metals Ru and Rh, a variety of compounds has been prepared (Scheme 3). Hill and co‐workers reported the coordinatively unsaturated compound [Ru(PCy_3_)_2_(H)(Cl)(*t*Bu_2_NHSi)], prepared by replacement of the *η* ^2^‐bound dihydrogen ligand in [Ru(PCy_3_)_2_(*η* ^2^‐H_2_)(H)(Cl)] with the silylene.^12^ Interestingly, this reaction did not occur if the mesityl carbene Mes_2_Im was used, as, in this case, a phosphine ligand is replaced to form [Ru(PCy_3_)(Mes_2_Im)(*η* ^2^‐H_2_)(H)(Cl)].^12^ The complex [(*η* ^5^‐C_5_Me_5_)Ru(*t*Bu_2_NHSi)(Cl)] was obtained from the reaction of *t*Bu_2_NHSi with tetranuclear [(*η* ^5^‐C_5_Me_5_)Ru(*μ*‐Cl)]_4_. This mononuclear Ru complex was subsequently converted into dinuclear [{(*η* ^5^‐C_5_Me_5_)_2_Ru}_2_(H)(*μ*‐H)(*μ*,*η* ^2^‐HSiRCl)(*μ*‐Cl)(*μ*,*η* ^2^‐*t*Bu_2_NHSi)] (R=Ph, *n*‐hexyl, Scheme 3) upon reaction with primary silanes.^13^ Furthermore, the ionic complex [(*η* ^5^‐C_5_Me_5_)Ru(NCMe)_3_][OTf] cleanly reacts with *t*Bu_2_NHSi to afford [(*η* ^5^‐C_5_Me_5_)Ru(NCMe)_2_(*t*Bu_2_NHSi)][OTf] and the solvation of this complex in THF afforded [(*η* ^5^‐C_5_Me_5_)Ru(*η* ^5^:*η* ^1^‐*t*Bu_2_NHSi)Ru(*η* ^5^‐C_5_Me_5_)(NCMe)_2_][OTf]_2_ featuring an interesting *η* ^5^:*η* ^1^‐silylene ligand.^13^ Another NHSi representative in Ru chemistry is the coordinatively unsaturated compound [Ru(*η* ^3^‐dcypb)(Cl)(*t*Bu_2_NHSi^H2^)] (dcypb=bis(dicyclohexyl)‐1,4‐phosphinobutane) which reacts promptly with small molecules such as H_2_O, H_2_ and CO.^14^ The Rh complex [Rh(PPh_3_)_3_(H)(CO)] reacts with three equivalents of *t*Bu_2_NHSi to give [Rh(H)(CO)(*t*Bu_2_NHSi)_3_].^15^ The cationic Rh^I^ compounds [Rh(L)_4_][BAr^F^] (L=*t*Bu_2_NHSi^H2^ **I**, *t*Bu_2_NHSi **II**) were obtained by treatment of [Rh(cod)_2_][BAr^F^] (BAr^F^=tetrakis(3,5‐bis(trifluoromethyl)phenyl)borate; cod=1,5‐cyclooctadiene) with four equivalents of **I** or **II** in hexane.^16^

**Scheme 4:**
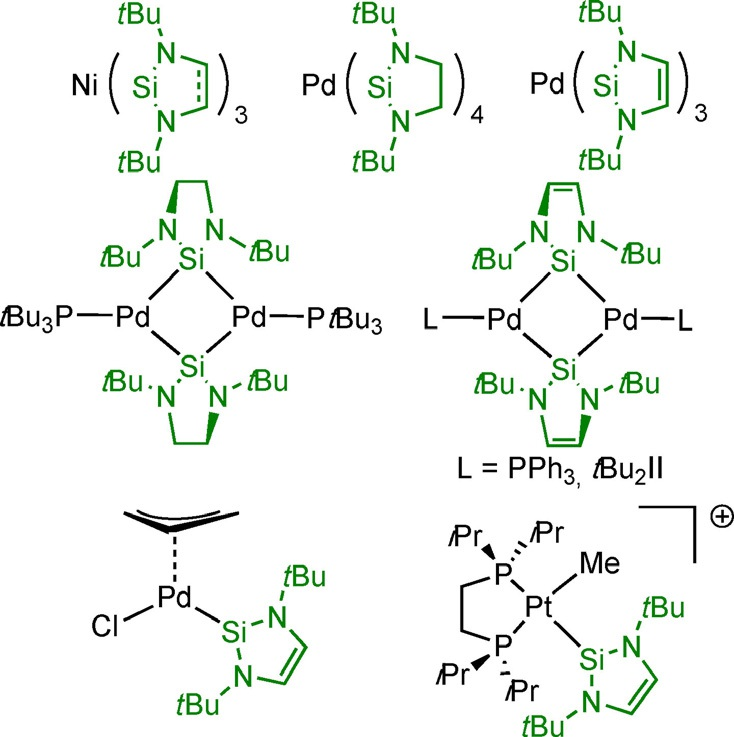
Neutral and ionic group 10 complexes of the *N*‐heterocyclic silylens **I** and **II**.

For the d^10^ metals Ni, Pd and Pt, a variety of NHSi complexes are known (Scheme 4). Substitution of both cod ligands of [Ni(cod)_2_] by *t*Bu_2_NHSi^H2^/ *t*Bu_2_NHSi (=L) in THF results in the formation of the homoleptic, trigonal planar complex [Ni(L)_3_].^17^ The allyl Pd complex [Pd(*η* ^3^‐C_3_H_5_)(*t*Bu_2_NHSi)(Cl)],^18^ and the phosphine‐stabilized silylene‐bridged dimeric Pd^0^ compound [Pd(PPh_3_)(*t*Bu_2_NHSi)]_2_ 19 are two examples of Pd NHSi complexes. The reaction of [Pd(P*t*Bu_3_)_2_] with *t*Bu_2_NHSi^H2^ affords the homoleptic four‐coordinate Pd^0^ complex [Pd(*t*Bu_2_NHSi^H2^)_4_], which forms a dinuclear silylene‐bridged Pd^0^ complex with one phosphine at each palladium center upon addition of P*t*Bu_3_. Loss of two *t*Bu_2_NHSi ligands led to the formation of a dinuclear silylene‐bridged Pd^0^ compound, which was further stabilized by one silylene ligand at each Pd center. Reaction of [Pd(cod)(CH_3_)_2_] with six equivalents of *t*Bu_2_NHSi^H2^ or four equivalents of *t*Bu_2_NHSi also afforded [Pd(*t*Bu_2_NHSi^H2^)_4_] and [Pd(*t*Bu_2_NHSi)_3_], while two NHSi equivalents were consumed during the reduction of the Pd^II^ precursor giving a methylated disilane from *t*Bu_2_NHSi^H2^ or *t*Bu_2_NHSi(CH_3_)_2_, respectively.^20^ Element–hydrogen bond activation at a cationic platinum compound and subsequent addition of the silylene gives the complex [Pt(dippe)Me(*t*Bu_2_NHSi)][B(C_6_F_5_)_4_] (dippe=1,2‐bis(di‐isopropylphosphino)ethane).^21^

**Scheme 5:**
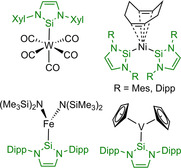
Transition metal complexes bearing *N*‐aryl substituted *N*‐heterocyclic silylenes Xyl_2_NHSi, Mes_2_NHSi and Dipp_2_NHSi.

Most of the coordination chemistry of *N‐*heterocyclic silylenes has been investigated using the *tert*‐butyl substituted derivatives *t*Bu_2_NHSi^H2^ and *t*Bu_2_NHSi, as summarized above. For other silylenes, for example *N‐*aryl substituted systems, only a few transition metal complexes are known (see Scheme 5). The only transition metal complex with Xyl_2_NHSi is [W(CO)_5_(Xyl_2_NHSi)] which was synthesized *via* irradiation of [W(CO)_6_] in THF and subsequent addition of the silylene.6f The number of transition metal complexes bearing Mes_2_NHSi is limited to the heteroleptic Ni^0^ complex [Ni(cod)(Mes_2_NHSi)_2_], for which the Dipp_2_NHSi analogue [Ni(cod)(Dipp_2_NHSi)_2_] is also known. Substitution of the cod ligand by a third silylene, as observed for *t*Bu_2_NHSi^H2^ and *t*Bu_2_NHSi, was not successful.6e For the Dipp‐substituted silylene, more examples exist, for example, a three‐coordinate iron(II) silylene complex [Fe(N(SiMe_3_)_2_)_2_(Dipp_2_NHSi)] reported by Layfield *et al*.^22^ Another interesting example is the complex [(*η* ^5^‐C_5_H_5_)_2_V(Dipp_2_NHSi)], which was obtained by reaction of the silylene with vanadocene, as it was not possible to obtain the analogous product from the corresponding carbene Dipp_2_Im.^23^

Given our long term interest in the development of the transition metal chemistry of alkyl‐24 and aryl‐25 substituted NHCs as well as cAACs,^26^ we became interested in the coordination properties of *N*‐aryl‐substituted *N*‐heterocyclic silylenes. Herein we present results using the Dipp‐substituted NHSi Dipp_2_NHSi as a ligand and reactant in transition metal carbonyl chemistry as well as some stereo‐electronic parameters for Dipp_2_NHSi.

## Results and Discussion

**Figure 1:**
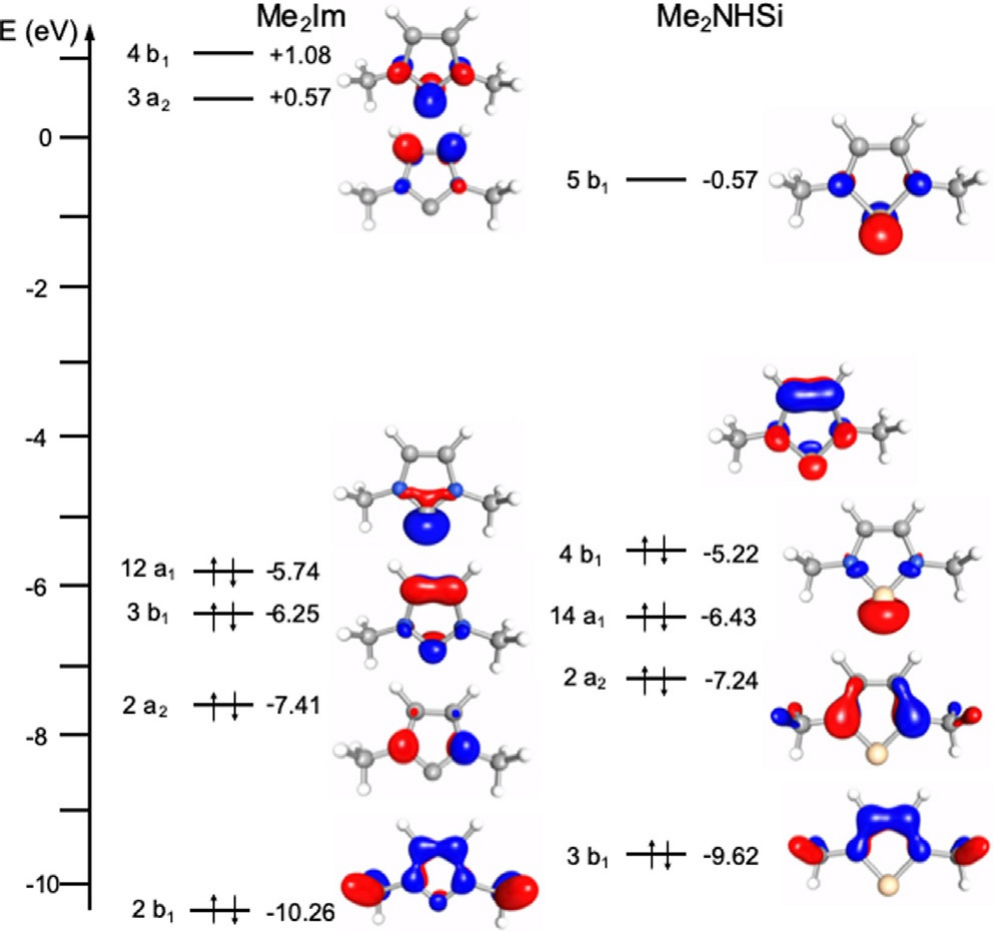
Main electronic features of 1,3‐dimethylimidazolin‐2‐ylidene Me_2_Im (left side) and the corresponding silylene equivalent Me_2_NHSi (right side). Energies were calculated at the DFT/def2‐TZVPP/B3LYP level of theory, and orbital plots are drawn at the 0.1 isosurface.

First, we compare the frontier orbitals suitable for coordination of the NHSi ligand with those of the NHC‐type ligands. DFT calculations (def2‐TZVPP/B3LYP) were performed on the *N‐*methyl substituted model Me_2_NHSi (1,3‐dimethyl‐1,3‐diaza‐2‐silacyclopent‐4‐ene‐2‐yliden) and its NHC analogue. The molecular orbitals of these molecules and their energies are shown in Figure 1. For our purpose it is instructive to recall the main electronic features of the NHC 1,3‐dimethylimidazolin‐2‐ylidene (Figure 1, left).^5^ A quantitative MO analysis reveals that the frontier orbitals of 1,3‐dimethylimidazolin‐2‐ylidene may be considered as those of a 6 *π*‐electron aromatic system, superimposed on the carbene *σ*‐type orbital 12a_1_ at −5.74 eV, which is the HOMO of the molecule. The orbitals 2b_1_, 2a_2_, 3b_1_, 3a_2_ and 4b_1_, similar to those of the well‐known cyclopentadienyl anion, are the occupied orbitals of the *π*‐system and have no nodal plane (orbital 2b_1_ in *C*_2*v*_ symmetry, at −10.26 eV) or one nodal plane (2a_2_, −7.41 eV and 3b_1_, −6.25 eV), whereas the unoccupied *π*‐orbitals (3a_2_, +0.57 eV and 4b_1_, +1.08 eV) have two nodal planes. These pairs of orbitals are not degenerate due to the heteroatomic substitution of the aromatic ring and thus *C*_2*v*_ symmetry. The 4b_1_ (LUMO+1, +1.08 eV) orbital is mainly centered at the carbene carbon atom and is mostly composed of the carbene p_x_‐orbital (62 %), while for the 3b_1_ orbital the p_x_ contribution is lower (22 %, based on gross Mulliken contributions of AOs to the MOs). The HOMO of Me_2_Im is the 12a_1_ orbital at −5.74 eV, usually referred to as the carbene *σ*‐orbital, which contains carbene carbon p_z_ (49 %) and s (33 %) character. Within our level of theory, we calculate an energy gap of 6.82 eV between 12a_1_ and 4b_1_.

The frontier orbitals of Me_2_NHSi differ considerably from those of the corresponding NHC. First, the order of the orbitals changes, as the HOMO of Me_2_NHSi is not the silylene *σ*‐orbital, but the 4b_1_ orbital, which should be weakly *π*‐donating upon coordination to a transition metal. However, as the silicon p_x_ contribution is low (24 %) for this orbital, the overlap of 4b_1_ with a metal centered d_*π*_‐type orbital should be rather small. The silylene *σ*‐orbital 14a_1_ at −6.43 eV lies at much lower energy compared to the carbene *σ*‐orbital 12a_1_ at −5.74 eV of Me_2_Im and also has much more s character (48 % s and 32 % p_z_ for Me_2_NHSi vs. 33 % s and 49 % p_z_ for Me_2_Im) compared to the NHC. According to the compositions and the orbital energies, one would expect that the NHSi is a much weaker *σ‐*donor ligand compared to an NHC. On the other hand, the *π*‐accepting orbital 5b_1_ lies much lower in energy than 4b_1_ of the NHC and has a much larger p_x_ contribution (76 % for NHSi vs. 62 % for NHC), which would be in line with much better *π*‐accepting properties of the NHSi ligand. These typical features, a *π*‐donor HOMO such as 4b_1_, a reverse orbital order of 4b_1_ and the silylene *σ*‐orbital 14a_1_, which lies energetically much lower compared to the carbene *σ*‐type orbital, and an energetically low lying *π*‐acceptor orbital can also be found for the 2,6‐diisopropyl‐phenyl substituted Dipp_2_NHSi. Figure S1 in the Supporting Information shows the important frontier orbitals of the NHSis Dipp_2_NHSi and Me_2_NHSi with respect to those of commonly used NHC ligands.

**Scheme 6:**
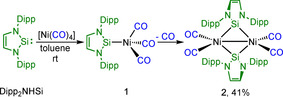
Synthesis of the [{Ni(CO)_2_(*μ*‐Dipp_2_NHSi)}_2_] **2**.

The Tolman Electronic Parameter (TEP) is a widely used method to determine the electronic characteristics of a ligand. This parameter is based on measurement of the C−O stretching vibration of a_1_ symmetry in complexes of the type [Ni(CO)_3_(L)].^27^ This stretching frequency allows one to draw conclusions regarding electron density at the metal center of the nickel carbonyl complex and, therefore, of the donor properties of the ligand.26e In order to synthesize a complex of the type [Ni(CO)_3_(NHSi)], we reacted [Ni(CO)_4_] with one equivalent of Dipp_2_NHSi in toluene at room temperature. This reaction led to a colorless solution which turned purple upon removal of the solvent, and a deep‐purple solid was isolated in moderate yield (**2**, 41 %; Scheme 6).

**Figure 2:**
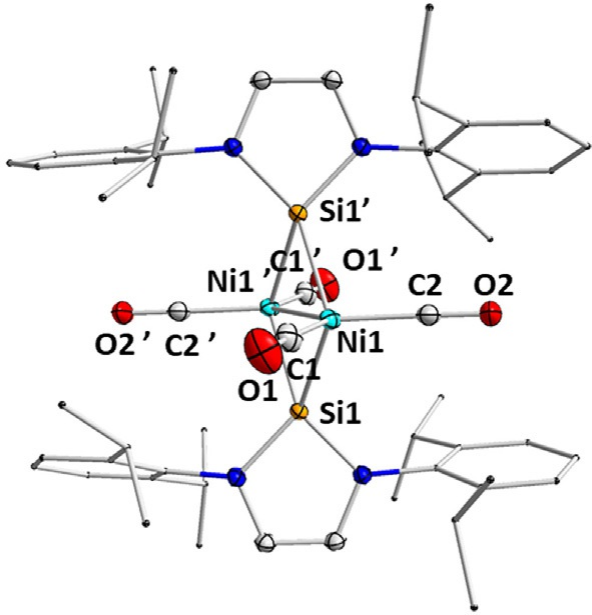
Molecular structure of [{Ni(CO)_2_(*μ*‐Dipp_2_NHSi)}_2_] **2** in the solid state (ellipsoids drawn at 50 % probability; hydrogen atoms omitted for clarity). Selected bond lengths [Å] and angles [°]: Ni1−Ni1’ 2.5218(5), Ni1−Si1 2.2798(5), Ni1−Si1’ 2.3090(5), Ni1’−Si1’ 2.2798(5), Ni1’−Si1 2.3090(5), Ni1−C1 1.7760(19), Ni1−C2 1.8079(18), C1−O1 1.146(2), C2−O2 1.132(2), Ni1‐Si1‐Ni1’ 66.672(16), N1‐Si1‐N2 88.22(6), C1‐Ni1‐C2 115.55(8), C1‐Ni1‐Ni1’ 127.72(6), C2‐Ni1‐Ni1’ 116.64(6), Si1‐Ni1‐Si1’ 113.327(16), Si1‐Ni1‐C1 113.18(6), Si1‐Ni1‐C2 102.61(5).

Compound **2** was characterized by ^1^H, ^13^C and ^29^Si NMR spectroscopy in solution and *via* IR spectroscopy and single‐crystal X‐ray diffraction in the solid state. In the ^1^H NMR spectrum, the expected signals of the silylene ligand were shifted slightly downfield compared to the free NHSi. The ^13^C{^1^H} NMR spectra also displayed a shift of the resonances for the NHSi ligand as well as one resonance for the carbonyl carbon atoms at 195.7 ppm. In the ^29^Si NMR spectrum, a resonance at 121.9 ppm also indicated the formation of a silylene transition metal complex. Two carbonyl stretching bands at 1971 and 2010 cm^−1^ were observed in the IR spectrum at rather low energies compared to other complexes of the type [Ni(CO)_3_(L)].24a, 24b, 24m, 27b, 29 The X‐ray crystal structure (Figure 2) of the product **2** revealed that the reaction of [Ni(CO)_4_] with one equivalent of Dipp_2_NHSi leads to a dimeric, silylene‐bridged complex [{Ni(CO)_2_(*μ*‐Dipp_2_NHSi)}_2_] **2**, which was, most probably, formed from the colorless intermediate [Ni(CO)_3_(Dipp_2_NHSi)] **1** (Scheme 6) upon CO elimination and subsequent dimerization. The dimer is built from two [Ni(CO)_2_(Dipp_2_NHSi)] moieties, which are connected by a nickel–nickel bond (Ni1–Ni1’ 2.5218(5) Å) of a similar length to those in bridged dinuclear nickel complexes (2.36–2.54 Å).24m, 28 The molecules lie on an inversion center located between the Ni atoms. Both the silicon and the nickel atoms are tetrahedrally coordinated. The silylene ligands are each bonded to the nickel atoms *via* one longer (2.3090(5) Å) and one shorter (2.2798(5) Å) Ni−Si bond, and the nickel atoms are thus slightly unsymmetrically bridged by the silylene ligands. The nickel‐carbon distances are unexceptional.

The corresponding NHC complexes [Ni(CO)_3_(NHC)], which are ligated with Mes_2_Im or Dipp_2_Im, are reluctant to replace a carbonyl ligand to give three‐coordinate complexes.27b, 29 Bis‐carbene complexes of the type [Ni(CO)_2_(NHC)_2_] are accessible either by carbonylation of bis‐NHC complex precursors, by reaction of [Ni(CO)_4_] with sterically less demanding NHCs, or by addition of a carbene to coordinatively unsaturated complexes bearing a bulky NHC ligand, such as [Ni(CO)_2_(*t*Bu_2_Im)].24a, 24b, 24m, 27b, 29 Cyclic (alkyl)(amino)carbenes react with [Ni(CO)_4_] to give the 18 VE complexes [Ni(CO)_3_(cAAC)] and complexes [Ni(CO)(cAAC)_2_] which are available by further substitution of [Ni(CO)_3_(cAAC)] with additional cAAC or from the reaction of suitable NHC precursors such as [Ni(CO)_2_(*t*Bu_2_Im)] with two equivalents of the cAAC.26a, 26b Carbene‐bridged, dinuclear nickel complexes have rarely been observed, and the only example of a NHC‐bridged nickel complex was prepared by Lappert *et al*. in 1977.29a To shed more light on the energetics of this reaction, DFT (TURBOMOLE/def2‐SV(P)/BP86) calculations were performed on the dimerization of [Ni(CO)_2_(Dipp_2_Im)] and [Ni(CO)_2_(Dipp_2_NHSi)] (Figure S2). The optimized geometry of [Ni(CO)_2_(*μ*‐Dipp_2_NHSi)]_2_ is similar to the experimentally observed structure (distances Ni−Ni: 2.5348 Å, Ni−Si: 2.2946–2.2968 Å; Ni−C: 1.7724–1.7861 Å; Figure S2), and the calculated CO stretching vibrations (1975 and 2001 cm^−1^) agree well with the experimentally observed frequencies (1971 and 2010 cm^−1^). Whereas the dimerization of [Ni(CO)_2_(Dipp_2_Im)] is highly repulsive on the energy hypersurface, for which we calculate a Δ*G(298)* of +275.03 kJ mol^−1^, the dimerization of the NHSi complex [Ni(CO)_2_(Dipp_2_NHSi)] *via* the bridging of the NHSi ligand is favorable by Δ*G(298)*=−80.27 kJ mol^−1^. We consider the strong interaction of the *π*‐accepting orbitals of the NHSi ligand with occupied d‐orbitals of the planar [(CO)_2_Ni–Ni(CO)_2_] dimer as one of the main driving forces for the dimerization process.

Furthermore, we were interested in comparing the bonding in Ni–NHSi *vs*. Ni–NHC and with that in classical phosphine complexes. To focus on the interaction of one d^10^ nickel atom with the NHSi and NHC ligands, respectively, DFT calculations were performed at the TZ2P/BLYP/ZORA/D3(BJ) level of theory on the mono‐ligated model complexes [Ni(Me_2_Im)] and [Ni(Me_2_NHSi)]. The results of the energy decomposition analysis of the Ni−C and Ni−Si bond in [Ni(Me_2_Im)] and [Ni(Me_2_NHSi)] are given in Table 1 and are compared to the Ni−P bond of [Ni(PPh_3_)]. The metal‐carbene, ‐phosphine and ‐silylene bond distances were fixed at the equilibrium distances of 1.753 Å (Ni−C), 2.020 Å (Ni−P) and 2.002 Å (Ni−Si), respectively.

**Table 1:** Energy Decomposition Analysis (kJ mol^−1^) of the Ni−C and Ni−Si bonds (C_2V_ symmetry) and of the Ni−P bond (*C*_3*v*_ symmetry) in [Ni(L)] complexes. Metal–carbene, ‐phosphine and ‐silylene bond lengths are 1.753 Å (Ni−C), 2.020 Å (Ni−P) and 2.002 Å (Ni−Si), respectively.

l‐Ni	Δ*E_int_*	Δ*E_Pauli_*	Δ*V_elstat_*	Δ*E_disp_*	Δ*E_oi_*	Δ*E_oi_* ^*σ*^	Δ*E_oi_* ^*πy*^	Δ*E_oi_* ^*πx*^	Δ*E_oi_* ^δ^
Me_2_Im	−479.5	+890.7	−824.6	−24.5	−521.0	−336.2	−146.6	−38.6	+0.4
Ph_3_P	−431.3	+737.1	−704.0	−41.0	−423.5	−218.8	−204.6		−0.0
Me_2_NHSi	−426.5	+665.0	−679.1	−17.7	−394.8	−165.9	−128.3	−99.8	−0.9

In *C*_2*v*_ symmetry Δ*E*_oi_^**πy**^ corresponds to b_2_ and Δ*E*_oi_^**πx**^ to b_1._ In *C*_3*v*_ symmetry Δ*E*_oi_^**π**^ corresponds to the e representation.

Details of the EDA analysis of the Ni−C and Ni−Si bonds (in C_2V_ symmetry) and of the Ni−P bond (in *C*_3*v*_ symmetry) in the mono‐coordinated complexes [Ni(L)] are provided in Table 1. These results reveal that the electronic properties of the silylene ligand are probably more similar to those of the phosphine ligand than to those of the NHC ligand. The interaction energy Δ*E_int_* between the neutral ligands L and the nickel atom decreases in the order Me_2_Im (−479.5 kJ mol^−1^) > PPh_3_ (−431.3 kJ mol^−1^)≈Me_2_NHSi (−426.5 kJ mol^−1^), and a decrease in the orbital interaction Δ*E_oi_* of −521.0 kJ mol^−1^ (Me_2_Im)≫−423.5 kJ mol^−1^ (PPh_3_)>−394.8 kJ mol^−1^ (Me_2_NHSi) was calculated, in which the orbital interactions between the PPh_3_ and the Me_2_NHSi are close in energy. The NHC ligand is certainly the strongest *σ*‐donor ligand among these three ligands, with a *σ*‐orbital interaction of −336.2 kJ mol^−1^ (64.5 % of Δ*E_oi_*) and a *π*‐orbital interaction energy of −185.2 kJ mol^−1^ (35.5 % of Δ*E_oi_*). For the NHSi ligand, −165.9 kJ mol^−1^ (42.0 %) arises from *σ*‐donor contribution and −228.1 kJ mol^−1^ (57.8 %) from the *π*‐interaction. Although we cannot differentiate here between *π*‐donation and *π*‐acceptance, it is interesting to note that: (i) *σ*‐interaction is much weaker for the NHSi ligand compared to NHC; and (ii) *π*‐interaction prevails for the NHSi ligand. This is in line with the general orbital picture of the ligands (Figure 1) in which both the *σ*‐donor and the *π*‐acceptor orbital of the NHSi ligand lie at much lower energies compared to the NHC ligand, whereas a *π*‐donating orbital of the NHSi ligand, the HOMO, lies at higher energy compared to the carbene ligand. The calculations on the phosphine ligated complex [Ni(L)] provide a rather balanced picture concerning *σ*‐ and *π*‐contributions to the orbital interaction, i.e. −218.8 kJ mol^−1^ (51.7 %) for the *σ*‐interaction and −204.6 kJ mol^−1^ (48.3 %) for the *π*‐interaction. According to the calculated Voronoi deformation density charges (Table 2), in all cases net charge is transferred to the nickel atom in the order NHC (−0.103 e^−^)>NHSi (−0.081 e^−^)>PPh_3_ (−0.068 e^−^). Thus, there is a slightly larger charge transfer to the nickel for the silylene compared to the phosphine ligand, due to a larger *π*‐donation or weaker *π*‐acceptor interaction (or both), which compensates the stronger *π*‐interaction found for PPh_3_.

**Table 2:** Voronoi *deformation* density (VDD) charges (as fraction of one electron) of Ni in the complexes [Ni(L)] and [Ni(CO)_3_(L)] complexes and corrected TEP values of [Ni(CO)_3_(L)] (in cm^−1^, available experimental values in curly brackets). Positive VDD charge (VDDC) values signify depletion of electrons. Metal–carbene, ‐phosphine and ‐silylene bond lengths are 1.997 Å (Ni−C), 2.251 Å (Ni−P) and 2.219 Å (Ni−Si), respectively.

L	VDDC (L‐Ni)	VDDC (L‐Ni(CO)_3_)	TEP (L‐Ni(CO)_3_)
Me_2_Im	−0.103	+0.166	2053 {2051}^30^
Ph_3_P	−0.068	+0.121	2066 {2069}27a
Me_2_NHSi	−0.081	+0.100	2076

A similar analysis was performed for [Ni(L)(CO)_3_] using Me_2_Im, Me_2_NHSi and PPh_3_ as the ligand (Table 3). Compared to [Ni(L)], three good *σ*‐donating and excellent *π*‐accepting carbonyl ligands have been added to the complex. As a consequence, the whole M–L interaction should be weaker, and much of the *π*‐contributions should be located at the M−C bond to the carbonyl ligands, that is, contributions of the NHC, NHSi and PPh_3_ ligand should be much less developed. This stabilization can be traced to the relative energies of the acceptor and donor orbitals of the transition metal component. The *σ*‐bonding a_1_ acceptor orbital of [Ni(CO)_3_] is 0.84 eV lower in energy compared to that of [Ni]. This trend is even more pronounced for the occupied d‐orbitals, for which stabilization by the three carbonyl ligands is essential. These [Ni(CO)_3_] donor orbitals, responsible for *π*‐back donation to the ligand, are stabilized by almost 6.17 eV compared to the d‐orbital level of the Ni atom in its d^10^s^0^ electron configuration.

**Table 3:** Energy Decomposition Analysis (kJ mol^−1^) of the Ni−C and Ni−Si bonds (C_s_ symmetry) and the Ni−P bond (*C*_3*v*_) in [Ni(CO)_3_(L)] complexes. Metal–carbene, ‐phosphine and ‐silylene bond lengths are 1.997 Å (Ni−C), 2.249 Å (Ni−P) and 2.219 Å (Ni−Si), respectively.

L‐Ni	Δ*E_int_*	Δ*E_Pauli_*	Δ*V_elstat_*	Δ*E_disp_*	Δ*E_oi_*	Δ*E_oi_* ^*σ*^	Δ*E_oi_* ^*π*^	Δ*E_oi_* ^*σ*^
Me_2_Im	−202.4	+570.5	−513.2	−44.3	−215.4	−194.0	−21.4	–
Ph_3_P	−161.2	379.8	−306.9	−64.8	−169.3	−112.4	−56.4	−0.4
Me_2_NHSi	−170.5	+524.6	−438.6	−34.2	−222.3	−189.6	−32.6	–

For [Ni(L)(CO)_3_], this difference is reflected in the lower interaction energy Δ*E_int_* of −202.4 kJ mol^−1^ (Me_2_Im)>−170.5 kJ mol^−1^ (Me_2_NHSi)>161.2 kJ mol^−1^ (PPh_3_) between L and [Ni(CO)_3_]. Interestingly, the largest orbital interaction Δ*E_oi_* was calculated for the silylene ligand, i.e. −222.3 kJ mol^−1^ for Me_2_NHSi, compared to −215.4 kJ mol^−1^ for Me_2_Im and −169.3 kJ mol^−1^ for PPh_3_. The electrostatic contributions are thus largest for the NHC complex. As the orbital interaction in [Ni(Me_2_Im)(CO)_3_] (−215.4 kJ mol^−1^) is even weaker than that in [Ni(Me_2_NHSi)(CO)_3_] (−222.3 kJ mol^−1^), we attribute the decrease in Δ*E_int_* to the decrease in the electrostatic term Δ*V_elstat_* from −513.2 kJ mol^−1^ for [Ni(Me_2_Im)(CO)_3_] to −438.6 kJ mol^−1^ for [Ni(Me_2_NHSi)(CO)_3_].

The more stabilizing Δ*E_oi_* for [Ni(Me_2_NHSi)(CO)_3_] can be mainly attributed to superior *π*‐bonding, as the interaction with Me_2_Im reveals a larger *σ*‐contribution Δ*E_oi_* ^*σ*^ for Me_2_Im (−194.0 kJ mol^−1^; 90.0 % of Δ*E*_oi_), than for Me_2_NHSi (−189.6 kJ mol^−1^; 85.3 % of Δ*E*_oi_) and PPh_3_ (−112.4 kJ mol^−1^; 66.4 % of Δ*E*_oi_) whereas *π*‐contributions Δ*E_oi_* ^*π*^ are larger for Me_2_NHSi (−32.6 kJ mol^−1^; 14.7 % of Δ*E*_oi_) compared to Me_2_Im (−21.4 kJ mol^−1^; 10.0 % of Δ*E_oi_*; cf. −56.4 kJ mol^−1^ 33.3 % of Δ*E_oi_* for PPh_3_). Although the formation of the dimer [{Ni(CO)_2_(*μ*‐Dipp_2_NHSi)}_2_] **2** prevents the experimental determination of Tolman's electronic parameter (TEP), these values were calculated for [Ni(CO)_3_(L)] (Table 2), and clearly show that Me_2_NHSi is the weakest donating ligand in this series: Me_2_Im: TEP=2053 cm^−1^; PPh_3_: TEP=2066 cm^−1^, Me_2_NHSi: TEP=2076 cm^−1^. These TEP values correlate with the Voronoi deformation density charges of Ni in the complexes [Ni(CO)_3_(L)] (Me_2_Im: +0.166; PPh_3_: +0.121, Me_2_NHSi: +0.100). We conclude that, considering the orbital interaction in [Ni(L)(CO)_3_], Me_2_NHSi has good *σ*‐donor properties similar to those of Me_2_Im, but that (i) beneficial electrostatic contributions to the Me_2_Im‐Ni interaction as well as (ii) *π*‐accepting contributions of the NHSi ligand reduce the electron density on the central metal.

The nature of the chemical bond between a transition metal and a carbene fragment CR_2_ drew the attention of theoreticians soon after the first stable transition metal carbene complex [Cr(CO)_5_{C(OMe)(Me)}] was reported in 1964 by Fischer and Maasböl.^31^ Carbene complexes became particularly interesting for theoretical analyses when experimental studies suggested that there are two categories of transition metal carbene complexes which show very different properties, namely “Fischer type” complexes,^32^ which are characterized by a *π*‐donor group X at the carbene ligand bound to a transition metal in a low oxidation state, and “Schrock type” carbene complexes,^33^ which have nucleophilic carbene ligands typically with hydrogen, alkyl, or aryl groups, but no *π*‐donor substituents at the carbene carbon atom. For historical reasons, calculations on “Fischer type” carbene complexes have been carried out on group 6 carbonyl complexes [M(CO)_5_(CR_2_)], especially those of tungsten.^34^ Subsequently, the bonding of many other neutral 2‐electron donor ligands was theoretically investigated with respect to the [W(CO)_5_] complex fragment in complexes of the type [W(CO)_5_(L)].^35^ NHCs and related molecules are “Fischer type” ligands, and calculations on [W(CO)_5_(H_2_Im)] in comparison with [W(CO)_5_(H_2_NHSi)] were reported in a theoretical study by Frenking *et al*.34m Their results are similar to our results on nickel carbonyl as outlined above. The bond dissociation energies of the NHC and NHSi ligand, calculated at the BP86/def2‐TZVPP//BP86/def2‐SVP level of theory, are, as expected, larger for the NHC complex (227.6 kJ mol^−1^ for [W(CO)_5_(H_2_Im)]) than for [W(CO)_5_(H_2_NHSi)] (185.4 kJ mol^−1^). The calculated values for the charge transfer to [W(CO)_5_] increase from the carbene complex [W(CO)_5_(H_2_Im)] (−0.47 e^−^) to the silylene complex [W(CO)_5_(H_2_NHSi)] (−0.74 e^−^), and the W–E bond order increases from [W(CO)_5_(H_2_Im)] (0.75) to [W(CO)_5_(H_2_NHSi)] (0.90). Frenking *et al*. concluded that neither the charge distributions nor the bond orders correlate with the BDEs of the NHE ligands. The decrease in the BDEs from the carbene to the silylene is determined by the intrinsic strength of the metal‐ligand bonds, Δ*E_int_*, which is in the order [W(CO)_5_(H_2_Im)] (−243.9 kJ mol^−1^)>[W(CO)_5_(H_2_NHSi)] (−201.3 kJ mol^−1^), and the authors attributed this decrease mainly to a decrease of the Pauli repulsion for the heavier homologue. A closer inspection of the trend of the electrostatic term Δ*V_elstat_* and the orbital (covalent) term Δ*E_oi_* shows that the weaker bonds are mainly caused by the former term. Interestingly, Frenking *et al*. also found that the orbital interaction in [W(CO)_5_(H_2_NHSi)] (−256.9 kJ mol^−1^) is even larger in magnitude than in [W(CO)_5_(H_2_Im)] (−223.0 kJ mol^−1^), whereas the electrostatic term Δ*V_elstat_* increases from [W(CO)_5_(H_2_Im)] (−538.1 kJ mol^−1^) to [W(CO)_5_(H_2_NHSi)] (−438.1 kJ mol^−1^). Thus, the authors concluded that the decrease of the bond strength going from [W(CO)_5_(H_2_Im)] to [W(CO)_5_(H_2_NHSi)] correlates with the decrease in Δ*V_elstat_*.

To compare with the results obtained for the nickel complexes and to corroborate the results obtained for the methyl substituted NHC or NHSi ligand, we performed calculations on the tungsten carbonyl complexes [W(CO)_5_(Me_2_Im)] and [W(CO)_5_(Me_2_NHSi)] at the TZ2P/BLYP/ZORA/D3(BJ) level of theory. It is important to note that the metal‐ligand bonding situation should change compared to the nickel carbonyl complexes as the a_1_ acceptor orbital for *σ*‐bonding in [W(CO)_5_] is stabilized by 0.72 eV compared to the acceptor orbital in [Ni(CO)_3_] and the metal d donor orbitals of [W(CO)_5_] suitable for *π*‐back donation are 0.12 eV lower in energy compared to those of [Ni(CO)_3_], which means that [W(CO)_5_] is, per se, a much poorer *π*‐electron donor for an additional ligand L in [W(CO)_5_(L)].

The results summarized in Table 4 confirm the interesting picture of the bonding of the NHSi ligand compared to the NHC ligand. Thus, (i) the intrinsic strength of the metal–ligand bonds Δ*E_int_* decrease from the carbene to the silylene ligand from −273.8 kJ mol^−1^ for [W(CO)_5_(Me_2_Im)] to −222.9 kJ mol^−1^ for [W(CO)_5_(Me_2_NHSi)]. (ii) This decrease in bond strength is caused by electrostatic contributions for the Me_2_Im complex. Whereas the contributions from Pauli repulsion remains almost constant for both complexes (538.1 kJ mol^−1^ for the Me_2_Im complex *vs*. 528.7 kJ mol^−1^ for the Me_2_NHSi complex), we compute a significant difference of the electrostatic term Δ*V_elstat_* to the bonding, i.e. −530.4 kJ mol^−1^ for [W(CO)_5_(Me_2_Im)] and −446.3 kJ mol^−1^ for [W(CO)_5_(Me_2_NHSi)]. (iii) The W−Si orbital interaction in the silylene complex [W(CO)_5_(Me_2_NHSi)] (−253.3 kJ mol^−1^) is larger than the W−C orbital interaction in the carbene complex [W(CO)_5_(Me_2_Im)] (−216.8 kJ mol^−1^), for the methylated ligands by 36.5 kJ mol^−1^ in favor of the silylene complex. (iv) The NHSi ligand is the better *σ*‐donor ligand, which is counterintuitive to the conclusions one might draw from the simple orbital picture provided in Figure 1 in combination with Fukui's frontier orbital concept.^36^ We calculate a *σ*‐contribution to the net orbital interaction of −181.8 kJ mol^−1^ for the silylene complex [W(CO)_5_(Me_2_NHSi)] and −155.5 kJ mol^−1^ for the carbene complex [W(CO)_5_(Me_2_Im)]. (v) Contributions of *π*‐symmetry play only a minor role for the NHC or NHSi co‐ligands in the presence of many good *π*‐accepting carbonyl ligands. However, as also calculated for the nickel carbonyl complexes, the *π*‐interaction between the Me_2_NHSi ligand and the tungsten atom is stronger compared to the Me_2_Im ligand (−57.7 kJ mol^−1^ for [W(CO)_5_(Me_2_Im)] and −70.3 kJ mol^−1^ for [W(CO)_5_(Me_2_NHSi)]).

**Table 4:** Energy Decomposition Analysis (kJ mol^−1^) of the tungsten‐carbene and tungsten‐silylene bond in [W(CO)_5_(Me_2_Im)] and [W(CO)_5_(Me_2_NHSi)] complexes (C_2V_ symmetry). Metal‐carbene/silylene bond distances are 2.282 Å (Ni−C) and 2.503 Å (Ni−Si), respectively.

L‐W	Δ*E_int_*	Δ*E_Pauli_*	Δ*V_elstat_*	Δ*E_disp_*	Δ*E_oi_*	Δ*E_oi_* ^*σ*^	Δ*E_oi_* ^*πy*^	Δ*E_oi_* ^*πx*^	Δ*E_oi_* ^*σ*^
Me_2_Im	−273.8	538.1	−530.4	−64.7	−216.8	−155.5	−37.3	−20.4	−3.6
Me_2_NHSi	−222.9	528.7	−446.3	−51.9	−253.3	−181.8	−37.7	−32.6	−1.2

**Scheme 7:**
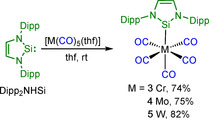
Synthesis of [M(CO)_5_(Dipp_2_NHSi)] (M=Cr **3**, Mo **4**, W **5**).

To provide some experimental data to support these calculations, and, as the dinuclear species **2** is not suitable to determine the TEP parameter, we investigated the behavior of Dipp_2_NHSi towards group 6 carbonyls. Complexes [M(CO)_5_(Dipp_2_NHSi)] **3**–**5** were synthesized by reacting [M(CO)_5_(THF)] (M=Cr, Mo, W) with Dipp_2_NHSi in THF and isolated as red (M=Cr, **3**, 74 %, M=Mo, **4**, 75 %) and orange (M=W, **5**, 82 %) solids, respectively (Scheme 7). Complex **5** decomposes very slowly in solution and in the solid state, whereas the corresponding chromium and molybdenum compounds decompose quickly in solution within 12 hours at temperatures of −30 °C, and within 7 days in the solid state.

For the carbonyl carbon atoms, a distinct upfield shift is observed in the ^13^C{^1^H} NMR spectrum going from chromium to tungsten (**3**: 215.5 ppm, 211.6 ppm, **4**: 204.1 ppm, 201.1 ppm, **5**: 196.4 ppm, 193.3 ppm). The ^29^Si NMR spectra of **3**–**5** reveal sharp singlets with the resonances clearly shifting to higher fields from chromium to tungsten (**3**: 138.2 ppm, **4**: 125.4.0 ppm, **5**: 111.2 ppm, ^1^ *J*(^183^W–^29^Si)=168.3 Hz).

**Scheme 8:**
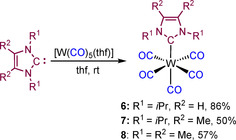
Synthesis of [W(CO)_5_(NHC)] (NHC=*i*Pr_2_Im **6**, *i*Pr_2_Im^Me^ **7** and Me_2_Im^Me^ **8**).

For comparison with common *N‐*heterocyclic carbene complexes, the compounds [W(CO)_5_(*i*Pr_2_Im)] (**6**), [W(CO)_5_(*i*Pr_2_Im^Me^)] (**7**)^37^ and [W(CO)_5_(Me_2_Im^Me^)] (**8**)^37^ were prepared from [W(CO)_5_(THF)] in good yields (**6**: 86 %, **7**: 50 %, **8**: 57 %, Scheme 8). Complexes **7** and **8** are known, but the ^1^ *J*(^183^W–^13^C) coupling constants and the X‐ray crystal structures of these compounds have not been reported previously.

**Figure 3:**
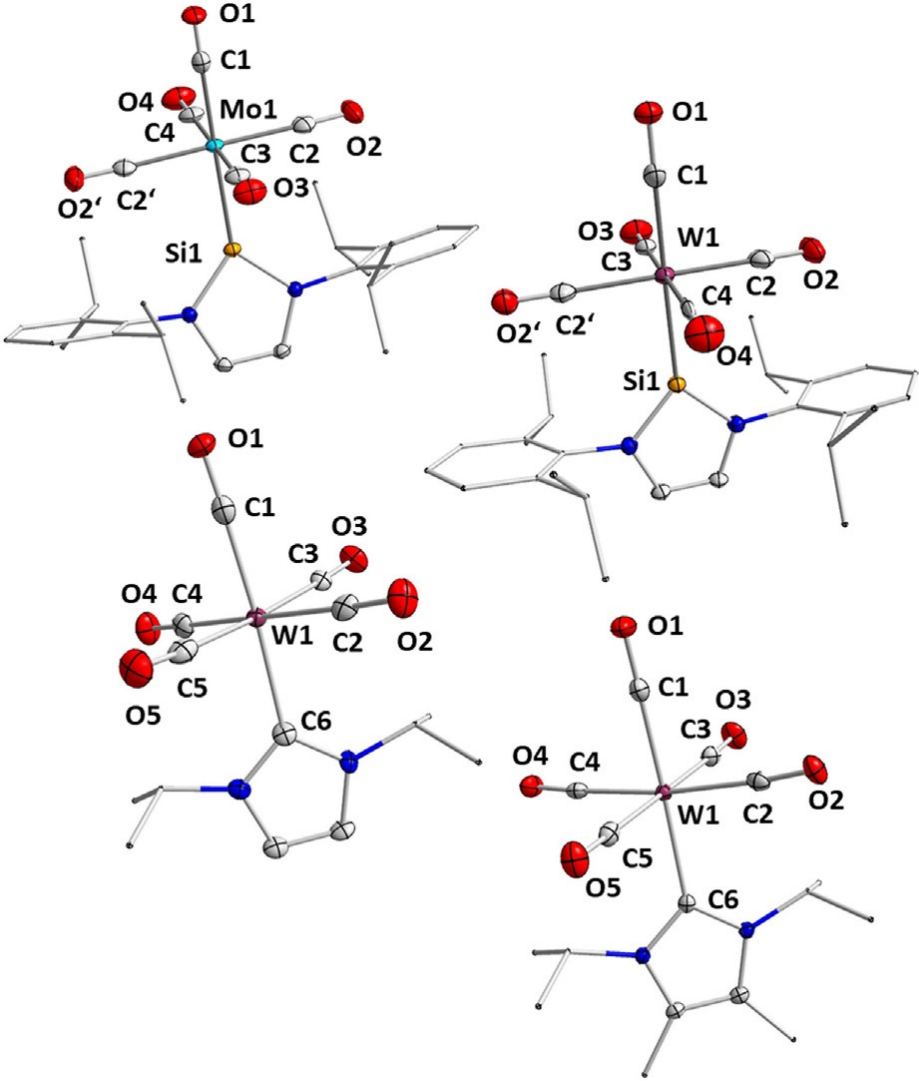
Molecular structures of **4** (top left), **5** (top right), **6** (bottom left) and **7** (bottom right) in the solid state (ellipsoids drawn at 50 % probability; hydrogen atoms omitted for clarity). Selected bond lengths [Å]: **4**: Mo1−Si1 2.4594(8), Mo1−C1 2.017(3), Mo1−C2 2.050(2), Mo1−C3 2.045(3), Mo1−C4 2.053(4); **5**: W1−Si1 2.4576(14), W1−C1 2.010(5), W1−C2 2.051(4), W1−C3 2.038(5), W1−C4 2.021(6); **6**: W1−C6 2.272(4), W1−C1 1.999(4), W1−C2 2.033(4), W1−C3 2.034(4), W1−C4 2.049(4), W1−C5 2.047(4); **7**: W1−C6 2.2930(18), W1−C1 1.9883(19), W1−C2 2.0367(19), W1−C3 2.033(2), W1−C4 2.0414(19), W1−C5 2.047(2).

Complexes **6**–**8** have been fully characterized (see Experimental and Supporting Information), including the X‐ray crystal structures of **6** and **7**. The molecular structures of these complexes as well as of [Mo(CO)_5_(Dipp_2_NHSi)] **4** and [W(CO)_5_(Dipp_2_NHSi)] **5**, with selected bond lengths and angles, are shown in Figure 3, and a detailed analysis of the W−C and C−O bond lengths of the tungsten complexes [W(CO)_5_(L)] (L=Dipp_2_NHSi **5**, *i*Pr_2_Im **6**, *i*Pr_2_Im^Me^ **7**) is provided in Table S1. Single crystals of **4** and **5** were grown from saturated hexane solutions at −30 °C, and those of **6** and **7** were obtained from saturated solutions of the respective complexes in toluene/hexane mixtures at −30 °C. All four complexes adopt nearly perfect octahedral structures with four carbonyl ligands arranged in the plane between the silylene and the *trans*‐CO ligand. In case of the carbene complexes, a staggered conformation of the four in‐plane carbonyl ligands and the NHC ligand is observed, while the silylene complexes adopt an eclipsed arrangement with two of the carbonyls pointing directly to the aryl substituents of the NHSi ligand. The silylene complexes **4** and **5** show similar M−Si (**4**: 2.4594(8) Å, **5**: 2.4576(14) Å) and M−C_trans_ (**4**: 2.017(3) Å, **5**: 2.012(6) Å) bond lengths, in agreement with distances found for [W(CO)_5_(Xyl_2_NHSi)] (Xyl=1,3‐bis(2,6‐dimethylphenyl)‐1,3‐diaza‐2‐silacyclopent‐4‐en−2‐ylidene) reported by Müller *et al*. (W−Si 2.4568(13) Å, W−C(*trans*) 2.058(5) Å, W−C(*cis*) 2.056 Å).6f For the NHC complexes **6** and **7**, the bond lengths between the tungsten atom and the carbene carbon atom are W1−C6 2.272(4) Å (**6**) and W1−C6 2.2930(18) Å (**7**) and, similar to those of the *n*Pr_2_Im complex [W(CO)_5_(*n*Pr_2_Im)] (W−C_NHC_ 2.265(4) Å).^38^

The W−C_trans_ distances of the NHC complexes **6** and **7** are shorter (e.g. **6**: W1−C1 1.999(4); **7**: W1−C1 1.9883(19)) Å) compared to the W−C_cis_ distances (**6**: W−C_cis_ 2.033(4)–2.049(4) Å; 7: W−C_cis_ 2.033(2)–2.047(2) Å), as is also the case for the molybdenum silylene complex **4** (Mo1−C1 2.017(3); Mo1−C_cis_ 2.045(3)–2.053(4) Å), but is only found for two of the four *cis*‐situated CO ligands of the tungsten silylene complex **5** (W1−C3 2.038(5), W1−C4 2.021(6) Å). The bond lengths of the *cis*‐carbonyl ligands of the silylene complex **5**, which are arranged directly above the aryl rings of the silylene ligand, are W1−C2/C2* 2.053(4) Å and are thus significantly elongated compared to the other two *cis*‐carbonyl ligands.

Utilizing the crystal structures of **4, 6** and **7** we calculated the percent buried volume (%*V*_bur_) which is a useful tool to analyze the steric hindrance of a ligand in the coordination sphere of a metal center.^39^ A comparison of the calculated values for the complexes [Ni(CO)_3_(L)] and [W(CO)_5_(L)] is shown in Table 5. As [Ni(CO)_3_(L)] could not be isolated, we used a model compound instead. The values obtained differ quite significantly for the two central atoms nickel and tungsten which is caused by the different geometries of the [Ni(CO)_3_(L)] and [W(CO)_5_(L)] complexes and the distance between the central atom and the coordinating atom of the ligand used for the assessment of the percent buried volume. We used the values approximated from the bond lengths determined by the X‐ray crystal structure analyses of the complexes, which is 2.0 Å for the nickel compounds and 2.5 Å for the tungsten complexes.

**Table 5:** Percent buried volume (*V*_bur_%) of the complexes [Ni(CO)_3_(L)] (L=Dipp_2_NHSi, Dipp_2_Im, Mes_2_Im, *t*Bu_2_Im, *i*Pr_2_Im^Me^, *i*Pr_2_Im) and [W(CO)_5_(L)] (L=Dipp_2_NHSi, Xyl_2_NHSi, Dipp_2_Im, Cy_2_Im, *i*Pr_2_Im^Me^, *i*Pr_2_Im). Note that *V*_bur_% has been calculated for different distances for the 3d element nickel and the 5d element tungsten.

	[(L)Ni(CO)_3_]^[a]^	[(L)W(CO)_5_]^[b]^
Dipp_2_NHSi	35.5^[c]^	27.4
Xyl_2_NHSi	–	24.8
Dipp_2_Im	34.8 31.5^[c]^	24.7
Mes_2_Im	32.2	–
Cy_2_Im	–	19.6
*t*Bu_2_Im	40.4^[d]^	–
*i*Pr_2_Im^Me^	28.8	20.2
*i*Pr_2_Im	28.2^[e]^	19.6

[a] r=3.0 Å, *d*=2.0 Å; [b] r=3.5 Å, *d*=2.5 Å; [c] optimized structure; [d] [(L)Ni(CO)_2_]; [e] average value of L in [(L)_2_Ni(CO)_2_].

The calculated percent buried volume of Dipp_2_NHSi for the nickel complex is 35.5 % and for the tungsten complex it is 27.4 % which, in both cases, is notably higher than those calculated for the xylyl‐substituted NHSi (W: 24.8 %) and the aryl substituted carbenes Dipp_2_Im (Ni: 34.8 %, W: 24.7 %) and Mes_2_Im (Ni: 32.2 %). This trend can be observed for all NHCs listed in Table 5 with exception of the extremely bulky *tert*‐butyl‐substituted carbene. However, it is important to note that the NHSi ligand Dipp_2_NHSi seems to be bulkier (in terms of the volume it buries) than the NHC analogue Dipp_2_Im, despite the larger M−Si distance in the silylene complexes compared to the M−C distance in carbene complexes.

The ^13^C NMR spectra of monosubstituted, octahedral tungsten carbonyl complexes [W(OC)_5_(L)] have been used to evaluate the donor strengths of the ligand L. Buchner and Schenk established the *trans*‐influence series of different ligands L toward tungsten(0) on the basis of ^1^ *J*(^183^W–^13^C) coupling constants of the carbonyl group *trans* to L. ^13^C NMR spectra and the associated ^1^ *J*(^183^W–^13^C) coupling constants are proposed to be useful tools to gain insight into the *trans*‐influence of a large variety of ligands towards tungsten(0). A good *σ*‐donor ligand L, which forms a strong, short bond to the metal center demands a high degree of metal orbitals of *n*s‐ and (*n‐1*)d‐character for this bond. Therefore, for the bond to the *trans*‐ligand L’, less metal s‐ and d‐character, and more p‐character remains, which results in an increase of the M−L’ bond lengths and a decrease in one‐bond spin coupling data, for example, the ^1^ *J*(^183^W–^13^C) coupling constants. Thus, the reduction of one‐bond spin coupling constants was rationalized and is directly related to the s‐character of the hybrid orbitals used by both atoms in the formation of their bond to L and *trans*‐CO.^40^ The magnitude of a spin–spin coupling constant across one bond is dominated by the Fermi contact term. In a series of closely related compounds considering the same type of bond (here W−CO), it is usually assumed that other factors change very little and that the variations in ^1^ *J*(A–B) are mainly due to changes in the s‐character of the bonding hybrid orbitals at the metal atoms. Thus, a conclusion regarding the *σ*‐donor capability of the ligand L can be made, and a series of examples are given in Table 6 which demonstrate that the stronger σ‐donor ligand L leads to a smaller coupling constant ^1^ *J*(^183^W–^13^C)_*trans*_.

**Table 6:** ^1^ *J*(^183^W‐^13^C) coupling constants of [M(CO)_5_L] (L=H^−^, CN^−^, Ph_3_As, Ph_3_Sb, Ph_3_P, Cl^−^, Br^−^, I^−^) of the *cis* and *trans*‐standing carbonyl ligands.

Ligand	*cis*‐CO Δ [ppm], ^1^ *J*_W–C_ [Hz]	*trans*‐CO Δ [ppm], ^1^ *J*_W–C_ [Hz]
H^−^	205.9 124	210.3 149
CN^−^	197.6 124	200.2 139
Ph_3_P	197.2 126	199.0 140
Ph_3_As	196.7 126	199.0 155
Ph_3_Sb	196.1 124	198.2 162
Cl^−^	199.6 128	201.4 165
Br^−^	198.6 127	201.5 171
I^−^	197.1 127	201.6 176

In this series of complexes [W(CO)_5_(L)] (L=H^−^, CN^−^, Ph_3_P, Ph_3_As, Ph_3_Sb, Cl^−^, Br^−^, I^−^; Table 6) the ^1^ *J*(^183^W–^13^C)_*cis*_ coupling constant remains remarkably constant, lying between 124 and 128 Hz and shows only a small variation of 4 Hz for these very different ligands L. On the other hand, ^1^ *J*(^183^W–^13^C)_*trans*_ reveals a variation of approximately 35 Hz and, from the data in Table 6, the ligands L may be arranged in a series of increasing ^1^ *J*(^183^W–^13^C)_*trans*_ of the axial carbonyl group and a decreasing *trans*‐influence in the octahedral tungsten carbonyl complexes.

Evaluation of the ^13^C{^1^H} NMR spectrum of **5** revealed tungsten satellites for the *trans*‐ and *cis*‐CO resonances with ^1^ *J*(^183^W–^13^C) coupling constants of 144 Hz (*trans*‐CO) and 121 Hz (*cis*‐CO), which fit well with the data presented in Table 6. Furthermore, ^1^ *J*(^183^W–^13^C)_*trans*_ obtained for **5** is close to ^1^ *J*(^183^W–^13^C)_*trans*_ of other silylene tungsten pentacarbonyl complexes reported previously, that is, [W(CO)_5_(Xyl_2_NHSi)] **VI** 6f with a coupling constant ^1^ *J*(^183^W–^13^C)_*trans*_ of 144 Hz and [W(CO)_5_(Amid_2_NHSi)] **VII** 41 (Amid_2_NHSi=bis(amidinato)silylene) with a coupling constant of 145 Hz. The ^1^ *J*(^183^W–^13^C)_*trans*_ coupling constants of these complexes lies between the values found for [W(CO)_5_(PPh_3_)] (140 Hz) and [(Me_3_P)W(CO)_5_] (145 Hz).^40^

For the carbene complexes **6**–**8** as well as for ([W(CO)_5_(Dipp_2_Im)]) **V** 42 reported previously, the values of ^1^ *J*(^183^W–^13^C) _*trans*_ lie in a range between 126 and 132 Hz and, thus, are much lower than those of the silylene complexes. These values demonstrate that NHCs are superior net donors (including electrostatic and orbital contributions) and reveal a stronger *trans*‐influence compared to the silylene (and phosphine) ligands. They lie in the region of the excellent donor ligand hydride (i.e. [W(CO)_5_(H)] ^−^, ^1^ *J*(^183^W–^13^C)_*trans*_=149 Hz). The better net donor properties of the NHC ligands are also reflected in the IR spectra of the complexes. The C−O stretching frequencies of different tungsten complexes with *N‐*heterocyclic carbenes and *N‐*heterocyclic silylenes are shown in Table 7. In complexes of the type [W(CO)_5_(L)] with the idealized *C*_4*v*_ symmetry, A_1_^I^, E and A_1_^II^ stretching vibrations are IR‐active. The A_1_^I^ vibration is the symmetric stretching mode of all carbonyl groups in *cis* and *trans* positions and can thus be used as a probe of the total charge density at the tungsten atom. The E and A_1_^II^ stretches are close in energy and often not resolved in the IR spectra of the compounds. For the silylene complexes, the A_1_^I^ frequencies are slightly higher (see Table 7, **5**: 2068 cm^−1^; Amid_2_NHSi: 2069 cm^−1^) than those observed for the corresponding carbene compounds (2053–2058 cm^−1^), which indicates that less electron density is located at the tungsten atom due to the poorer net donor properties and/or better net acceptor properties of the NHSi ligand.

**Table 7:** ^1^ *J*(^183^W‐^13^C) coupling constants of [M(CO)_5_(L)] (L=Dipp_2_NHSi, Xyl_2_NHSi, Amid_2_NHSi, *i*Pr_2_Im, *i*Pr_2_Im^Me^, Me_2_Im^Me^, Dipp_2_Im) of the *cis*‐ and *trans*‐standing carbonyl ligands.

Ligand complex	*cis*‐CO Δ [ppm], ^1^ *J*_W–C_ [Hz]	*trans*‐CO Δ [ppm], ^1^ *J*_W–C_ [Hz]	*ν(CO)* [cm^−1^]
Dipp_2_NHSi **5**	193.6 120.5	196.6 143.9	1935, 2068
Xyl_2_NHSi VI	193.7 120.8	196.6 144.3	1980, 2011, 2069
Amid_2_NHSi VII	203.7 123.1	203.3 145.0	–
*i*Pr_2_Im **6**	197.7 126.0	204.1 126.1	1961, 2056
*i*Pr_2_Im^Me^ **7**	197.9 126.2	201.9 131.9	1998, 2058
Me_2_Im^Me^ **8**	198.5 125.9	201.6 131.6	1864, 2058
Dipp_2_Im V	197.2 125.8	200.8 127.2	1916, 2053^[a]^

[a] in CHCl_3_.

Analysis of the ^1^ *J*(^183^W–^13^C) coupling constants thus reveals that the net donor properties of the NHSi ligand should be similar to phosphines, but not to NHCs; the latter can be classified as excellent (*σ*‐)donors such as hydride or methyl. However, it should be noted that several mechanisms, which are not independent from each other, may contribute to the *trans*‐influence which includes orbital energy separation between the tungsten acceptor orbital and the ligand donor orbital, changes in overlap population, interaction with tungsten *n*p orbitals and *π*‐contributions, etc.

To support these findings, we analyzed the tungsten carbonyl model complexes [W(CO)_5_(Me_2_Im)] and [W(CO)_5_(Me_2_NHSi)] at the TZ2P/BLYP/ZORA/D3(BJ) level of theory more closely. Calculated IR stretching frequencies of the complexes [W(CO)_5_(Me_2_Im)] and [W(CO)_5_(Me_2_NHSi)] and Voronoi deformation density (VDD) charges of tungsten are given in Table 8, and the energy decomposition analysis (kJ mol^−1^) of the axial tungsten–carbonyl bond in the complexes [W(CO)_5_(Me_2_Im)] and [W(CO)_5_(Me_2_NHSi)] is given in Table 9. Inspection of the calculated IR stretching vibrations of *pseudo*‐A_1_ symmetry reveals the same trend, that is, that the symmetric stretching frequency for the NHSi complexes lies 10 cm^−1^ higher in energy and supports the idea that the NHC ligand is the better net donor ligand. The EDA of the *trans*‐CO ligand (Table 9) reveals that the W−C orbital interaction is stronger for [W(CO)_5_(Me_2_Im)] (−380.5 kJ mol^−1^) than for [W(CO)_5_(Me_2_NHSi)] (−365.1 kJ mol^−1^), which in turn supports the idea of a larger orbital interaction of the *trans*‐NHSi ligand. Interestingly, for both complexes we calculated similar contributions from *σ*‐symmetry (−162.5 kJ mol^−1^ for [W(CO)_5_(Me_2_Im)] and −165.5 kJ mol^−1^ for [W(CO)_5_(Me_2_NHSi)]), whereas *π*‐back‐bonding is more efficient for the NHC complex (−218.0 kJ mol^−1^ vs. −200.7 kJ mol^−1^ for the Me_2_NHSi complex), which is in line with the observed and calculated IR spectra.

**Table 8:** A_1_ IR stretching frequencies (in cm^‐1^) of [W(CO)_5_(Me_2_Im)] and [W(CO)_5_(Me_2_NHSi)] complexes and Voronoi deformation density (VDD) charges (as fraction of one electron) of W. Negative VDD charge values signify accumulation of electrons (C_2V_ symmetry).

L	VDDC (W)	*ν(CO)*
Me_2_Im	+0.131	1902 (s), 1916 (m), 2023 (w)
Me_2_NHSi	+0.100	1938 (s), 2033 (m)

**Table 9:** Energy Decomposition Analysis (kJ mol^−1^) of the axial tungsten‐carbonyl bond in [W(CO)_5_(Me_2_Im)] and [W(CO)_5_(Me_2_NHSi)] complexes.

L‐W	Me_2_Im	Me_2_NHSi
Δ*E*_int_	−242.1	229.2
Δ*E*_Pauli_	592.9	580.8
Δ*V*_elstat_	−429.7	−421.1
Δ*E*_disp_	−24.8	−23.8
Δ*E*_oi_	−380.5	−365.1
Δ*E*_oi_^σ^	−162.5	−164.5
Δ*E*_oi_^π^	−218.0	−200.7
Δ*E*_oi_^δ^	0.0	0.0

**Scheme 9:**
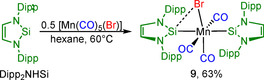
Synthesis of [Mn(CO)_3_(Dipp_2_NHSi)_2_(Br)] **9**.

Silylenes reveal a considerable affinity towards halogens and halogen‐containing compounds which has been reported, with examples being mainly in main group element chemistry.^43^ One example of such halophilic reactions is the insertion of *t*Bu_2_NHSi into the C−X (X=Cl, Br) bond of chloro‐ and bromocarbons.^44^ This behavior can also be observed in transition metal chemistry, as exemplified by Tilley and co‐workers in the reactivity of the previously mentioned dinuclear ruthenium complex [{(*η* ^5^‐C_5_Me_5_)_2_Ru}_2_(H)(*μ*‐H)(*μ*,*η* ^2^‐HSiRCl)(*μ*‐Cl)(*μ*,*η* ^2^‐*t*Bu_2_NHSi)] (R=Ph, *n*‐hexyl), in which an NHSi‐Cl ligand bridges two ruthenium atoms *via* the silicon and chloride atoms (Scheme 3). After our reactions of NHSi with suitable carbonyl precursors [M(CO)_5_(THF)] leading to NHSi carbonyl complexes, we were interested to see how this reaction pattern changes if the transition metal complex contains a halide ligand in addition to the carbonyl ligands. Natural starting materials for such studies are group 7 metal carbonyl halide complexes [M(CO)_5_(X)], and thus we reacted Dipp_2_NHSi with [Mn(CO)_5_(Br)], which led to a low yield conversion of [Mn(CO)_5_(Br)] (Scheme 9). The reaction of two equivalents of Dipp_2_NHSi with [Mn(CO)_5_(Br)] in hexane at room temperature then afforded the bis‐silylene complex **9**, which is very soluble in common organic solvents such as THF and toluene, but only sparingly soluble in non‐polar solvents such as hexane. It can be isolated by filtration from the latter solvent as a yellow solid in 51 % yield. [Mn(CO)_3_(Dipp_2_NHSi)_2_(Br)] **9** was characterized by IR, NMR spectroscopy, elemental analysis and single‐crystal X‐ray diffraction. Elemental analysis performed on crystals of **9** led to the assumption that two of the carbonyls of the manganese complex were replaced by NHSi ligands. As the IR spectrum revealed only two absorptions for the CO stretching modes at 1927 and 1962 cm^−1^ this assumption was confirmed by the loss of two carbonyl ligands. Interestingly, there are only a few publications on analogous manganese complexes bearing *N*‐heterocyclic carbene ligands. In 1977, Lappert *et al*.29a reported that the reaction of [Mn(CO)_5_(Br)] with the *N‐*heterocyclic carbene dimer (Me_2_Im^H2^)_2_ led to oxidation of the manganese compound instead of the formation of the desired complex [Mn(CO)_3_(Me_2_Im^H2^)_2_(Br)]. By using the manganese bis‐phosphine precursor [Mn(CO)_3_(PPh_3_)_2_(Br)], the bis‐carbene complex *fac*‐[Mn(CO)_3_(Me_2_Im)_2_(Br)] was obtained in very low yields *via* substitution of both phosphine ligands. Whittlesey and co‐workers reported the reaction of two equivalents of the backbone‐methylated NHC *i*Pr_2_Im^Me^ with [Mn(CO)_5_(Br)] which afforded the bis‐NHC complex *fac*‐[Mn(CO)_3_(*i*Pr_2_Im^Me^)_2_(Br)]. The reaction of one equivalent of the sterically more demanding NHC Dipp_2_Im led to the mono‐NHC complex [Mn(CO)_4_(Dipp_2_Im)(Br)]^45^ which is also known for the *N‐*mesityl‐substituted NHC Mes_2_Im.^46^

**Figure 4:**
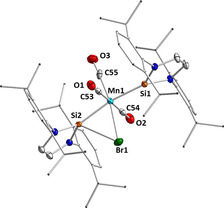
Molecular structure of [Mn(CO)_3_(Dipp_2_NHSi)_2_(Br)] **9** in the solid state (ellipsoids drawn at 50 % probability; hydrogen atoms omitted for clarity). Selected bond lengths [Å] and angles [°]: Mn1−C53 1.854(3), C53−O1 1.133(4), Mn1−C54 1.852(3), C54−O2 1.142(4), Mn1−C55 1.839(3), C55−O3 1.084(4), Mn1−Br1 2.5585(5), Si1−Br1 3.6315(9), Si2−Br 2.7583(8), Si2‐Mn1‐Br1 69.95(2), Si1‐Mn1‐Br1 98.34(3), Si2‐Br1‐Mn1 49.43(2), Br1‐Si2‐Mn1 60.62(2), Si1‐Mn1‐Si2 168.23(4), plane (Mn1‐C53‐C54‐C55)/ plane (C53‐C54‐Br) 10.372(14)°.

**Figure 5:**
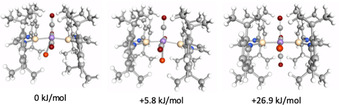
DFT calculations (TURBOMOLE/def2‐TZVP(Mn,Si,Br)/def2‐SV(P)/BP86‐D3(BJ)) on [Mn(CO)_3_(Dipp_2_NHSi)_2_(Br)] **9**.

The ^29^Si NMR spectrum of **9** recorded at −40 °C reveals a single resonance at 121.5 ppm which is in line with a dynamic behavior of this complex in solution, caused by an oscillating movement of the bromine atom between the two silicon silylene atoms and explained in detail in the next paragraph (see also Figure 5). Single crystals of complex **9** were grown by slow evaporation of a benzene solution at room temperature, and the molecular structure of **9** was established by X‐ray diffraction (Figure 4). Compound **9** crystallizes in the orthorhombic space group *P*2_1_2_1_2_1_ and has a distorted octahedral structure with the silylene ligands *trans* to one another and the three CO ligands and the bromine atom lying in a plane between them. The silylenes are simultaneously bent towards the bromine atom which lies slightly out of the plane formed by the three carbonyl groups (10.37(14)°). Despite the interaction of one silylene ligand with the bromine atom, nearly the same bond lengths for Mn−Si1 (2.2329(9) Å) and Mn−Si2 (2.2304(9) Å) are observed. The Si2−Br1 distance is 2.7583(8) Å whereas the Si1−Br1 distance is 3.6315(9) Å.

The distortion of the complex is caused by an interaction of the lone pair orbitals at the bromide ligand with the unoccupied silicon p_*π*_‐orbital. DFT calculations on **9** show that the Si−Br interaction contributes to the stability of the complex and that the bromide ligand should oscillate between the two silylene silicon atoms. A more symmetrical arrangement with the bromide ligand in the manganese carbonyl plane (i.e. without significant interaction with the silicon atom) is energetically unfavorable by 5.8 kJ mol^−1^ (TURBOMOLE/def2‐TZVP(Mn,Si,Br)/def2‐SV(P)/BP86‐D3(BJ)) and represents a transition state (see Figure 5). However, it should be noted that the relative position of the bromide ligand with respect to the five‐membered ring of the silylene ligand is also crucial for stabilization of the complex. If the bromide atom lies perpendicular to the plane spanned by the five‐membered NHSi rings and interaction with the silicon atom p_π_‐orbital is enabled, the complex is stabilized, whereas if the bromide lies in the NHSi plane, such an interaction is not possible, leading to a destabilization of 26.9 kJ mol^−1^ with respect to the minimum energy structure (Figure 5).

**Scheme 10:**
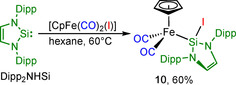
Synthesis of [(*η* ^5^‐C_5_H_5_)Fe(CO)_2_(Dipp_2_NHSi‐I)] **10**.

In contrast to **9**, reaction of Dipp_2_NHSi with [(*η* ^5^‐C_5_H_5_)Fe(CO)_2_(I)] in hexane at room temperature led to formation of the iron complex [(*η* ^5^‐C_5_H_5_)Fe(CO)_2_(Dipp_2_NHSi‐I)] (**10**), which is formally the product of an insertion of the silylene into the Fe−I bond (Scheme 10). We have no evidence to indicate that initial substitution of CO by Dipp_2_NHSi is involved in the course of this reaction. After purification, complex **10** was obtained as a red‐brown solid in 60 % yield and was characterized by IR and NMR spectroscopy and single‐crystal X‐ray diffraction. The IR spectrum revealed two absorptions for the symmetric and asymmetric stretches of the CO ligands at 1974 and 2021 cm^−1^, respectively, which confirms that no carbonyl ligand was lost.

The shift of the stretching modes of complex **10** from those of the starting material [(*η* ^5^‐C_5_H_5_)Fe(CO)_2_(I)] (1986 and 1941 cm^−1^)^47^ displays the altered electronic environment at the iron atom. By symmetry (maximum=C_s_), the aryl‐substituents of the silylene ligand are chemically inequivalent which leads to a splitting of their resonances in the ^1^H and ^13^C NMR spectra. The CO ligands give rise to one resonance at 212.2 ppm, and the resonance of the silicon atom was detected at 17.2 ppm in the ^29^Si NMR spectrum, significantly shifted (59 ppm) to higher fields compared to the free NHSi. The reaction of [(*η* ^5^‐C_5_H_5_)Fe(CO)_2_(I)] with *N*‐heterocyclic carbenes leads to formation of the ionic complexes [(*η* ^5^‐C_5_H_5_)Fe(CO)_2_(NHC)][I] (NHC=Me_2_Im, *i*Pr_2_Im, Mes_2_Im, etc.) by displacement of iodide.^48^ In contrast to the formation of **10**, the carbene ligands are not prone to nucleophilic attack by iodide, which is in line with the properties of the NHSi as discussed above.

**Figure 6:**
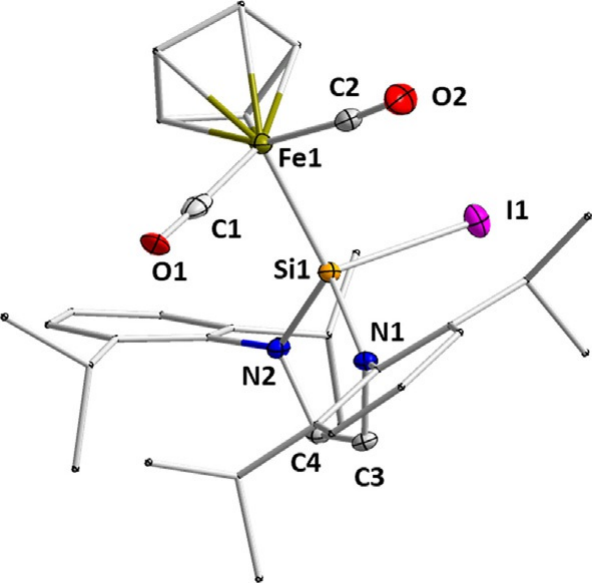
Molecular structure of **10** in the solid state (ellipsoids drawn at 50 % probability; hydrogen atoms omitted for clarity). Selected bond lengths [Å] and angles [°]: Fe−Si1 2.2461(9), Si1−I1 2.6443(9), Si1−N1 1.746(3), Si1−N2 1.744(3), Fe1−C32 1.767(4), Fe1−C33 1.760(4), C32−O1 1.105(4), C33−O2 1.135(4); plane (N2‐C1‐C2‐N2)/ plane (N1‐Si1‐N2) 20.970(139)°.

Compound **10** crystallizes as dark red crystals in the monoclinic space group *P*2_1_/*n* (Figure 6). The X‐ray diffraction analysis confirmed the insertion of the silicon atom into the iron‐iodine bond leading to an oxidized silylene ligand which no longer acts as a neutral two‐electron donor but as a silyl ligand, i.e., an anionic 2‐VE donor ligand, which gives complex **10** an 18 electron count. The silyl ligand is tetrahedrally surrounded by iron, iodine and two nitrogen atoms with a silicon‐iodine distance of 2.6443(9) Å and a silicon–iron bond length of 2.2461(9) Å. The tetrahedral coordination at silicon leads to a twist out of the (former) NHSi plane containing the nitrogen atoms and the backbone by 20.97(14)°). There is no significant geometrical change at the [(*η* ^5^‐C_5_H_5_)Fe(CO)_2_] unit.

## Conclusions

We report here some peculiarities of *N‐*heterocyclic silylenes as ligands, in particular for the Dipp_2_NHSi ligand, which is the silicon analogue of the widely used NHC ligand Dipp_2_Im. To our surprise, the silylene ligand Dipp_2_NHSi is much less reactive compared to similar NHC ligands. Calculations on the main electronic features of Me_2_Im/Me_2_NHSi and Dipp_2_NHSi/ Dipp_2_Im revealed significant differences in the frontier orbital region of these compounds, which affect the ligation properties of NHSis. (i) The orbital order changes, as the HOMO of NHSis is a *π*‐orbital. (ii) The *σ*‐orbital of NHSis lies at much lower energy than those of NHCs and has a higher degree of s‐orbital character. (iii) The *π*‐accepting LUMO of NHSis is much lower in energy than those of NHCs and reveals a large silicon p_x_‐contribution.

The bonding of Me_2_Im and Me_2_NHSi (=L) to transition metal complexes has been assessed for several model systems, i.e., [Ni(L)], [Ni(CO)_3_(L)], and [W(CO)_5_(L)]. These studies reveal some common features in the difference between M–NHSi and M–NHC bonding: (i) NHCs are the better net donor ligands. (ii) The intrinsic M−L interaction energy typically decreases going from M–NHC to M–NHSi. (iii) This decrease is mainly caused by favorable electrostatic contributions to the M−NHC bond. (iv) The orbital interaction in the carbonyl complexes was typically larger for M–NHSi than for M–NHC. (iv) The contribution of *σ*‐ and *π*‐interactions depends significantly on the system under investigation. Interestingly, the M–NHSi *π*‐interaction is often stronger compared to that in M–NHC. (v) The electronic properties of NHSi ligands are closer to those of phosphines than to NHCs. (vi) Calculation of the percent buried volume (*V*_bur_%) show that Dipp_2_NHSi is slightly bulkier than Dipp_2_Im.

We have shown that Dipp_2_NHSi reacts with [Ni(CO)_4_] to form a colorless intermediate [Ni(CO)_3_(Dipp_2_NHSi)] (**1**), which led to the isolation of the dinuclear silylene‐bridged complex [{Ni(CO)_2_(*μ*‐Dipp_2_NHSi)}_2_] (**2**) upon CO elimination. It is interesting to note that the corresponding mononuclear carbene complex [Ni(CO)_3_(Dipp_2_Im)] is stable, whereas **1** loses CO easily and dimerizes to the NHSi‐bridged compound [{Ni(CO)_2_(*μ*‐Dipp_2_NHSi)}_2_] (**2**), which is favorable according to DFT calculations. To provide experimental support for our calculations (*vide supra*), the silylene complexes [M(CO)_5_(Dipp_2_NHSi)] (M=Cr **3**, Mo **4**, W **5**) were synthesized from Dipp_2_NHSi and [M(CO)_6_] (M=Cr, Mo, W) and the tungsten NHSi complexes were compared to the NHC complexes [W(CO)_5_(*i*Pr_2_Im)] (**6**), [W(CO)_5_(*i*Pr_2_Im^Me^)] (**7**)^37^ and [W(CO)_5_(Me_2_Im^Me^)] (**8**).^37^ Reaction of Dipp_2_NHSi with the manganese carbonyl complex [Mn(CO)_5_(Br)] led to [Mn(CO)_3_(Dipp_2_NHSi)_2_(Br)] (**9**), for which X‐ray crystallography and DFT calculations revealed an intramolecular stabilizing donor‐acceptor interaction between the bromide ligand lone pair orbitals and the silylene acceptor orbital. In solution, the system is fluxional, and the bromide ligand switches between two possible Br−Si interactions with a symmetric arrangement as a transition state for the process. Complete transfer of a halide to the silylene was achieved for the reaction of Dipp_2_NHSi with [(*η* ^5^‐C_5_H_5_)Fe(CO)_2_(I)] which led to the formation of [(*η* ^5^‐C_5_H_5_)Fe(CO)_2_(Dipp_2_NHSi‐I)] (**10**), a complex that no longer features a silylene ligand but contains a silyl ligand due to the formal oxidation of the silicon atom.

In summary, we have shown that *N‐*heterocyclic silylenes, the higher homologues of the now ubiquitous NHC ligands in transition metal chemistry, show significantly different behavior regarding their coordination chemistry. These differences can largely be explained by the simple MO picture of the NHSis. In particular, one energetically low lying *π*‐acceptor orbital seems to determine the coordination chemistry of these ligands and is responsible for silylenes being good bridging ligands, showing intramolecular interactions with donors such as halides, and being good intramolecular acceptors for migrating groups such as halide ligands.

## Experimental Section

### General procedures

All reactions and subsequent manipulations were performed under an argon atmosphere in an Innovative Technology Inc. glovebox or using standard Schlenk techniques. NMR spectra were recorded on Bruker Avance 300, Bruker NEO 400 or Bruker Avance 500 spectrometers in C_6_D_6_, [D_8_]THF, CDCl_3_ and [D_8_]toluene solutions at room temperature if not stated differently. Chemical shifts are listed in parts per million (ppm) and were calibrated against the residual solvent signals (δ (^1^H): C_6_D_5_H 7.16, d^7^‐THF 3.58, C(D/H)Cl_3_ 7.26, d^7^‐toluene 2.08,; δ (^13^C): C_6_D_6_ 7128.06, [D_8_]‐THF 67.21, CDCl_3_ 77.16, [D_8_]‐toluene 20.43). Coupling constants are quoted in Hz. Infrared spectra were recorded on solid samples at room temperature on a Bruker Alpha FT‐IR spectrometer using an ATR unit and are reported in cm^−1^. Elemental analyses were performed in the micro analytical laboratory of the Institute of Inorganic Chemistry at the University of Würzburg with an Elementar vario MICRO cube. Dipp_2_NHSi,6f, 49 [Mn(CO)_5_(Br)]^50^ and [(*η* ^5^‐C_5_H_5_)Fe(CO)_2_(I)]^51^ were prepared according to literature procedures. All other starting materials were purchased from commercial sources and used without purification. All solvents were HPLC grade, further treated to remove traces of water using an Innovative Technology Inc. Pure‐Solv Solvent Purification System and deoxygenated using the freeze‐pump‐thaw method. For irradiation, a mercury vapor lamp with a wavelength of *λ*=254 nm was used.

### [{Ni(CO)_2_(*μ*‐Dipp_2_NHSi)}_2_] (2)

**Safety precautions in handling [Ni(CO)_4_]**: Special care has been taken while manipulating the extremely toxic, flammable and volatile (b.p. 43 °C) [Ni(CO)_4_]. All manipulations were carried out in a well‐ventilated fume hood or a glovebox. Safety glasses, an apron and gloves using additional protective gloves should be worn when handling this reagent. [Ni(CO)_4_] should be maintained at temperatures below 0 °C. Traces of [Ni(CO)_4_] can be disposed of by treatment with concentrated nitric acid diluted 1:1 with water and all glassware used should be treated with the nitric acid solution. Dipp_2_NHSi (150 mg, 370 μmol) dissolved in toluene (10 mL) was cooled to 0 °C and Ni(CO)_4_ (53.0 *μ*L, 69.5 mg, 407 μmol, 1.1 equiv.) was added. The reaction mixture was allowed to warm to room temperature and the resulting light yellow solution stirred for 16 h. All volatiles were removed under reduced pressure yielding **2** (83.0 mg, 160 μmol, 43 %) as a dark violet solid. Crystals suitable for X‐ray diffraction were grown by slow evaporation of a saturated benzene solution of **2** at room temperature. ^1^H NMR (300.1 MHz, C_6_D_6_): δ=7.23–7.12 (m, 6 H, aryl‐C*H*), 6.38 (s, 2 H, C*H*), 3.30 (sept, 4 H, ^3^ *J*_H‐H_=6.9 Hz, *i*Pr‐C*H*), 1.33 (d, 12 H, ^3^ *J*_H‐H_=6.9 Hz, *i*Pr‐C*H*_3_), 1.18 (d, 12 H, ^3^ *J*_H‐H_=6.9 Hz, *i*Pr‐C*H*_3_). ^13^C{^1^H} NMR (75.5 MHz, C_6_D_6_): δ=195.7 (*C*O), 146.0 (aryl‐*C*_ipso_), 137.5 (aryl‐*C*_ortho_), 128.5 (aryl‐*C*_meta_), 125.4 (aryl‐*C*_para_), 123.9 (N*C*CN), 29.1 (*i*Pr‐*C*H_3_), 24.9 (*i*Pr‐*C*H_3_), 24.2 (*i*Pr‐*C*H_3_). ^29^Si{^1^H} NMR (59.6 MHz, C_6_D_6_): δ=121.9. IR (CH_2_Cl_2_ [cm^−1^]): 1971 (s, *ν*_CO,str_), 2010 (vs., *ν*_CO,str_). Elemental analysis (%) calcd for C_56_H_72_N_4_Si_2_Ni_2_O_4_: C 64.75, H 6.99, N 5.39; found C 63.97, H 6.92, N 5.30.

**[Cr(CO)_5_(Dipp_2_NHSi)] (3)**: [Cr(CO)_6_] (70.0 mg, 318 μmol) dissolved in THF (10 mL) was irradiated for 3 h at room temperature. After addition of Dipp_2_NHSi (129 mg, 318 μmol) in THF (5 mL), the reaction mixture was stirred at room temperature for 1 h. All volatiles were removed under reduced pressure and the residue was washed with hexane and dried in vacuo yielding **3** (140 mg, 235 μmol, 74 %) as an orange solid. ^1^H NMR (400.3 MHz, C_6_D_6_): δ=7.21–7.11 (m, 6 H, aryl‐C*H*), 6.32 (s, 2 H, C*H*), 3.34 (sept, 4 H, ^3^ *J*_H H_=6.8 Hz, *i*Pr‐C*H*), 1.38 (d, 12 H, ^3^ *J*_H‐H_=6.8 Hz, *i*Pr‐C*H*_3_), 1.17 (d, 12 H, ^3^ *J*_H‐H_=6.8 Hz, *i*Pr‐C*H*_3_). ^13^C{^1^H} NMR (100.7 MHz, C_6_D_6_): δ=215.5 (*C*O), 211.6 (*C*O), 146.0 (aryl‐*C*_ipso_), 137.1 (aryl‐*C*_ortho_), 129.0 (aryl‐*C*_meta_), 126.8 (aryl‐*C*_para_), 124.1 (N*C*CN), 29.3 (*i*Pr‐*C*H_3_), 25.5 (*i*Pr‐*C*H_3_), 23.7 (*i*Pr‐*C*H_3_). ^29^Si{^1^H} NMR (79.5 MHz, C_6_D_6_): δ=138.2. IR ([cm^−1^]): 1937 (vs., *ν*_CO,str_), 2060 (m, *ν*_CO,str_), 2867 (w, *ν*_CH,str_), 2927 (w, *ν*_CH,str_), 2961 (m, *ν*_CH,str_). Elemental analysis (%) calcd for C_31_H_36_N_2_SiO_5_Cr: C 62.40, H 6.08, N 4.69; found C 62.39, H 6.93, N 4.44.

**[Mo(CO)_5_(Dipp_2_NHSi)] (4)**: [Mo(CO)_6_] (75.0 mg, 284 μmol) dissolved in THF (10 mL) was irradiated for 3 h at room temperature. After addition of Dipp_2_NHSi (115 mg, 284 μmol) in THF (5 mL), the reaction mixture was stirred at room temperature for 1 h. All volatiles were removed under reduced pressure and the residue was washed with hexane and dried in vacuo yielding **4** (136 mg, 212 μmol, 75 %) as an orange solid. Crystals suitable for X‐ray diffraction were grown from a saturated hexane solution of **4** at −30 °C. ^1^H NMR (400.3 MHz, CDCl_3_): δ=7.38–7.27 (m, 6 H, aryl‐C*H*), 6.58 (s, 2 H, C*H*), 3.21 (sept, 4 H, ^3^ *J*_H H_=6.9 Hz, *i*Pr‐C*H*), 1.34 (d, 12 H, ^3^ *J*_H‐H_=6.9 Hz, *i*Pr‐C*H*_3_), 1.26 (d, 12 H, ^3^ *J*_H‐H_=6.9 Hz, *i*Pr‐C*H*_3_). ^13^C{^1^H} NMR (100.7 MHz, CDCl_3_): δ=204.1 (*C*O), 201.1 (*C*O), 146.0 (aryl‐*C*_ipso_), 136.8 (aryl‐*C*_ortho_), 128.3 (aryl‐*C*_meta_), 126.2 (aryl‐*C*_para_), 123.8 (N*C*CN), 29.1 (*i*Pr‐*C*H_3_), 25.2 (*i*Pr‐*C*H_3_), 23.8 (*i*Pr‐*C*H_3_). ^29^Si{^1^H} NMR (79.5 MHz, CDCl_3_): δ=125.4. IR ([cm^−1^]): 1946 (vs., *ν*_CO,str_), 2070 (m, *ν*_CO,str_), 2866 (w, *ν*_CH,str_), 2927 (w, *ν*_CH,str_), 2961 (m, *ν*_CH,str_). Elemental analysis (%) calcd for C_31_H_36_N_2_SiO_5_Mo: C 58.12, H 5.66, N 4.37; found C 58.77, H 6.63, N 4.03.

**[W(CO)_5_(Dipp_2_NHSi)] (5)**: [W(CO)_6_] (87.0 mg, 247 μmol) dissolved in THF (10 mL) was irradiated for 3 h at room temperature. After addition of Dipp_2_NHSi (100 mg, 247 μmol) in THF (5 mL), the reaction mixture was stirred at room temperature for 1 h. All volatiles were removed under reduced pressure and the residue was washed with hexane and dried in vacuo yielding **5** (148 mg, 203 μmol, 82 %) as a yellow solid. Crystals suitable for X‐ray diffraction were grown from a saturated hexane solution of **5** at −30 °C. ^1^H NMR (500.1 MHz, C_6_D_6_): δ=7.22–7.19 (m, 2 H, aryl‐C*H*), 7.14–7.12 (m, 4 H, aryl‐C*H*), 6.32 (s, 2 H, C*H*), 3.31 (sept, 4 H, ^3^ *J*_H‐H_=6.9 Hz, *i*Pr‐C*H*), 1.38 (d, 12 H, ^3^ *J*_H‐H_=6.9 Hz, *i*Pr‐C*H*_3_), 1.17 (d, 12 H, ^3^ *J*_H‐H_=6.9 Hz, *i*Pr‐C*H*_3_). ^13^C{^1^H} NMR (125.8 MHz, C_6_D_6_): δ=196.4 (^1^ *J*_W‐Si_=144.0 Hz, *trans*‐*C*O), 193.9 (^1^ *J*_W‐Si_=120.5 Hz, *cis*‐*C*O), 146.0 (aryl‐*C*_ipso_), 136.9 (aryl‐*C*_ortho_), 128.9 (aryl‐*C*_meta_), 126.3 (aryl‐*C*_para_), 124.1 (N*C*CN), 29.4 (*i*Pr‐*C*H_3_), 25.2 (*i*Pr‐*C*H_3_), 23.9 (*i*Pr‐*C*H_3_). ^29^Si{^1^H} NMR (99.4 MHz, CDCl_3_): δ=111.2 (^1^ *J*_W‐Si_=168.3 Hz). IR ([cm^−1^]): 1935 (vs., *ν*_CO,str_), 2068 (m, *ν*_CO,str_), 2867 (w, *ν*_CH,str_), 2927 (w, *ν*_CH,str_), 2963 (m, *ν*_CH,str_). Elemental analysis (%) calcd for C_31_H_36_N_2_O_5_SiW: C 51.11, H 4.98, N 3.85; found C 50.98, H 4.86, N 3.64.

**[W(CO)_5_(*i*Pr_2_Im)] (6)**: [W(CO)_6_] (100 mg, 284 μmol) dissolved in THF (10 mL) was irradiated for 3 h at room temperature. After addition of *i*Pr_2_Im (43.2 mg, 284 μmol) in THF (5 mL), the reaction mixture was stirred at room temperature for 1 h. All volatiles were removed under reduced pressure and the residue was washed with hexane and dried in vacuo yielding **6** (116 mg, 244 μmol, 86 %) as a yellow solid. Crystals suitable for X‐ray diffraction were grown from a saturated hexane/toluene solution of **6** at −30 °C. ^1^H NMR (300.1 MHz, [D_8_]‐THF): δ=7.72 (s, 2 H, C*H*), 5.49 (sept, 2 H, ^3^ *J*_H‐H_ = 6.7 Hz, *i*Pr‐C*H*), 1.75 (d, 12 H, ^3^ *J*_H‐H_=6.7 Hz, *i*Pr‐C*H*_3_). ^13^C{^1^H} NMR (75.5 MHz, [D_8_]THF): δ=201.5 (^1^ *J*_C‐W_=131 Hz, *trans*‐CO), 198.4 (^1^ *J*_C‐W_=124 Hz, *cis*‐CO), 175.4 (N*C*N), 119.4 (N*C*CN), 54.9 (*i*Pr‐*C*H), 23.5 (*i*Pr‐*C*H_3_). ^1^H NMR (400.3 MHz, C_6_D_6_): δ=7.72 (s, 2 H, C*H*), 5.49 (sept, 2 H, ^3^ *J*_H‐H_=6.7 Hz, *i*Pr‐C*H*), 1.75 (d, 12 H, ^3^ *J*_H‐H_=6.7 Hz, *i*Pr‐C*H*_3_). ^13^C{^1^H} NMR (100.7 MHz, C_6_D_6_): δ=201.3 (*trans*‐CO), 198.2 (*cis*‐CO), 176.4 (N*C*N), 117.8 (N*C*CN), 54.1 (*i*Pr‐*C*H), 23.3 (*i*Pr‐*C*H_3_). IR ([cm^−1^]): 1961 (m, *ν*_CO,str_), 2056 (m, *ν*_CO,str_), 2935 (w, *ν*_CH,str_), 2972 (w, *ν*_CH,str_). Elemental analysis (%) calcd for C_14_H_16_N_2_O_5_W: C 35.32, H 3.39, N 5.88; found C 34.96, H 3.44, N 5.95.

**[W(CO)_5_(*i*Pr_2_Im^Me^)] (7)**: [W(CO)_6_] (60.0 mg, 171 μmol) dissolved in THF (10 mL) was irradiated for 3 h at room temperature. After addition of *i*Pr_2_Im^Me^ (30.7 mg, 171 μmol) in THF (5 mL), the reaction mixture was stirred at room temperature for 1 h. All volatiles were removed under reduced pressure and the residue was washed with hexane and dried in vacuo yielding **7** (43.1 mg, 85.9 μmol, 50 %) as a yellow solid. Crystals suitable for X‐ray diffraction were grown from a saturated hexane/toluene solution of **7** at −30 °C. ^1^H NMR (500.1 MHz, CDCl_3_): δ=5.57 (sept, 2 H, ^3^ *J*_H‐H_=7.1 Hz, *i*Pr‐C*H*), 2.24 (s, 6 H, C(C*H*_3_)), 1.51 (d, 12 H, ^3^ *J*_H‐H_=7.1 Hz, *i*Pr‐C*H*_3_). ^13^C{^1^H} NMR (125.8 MHz, CDCl_3_): δ=201.9 (^1^ *J*_C‐W_=132 Hz, *trans*‐*C*O), 197.9 (^1^ *J*_C‐W_=126 Hz, *cis*‐*C*O), 177.7 (N*C*N), 126.5 (N*C*CN), 55.7 (*i*Pr‐*C*H), 21.9 (*i*Pr‐*C*H_3_), 10.8 (C(*C*H_3_)). IR ([cm^−1^]): 1998 (m, *ν*_CO,str_), 2058 (m, *ν*_CO,str_), 2934 (w, *ν*_CH,str_), 2977 (w, *ν*_CH,str_). Elemental analysis (%) calcd for C_16_H_20_N_2_O_5_W: C 38.12, H 4.00, N 5.63; found C 37.92, H 3.99, N 5.56.

**[W(CO)_5_(Me_2_Im^Me^)] (8)**: [W(CO)_6_] (60.0 mg, 171 μmol) dissolved in THF (10 mL) was irradiated for 3 h at room temperature. After addition of Me_2_Im^Me^ (21.2 mg, 171 μmol) in THF (5 mL), the reaction mixture was stirred at room temperature for 1 h. All volatiles were removed under reduced pressure and the residue was washed with hexane and dried in vacuo yielding **8** (43.6 mg, 97.3 μmol, 57 %) as a yellow solid. ^1^H NMR (500.1 MHz, CDCl_3_): δ=3.74 (s, 6 H, NC*H*_3_), 2.15 (s, 6 H, NC(C*H*_3_)). ^13^C{^1^H} NMR (125.8 MHz, CDCl_3_): δ=201.6 (^1^ *J*_C‐W_=132 Hz, *trans*‐*C*O), 198.5 (^1^ *J*_C‐W_=126 Hz, *cis*‐*C*O), 177.1 (^1^ *J*_C‐W_=100 Hz, N*C*N), 125.7 (N*C*CN), 37.7 (NC*H*_3_), 10.1 (NC(C*H*_3_)). IR ([cm^−1^]): 1864 (vs., *ν*_CO,str_), 2058 (m, *ν*_CO,str_), 2961 (w, *ν*_CH,str_). Elemental analysis (%) calcd for C_12_H_12_N_2_O_5_W: C 32.17, H 2.70, N 6.25; found C 31.87, H 2.74, N 6.05.

**[Mn(CO)_3_(Dipp_2_NHSi)_2_(Br)] (9)**: Dipp_2_NHSi (60.0 mg, 148 μmol, 2 equiv.) and [Mn(CO)_5_(Br)] (20.4 mg, 74.2 μmol) were dissolved in hexane (5 mL) and stirred at 60 °C for 16 h. The precipitate was collected by filtration, washed with hexane (2x2 mL) and dried in vacuo yielding **9** (47.9 mg, 46.6 μmol, 63 %) as a yellow solid. Crystals suitable for X‐ray diffraction were grown from a saturated hexane solution of **9** at −30 °C. ^1^H NMR (500.1 MHz, [D_8_]toluene, −40 °C): δ=7.13 (s, 3 H, aryl‐C*H*), 7.05 (s, 3 H, aryl‐C*H*), 6.16 (s, 2 H, C*H*), 3.53 (sept, 4 H, ^3^ *J*_H‐H_=6.6 Hz, *i*Pr‐C*H*), 1.37 (d, 12 H, ^3^ *J*_H‐H_=6.6 Hz, *i*Pr‐C*H*_3_), 1.15 (d, 12 H, ^3^ *J*_H‐H_=6.6 Hz, *i*Pr‐C*H*_3_). ^13^C{^1^H} NMR (125.8 MHz, [D_8_]toluene, −40 °C): δ=213.1 (CO), 146.0 (aryl‐*C*_ipso_), 137.1 (aryl‐*C*_ortho_), 129.1 (aryl‐*C*_meta_), 128.2 (aryl‐*C*_para_), 123.8 (N*C*CN), 29.1 (*i*Pr‐*C*H), 25.7 (*i*Pr‐*C*H_3_), 23.6 (*i*Pr‐*C*H_3_). ^29^Si{^1^H} NMR (99.4 MHz, [D_8_]toluene, −40 °C): δ=121.5. IR ([cm^−1^]): 1927 (vs., *ν*_CO,str_), 1962 (m, *ν*_CO,str_), 2865 (w, *ν*_CH,str_), 2925 (w, *ν*_CH,str_), 2959 (m, *ν*_CH,str_). Elemental analysis (%) calcd for C_55_H_72_BrMnN_4_O_3_Si_2_: C 64.25, H 7.06, N 5.45; found C 64.28, H 6.98, N 5.31.

**[(*η*^5^‐C_5_H_5_)Fe(CO)_2_(Dipp_2_NHSi‐I)] (10)**: Dipp_2_NHSi (100 mg, 247 μmol) and [(*η* ^5^‐C_5_H_5_)Fe(CO)_2_(I)] (75.1 mg, 247 μmol) were dissolved in THF (10 mL) and stirred at room temperature for 16 h. After all volatiles were removed under reduced pressure the residue was dissolved in hexane and cooled to −30 °C yielding **10** (104 mg, 147 μmol, 60 %) as a red‐brown solid. Crystals suitable for X‐ray diffraction were grown from a saturated hexane solution of **10** at −30 °C. ^1^H NMR (400.3 MHz, C_6_D_6_): δ=7.25–7.12 (m, 6 H, aryl‐C*H*), 6.14 (s, 2 H, C*H*), 4.78 (sept, 2 H, ^3^ *J*_H‐H_=4.7 Hz, *i*Pr‐C*H*), 4.06 (s, 5 H, C*H*_cp_), 3.60 (sept, 2 H, ^3^ *J*_H‐H_=4.7 Hz, *i*Pr‐C*H*), 1.58 (d, 6 H, ^3^ *J*_H‐H_=4.7 Hz, *i*Pr‐C*H*_3_), 1.37 (d, 6 H, ^3^ *J*_H‐H_=4.7 Hz, *i*Pr‐C*H*_3_), 1.30 (d, 6 H, ^3^ *J*_H‐H_ = 4.7 Hz, *i*Pr‐C*H*_3_), 1.23 (d, 6 H, ^3^ *J*_H‐H_=4.7 Hz, *i*Pr‐C*H*_3_). ^13^C{^1^H} NMR (100.7 MHz, C_6_D_6_): δ=212.2 (*C*O), 149.7 (aryl‐*C*_ipso_), 148.3 (aryl‐*C*_ortho_), 140.2 (aryl‐*C*_ortho_), 125.3 (aryl‐*C*_meta_), 123.4 (aryl‐*C*_para_), 123.1 (N*C*CN), 86.9 (*C*H_cp_), 29.9 (*i*Pr‐*C*H), 29.3 (*i*Pr‐*C*H), 28.1 (*i*Pr‐*C*H_3_), 26.9 (*i*Pr‐*C*H_3_), 24.1 (*i*Pr‐*C*H_3_), 22.9 (*i*Pr‐*C*H_3_). ^29^Si{^1^H} NMR (79.5 MHz, C_6_D_6_): δ=17.2. IR (CH_2_Cl_2_ [cm^−1^]): 1974 (vs., *ν*_CO,str_), 2021 (m, *ν*_CO,str_), 2865 (w, *ν*_CH,str_), 2925 (w, *ν*_CH,str_), 2961 (m, *ν*_CH,str_). Elemental analysis (%) calcd for C_33_H_41_N_2_SiO_2_IFe: C 55.94, H 5.83, N 3.95; found C 55.84, H 6.06, N 5.16.

### Crystallographic details

Crystal data were collected with a Bruker D8 Apex‐2 diffractometer equipped with an Oxford Cryosystems low‐temperature device using a CCD area detector and graphite monochromated Mo‐*Κ*_α_ radiation or a Rigaku XtaLAB Synergy‐DW diffractometer equipped with an Oxford Cryo 800 using a HyPix‐6000HE detector and copper monochromated Cu‐*Κ*_α_ radiation. Crystals were immersed in a film of perfluoropolyether oil on a MicroMount^TM^ and data were collected at 100 K. The images were processed with the Bruker or CrySalis software packages and the structures solved using the ShelXTL software package.^52^ All non‐hydrogen atoms were refined anisotropically. Hydrogen atoms were included in structure factor calculations and assigned to idealized positions.

Deposition Numbers 1987076 (**2**), 1987080 (**4**), 1987077 (**5**), 1987081 (**6**), 1987079 (**7**), 1987082 (**9**) and 1987078 (**10**) contain the supplementary crystallographic data for this paper. These data are provided free of charge by the joint Cambridge Crystallographic Data Centre and Fachinformationszentrum Karlsruhe Access Structures service www.ccdc.cam.ac.uk/structures.

**Crystal data for [{Ni(CO)_2_(*μ*‐Dipp_2_NHSi)}_2_] (2)**: C_56_H_72_N_4_Ni_2_O_4_Si_2_, M_r_=1038.77, *T*=100.00(10) K, *λ*=1.54184 Å, purple plate, 0.039×0.12×0.215 mm^3^, triclinic space group *P* $\bar{1}$ , *a=*10.5548(2) Å, *b=*11.9880(3) Å, *c=*12.4406(2) Å, *α*=104.912(2)°, *β*=113.089(2)°, *γ*=97.345(2)°, *V*=1352.12(5) Å^3^, *Z*=2, *ρ_calcd_*=1.276 Mg/m^3^, *μ*=1.660 mm^−1^, F(000)=552, 32636 reflections, −13≤h≤13, −14≤k≤15, −13≤l≤15, 3.949<*θ*<77.633°, completeness 97.7 %, 5635 independent reflections, 5193 reflections observed with [I>2*σ*(I)], 315 parameters, 0 restraints, *R* indices (all data) *R*1=0.0399, *wR*2=0.0964, final *R* indices [I>2*σ*(I)] *R*1=0.0350, *wR*2=0.0908, largest difference peak and hole 0.390 and −0.477 eA^−3^, GooF=1.132.

**Crystal data for [Mo(CO)_5_(Dipp_2_NHSi)] (4)**: C_31_H_36_N_2_MoO_5_Si, M_r_=640.68, *T*=100(2) K, *λ*=0.71073 Å, blue plate, 0.066×0.201×0.373 mm^3^, monoclinic space group *P*2_1_/*m*, *a=*9.1296(6) Å, *b=*19.4397(13) Å, *c=*9.5888(6) Å, *β*=110.248(3)°, *V*=1596.62(18) Å^3^, *Z*=1, *ρ_calcd_*=1.333 Mg/m^3^, *μ*=0.487 mm^−1^, F(000)=664, 17161 reflections, −11≤h≤11, −24≤k≤23, −12≤l≤12, 2.264<*θ*<26.777°, completeness 99.8 %, 3511 independent reflections, 2900 reflections observed with [I>2*σ*(I)], 197 parameters, 0 restraints, *R* indices (all data) *R*1=0. 0478, *wR*2=0.0634, final *R* indices [I>2*σ*(I)] *R*1=0.0334, *wR*2=0.0592, largest difference peak and hole 0.450 and −0.485 eA^−3^, GooF=1.032.

**Crystal data for [W(CO)_5_(NHSi)] (5)**: C_31_H_36_N_2_WO_5_Si, M_r_=728.56, *T*=100(2) K, *λ*=0.71073 Å, yellow block, 0.079×0.182×0.192 mm^3^, monoclinic space group *P*2(1)/*m*, *a=*9.1098(10) Å, *b=*19.486(2) Å, *c=*9.5658(11) Å, *β*=110.252(4)°, *V*=1593.1(3) Å^3^, *Z*=1, *ρ_calcd_*=1.519 Mg/m^3^, *μ*=3.702 mm^−1^, F(000)=728, 23543 reflections, −11≤h≤11, −24≤k≤24, −12≤l≤12, 2.662<*θ*<26.820°, completeness 99.3 %, 3500 independent reflections, 3167 reflections observed with [I>2*σ*(I)], 265 parameters, 0 restraints, *R* indices (all data) *R*1=0. 0338, *wR*2=0.0641, final *R* indices [I>2*σ*(I)] *R*1=0.0281, *wR*2=0.0618, largest difference peak and hole 2.361 and −0.812 eA^−3^, GooF=1.048.

**Crystal data for [W(CO)_5_(*i*Pr_2_Im)] (6)**: C_14_H_16_N_2_WO_5_, M_r_=476.13, *T*=100(2) K, *λ*=0.71073 Å, yellow block, 0.062×0.346×0.561 mm^3^, orthorhombic space group *Pbca*, *a=*13.6965(14) Å, *b=*12.8373(13) Å, *c=*18.3680(18) Å, *V*=3229.6(6) Å^3^, *Z*=8, *ρ_calcd_*=1.959 Mg/m^3^, *μ*=7.177 mm^−1^, F(000)=1824, 22302 reflections, −17≤h≤17, −16≤k≤15, −20≤l≤23, 2.441 < *θ*<26.809°, completeness 99.7 %, 3448 independent reflections, 2951 reflections observed with [I>2*σ*(I)], 203 parameters, 0 restraints, *R* indices (all data) *R*1=0. 0336, *wR*2=0.0994, final *R* indices [I>2*σ*(I)] *R*1=0.0268, *wR*2=0.0874, largest difference peak and hole 0.911 and −02.142 eA^−3^, GooF=0.805.

**Crystal data for [W(CO)_5_(*i*Pr_2_Im^Me^)] (7)**: C_16_H_20_N_2_WO_5_, M_r_=504.19, *T*=100(2) K, *λ*=0.71073 Å, yellow block, 0.164×0.243×0.491 mm^3^, monoclinic space group *P*2_1_/*n*, *a=*9.4325(8) Å, *b=*12.7850(10) Å, *c=*14.6688(12) Å, *β*=94.558(2)°, *V*=1763.4(2) Å^3^, *Z*=4, *ρ_calcd_*=1.899 Mg/m^3^, *μ*=6.578 mm^−1^, F(000)=976, 28450 reflections, −11≤h≤11, −16≤k≤16, −18≤l≤18, 2.116 < *θ*<26.857°, completeness 99.9 %, 3789 independent reflections, 3666 reflections observed with [I>2*σ*(I)], 223 parameters, 0 restraints, *R* indices (all data) *R*1=0. 0134, *wR*2=0.0337, final *R* indices [I>2*σ*(I)] *R*1=0.0129, *wR*2=0.0334, largest difference peak and hole 0.681 and −0.629 eA^−3^, GooF=1.110.

**Crystal data for [Mn(CO)_3_(Dipp_2_NHSi)_2_(Br)] (9)**: C_55_H_72_N_4_MnO_3_Si_2_Br_1_, M_r_=1028.22, *T*=100(2) K, *λ*=0.71073 Å, orange block, 0.16×0.405×0.449 mm^3^, orthorhombic space group *P*2_1_2_1_2_1_, *a*=12.1025(3) Å, *b*=19.0741(4) Å, *c*=23.6608(6) Å, *V*=5462.0(2) Å^3^, *Z*=4, *ρ*_calcd_=1.250 Mg/m^3^, *μ*=1.061 mm^−1^, F(000)=2168, 62019 reflections, −16≤h≤15, −25≤k≤25, −31≤l≤31, 1.371<θ<28.357°, completeness 1.82/1.00, 13621 independent reflections, 11762 reflections observed with [I>2σ(I)], 613 parameters, 0 restraints, *R* indices (all data) *R*1=0. 0445, *wR*2=0.0819, final *R* indices [I>2σ(I)] *R*1=0.0348, *wR*2=0.0790, largest difference peak and hole 1.547 and −0.561 eA^−3^, GooF=1.006.

**Crystal data for [(*μ*^5^‐C_5_H_5_)Fe(CO)_2_(Dipp_2_NHSiI)] (10)**: C_33_H_41_N_2_FeIO_2_Si, M_r_=708.54, *T*=100(2) K, *λ*=0.71073 Å, orange block, 0.175×0.211×0.242 mm^3^, monoclinic space group *P*2(1)/*n*, *a=*17.6210(11) Å, *b=*10.6925(6) Å, *c=*18.1746(11) Å, *β*=110.295(2)°, *V*=3211.7(3) Å^3^, *Z*=4, *ρ_calcd_*=1.465 Mg/m^3^, *μ*=1.499 mm^−1^, F(000)=1448, 56480 reflections, −23≤h≤23, −14≤k≤14, −24≤l≤24, 1.992 < *θ*<28.360°, completeness 99.7 %, 8020 independent reflections, 6976 reflections observed with [I>2*σ*(I)], 369 parameters, 0 restraints, *R* indices (all data) *R*1=0. 0521, *wR*2=0.1158, final *R* indices [I>2*σ*(I)] *R*1=0.0438, *wR*2=0.1117, largest difference peak and hole 2.891 and −0.896 eA^−3^, GooF=1.094.

Computational details: Calculations on the NHCs, NHSis and the complex [{Ni(CO)_2_(*μ*‐NHSi)}_2_] were carried out using the TURBOMOLE V7.2 2017 program suite, a development of the University of Karlsruhe and the Forschungszentrum Karlsruhe GmbH, 1989–2007, TURBOMOLE GmbH, since 2007; available from http://www.turbomole.com.^53^ Geometry optimizations were performed using (RI‐)DFT calculations^54^ on a m4 grid employing the BP86^55^ functional and a def2‐SV(P)^56^ basis set for all atoms or employing the B3LYP^57^ functional or a def2‐TZVPP^56^ basis set for selected or all atoms. Vibrational frequencies were calculated at the same level with the AOFORCE^58^ module and all structures represented true minima without imaginary frequencies.

All calculations for the energy decomposition analysis were carried out using the Amsterdam Density Functional (ADF) program.^59^ The numerical integration was performed using a procedure developed by Becke *et al*.^60^ The molecular orbitals (MOs) were expanded in a large uncontracted set of Slater type orbitals (STOs) containing diffuse functions: a triple‐ζ quality basis set was used for all atoms,^61^ augmented with two sets of polarization functions for H (2p, 3d), C, N, O, Si, (3d, 4f), Ni (4p, 4f) and W (6p, 5f). An auxiliary set of s, p, d, f and g STOs was used to fit the molecular density and to represent the Coulomb and exchange potentials accurately in each self‐consistent field (SCF) cycle. All electrons were included in the variational treatment (no frozen‐core approximation was used). The generalized gradient approximation (GGA) at the BLYP level was used where exchange is described by Slater Xα potential,^62^ with non‐local corrections due to Becke^55^ added self‐consistently, and where correlation was treated by using the Lee–Yang–Parr gradient‐corrected functional.^58^, ^63^ Relativistic effects were included with the scalar‐zero‐order‐regular‐approximation (ZORA).^64^ In addition, the D3(BJ) dispersion correction was used.^65^ This level of theory is denoted as TZ2P/BLYP/ZORA/D3(BJ) throughout the text. Energy minima have been verified by vibrational analysis.^66^ Voronoi *deformation* density (VDD) charges^67^ were calculated for the optimized gas‐phase structures at the same level of theory.

The interaction energy (Δ*E*_int_) between Ni (d^10^s^0^) and the NHC^Me^/NHSi^Me^ fragments can be decomposed into the following terms [Eq. (1)]:

(1) $$\Delta E{}_{\mathrm{int}}=\Delta E{}_{\mathrm{Pauli}}+\Delta V{}_{\mathrm{elstat}}+\Delta E{}_{\mathrm{disp}}+\Delta E{}_{\mathrm{oi}}$$

This energy decomposition analysis (EDA)^68^ quantifies the Pauli‐repulsive orbital interactions (Δ*E*_Pauli_) between same‐spin electrons, the electrostatic interaction (Δ*V*_elstat_), the interaction due to dispersion forces (Δ*E*_disp_) and orbital interactions (Δ*E*_oi_), that emerge from charge transfer (interaction between occupied orbitals on one fragment with unoccupied orbitals on the other fragment, including donor‐acceptor interactions) and polarization (empty‐occupied orbital mixing on one fragment due to the presence of the other fragment). It can be further divided into contributions from each irreducible representation *Γ* of the interacting system (Equation (2)).

(2) $$\Delta E{}_{\mathrm{oi}}\left(\zeta \right)=\sum {}_{\Gamma }\;\Delta E{}_{\mathrm{oi}}{}^{\Gamma }\;\left(\zeta \right)$$

The percentage AO contribution to MOs is based on gross Mulliken contributions.^68^, ^69^ The Tolman electronic parameter (TEP) was calculated by simulating the IR‐spectra of [Ni(CO)_3_(NHC^Me^)] and [Ni(CO)_3_(NHSi^Me^)], respectively. The frequencies obtained were corrected by using an empirical formula [Eq. (3)]:

(3) TEP=0.8609x+376.28

## Supporting information

As a service to our authors and readers, this journal provides supporting information supplied by the authors. Such materials are peer reviewed and may be re‐organized for online delivery, but are not copy‐edited or typeset. Technical support issues arising from supporting information (other than missing files) should be addressed to the authors.

Supplementary
