# Supplementary material for: N‐Heterocyclic Silylenes as Ligands in Transition Metal Carbonyl Chemistry: Nature of Their Bonding and Supposed Innocence
Source: Chemistry. 2020 Jul 27;26(49):11276–92. doi: 10.1002/chem.202001062 (PMC7497151; doi:10.1002/chem.202001062)
Supplement: Supplementary file 1 — Supplementary [file CHEM-26-11276-s001.pdf]

Chemistry–A European Journal

Supporting Information

N-Heterocyclic Silylenes as Ligands in Transition Metal Carbonyl Chemistry: Nature of Their Bonding and Supposed Innocence

Mirjam J. Krahfuß,[a] Jörn Nitsch,[a] F. Matthias Bickelhaupt,[b, c] Todd B. Marder,[a, d] and Udo Radius*[a]

Table of Contents

1. Additional Tables and Figures 1

1. Solution NMR Spectra of 2 – 10 3

2. IR Spectra of 2 – 10 18

3. Computational details 23

1. Additional Tables and Figures

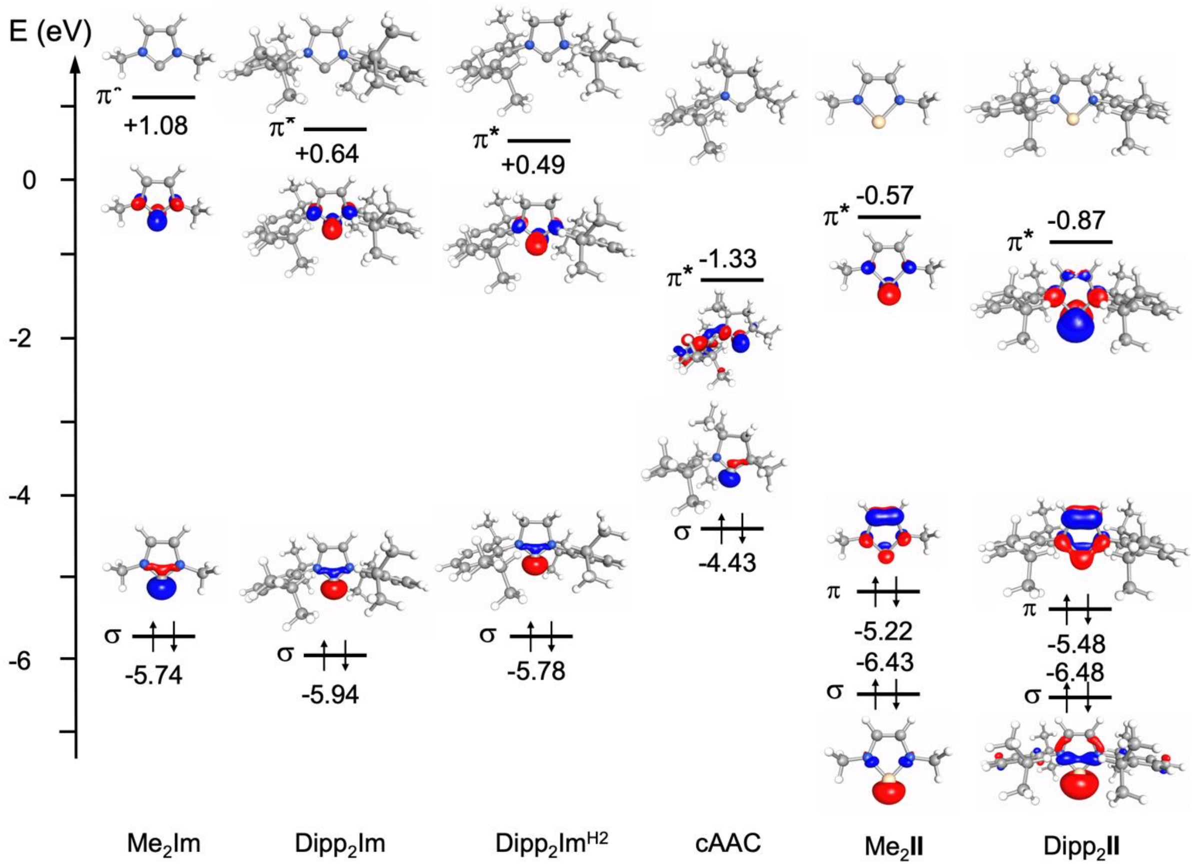

Figure S1: Important frontier orbitals of the NHSi **Dipp₂II** and NHSi^{Me} with respect to those of commonly used NHC ligands Me_2Im, $Dipp_2Im$, $Dipp_2Im^{H2}$ and cAAC^{Me}. Energies were calculated at the DFT/def2-TZVPP/B3-LYP-D3(BJ) level of theory, orbital plots are drawn at the 0.1 isosurface.

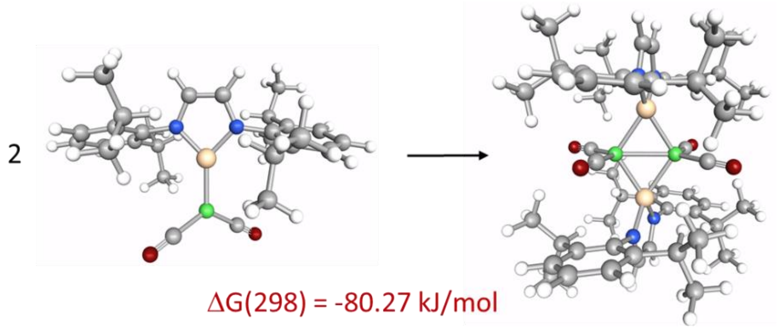

Figure S2. DFT calculations (TURBOMOLE/def2-SV(P)/BP86) on the dimerization of [Ni(CO)$_2$(NHSi)] **2a** to give the dimer [{Ni(CO)$_2$(μ-NHSi)}$_2$] **2**.

Table S1. Bond lengths of W–C in [W(CO)$_5$(L)] (L = Dipp$_2$NHSi **5**, *i*Pr$_2$Im **6**, *i*Pr$_2$Im^{Me} **7**) complexes.

**Bond lengths [Å]**	**[W(CO)$_5$(Dipp$_2$NHSi)] 5**	**[W(CO)$_5$(*i*Pr$_2$Im)] 6**	**[W(CO)$_5$(*i*Pr$_2$Im^{Me})] 7**
**W1─Si1 / W1─C6**	2.4576(14)	2.272(4)	2.2930(18)
**W1─C1**	2.010(5)	1.999(4)	1.9883(19)
**W1─C2**	2.051(4)	2.033(4)	2.0367(19)
**W1─C3**	2.038(5)	2.034(4)	2.033(2)
**W1─C4**	2.021(6)	2.049(4)	2.0414(19)
**W1─C5**	-	2.047(4)	2.047(2)
**C1─O1**	1.147(6)	1.155(5)	1.154(2)
**C2─O2**	1.141(5)	1.149(5)	1.141(2)
**C3─O3**	1.145(6)	1.154(4)	1.144(2)
**C4─O4**	1.163(6)	1.138(5)	1.144(2)
**C5─O5**	-	1.144(4)	1.135(2)

2. Solution NMR Spectra of 2 – 10

NMR Spectra of [{Ni(CO)$_2$(μ-Dipp$_2$NHSi)}$_2$] (2)

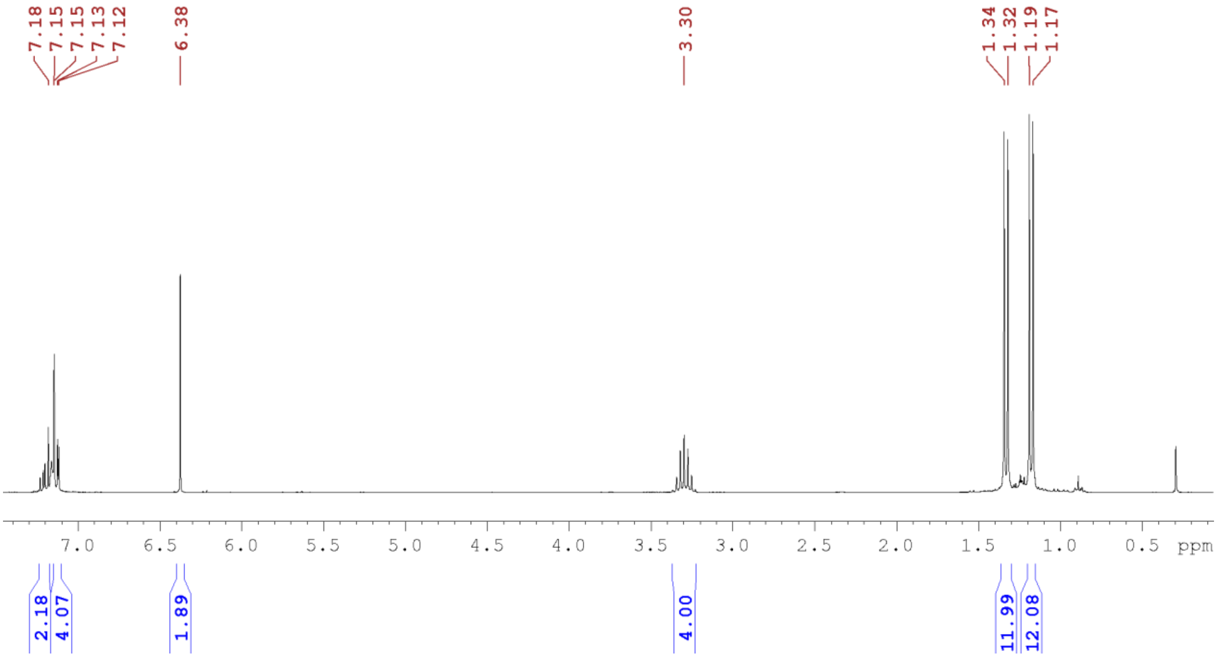

Figure S3. ^{1}H NMR spectrum (300.1 Hz) of [{Ni(CO)$_2$(μ-Dipp$_2$NHSi)}$_2$] in C_6D_6.

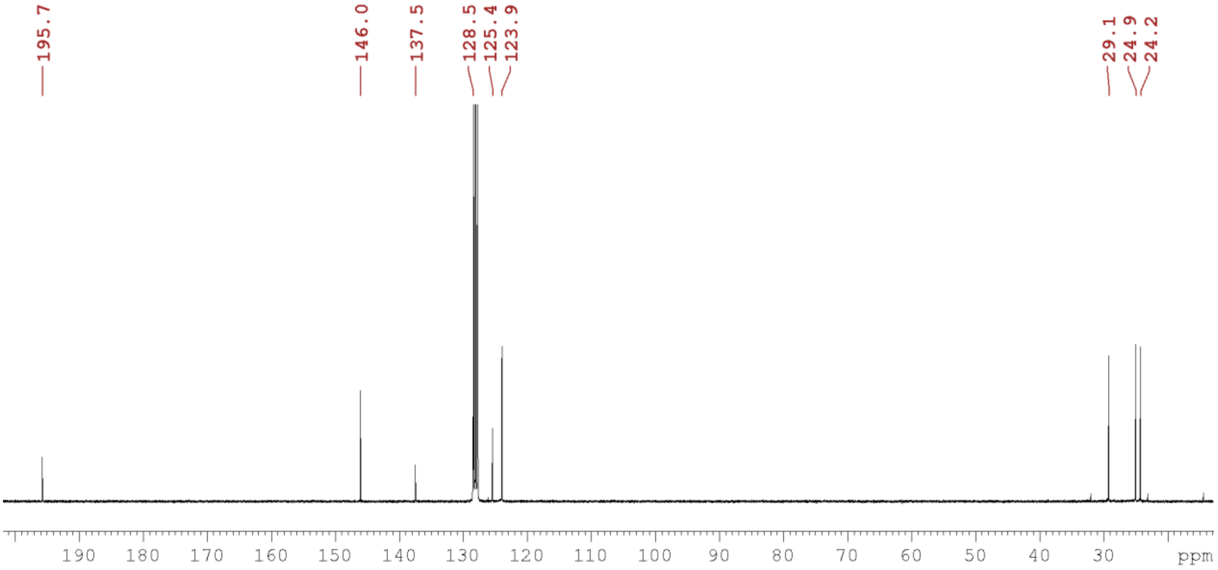

Figure S4. ^{13}C{^{1}H} NMR spectrum (75.5 Hz) of [{Ni(CO)$_2$(μ-Dipp$_2$NHSi)}$_2$] in C_6D_6.

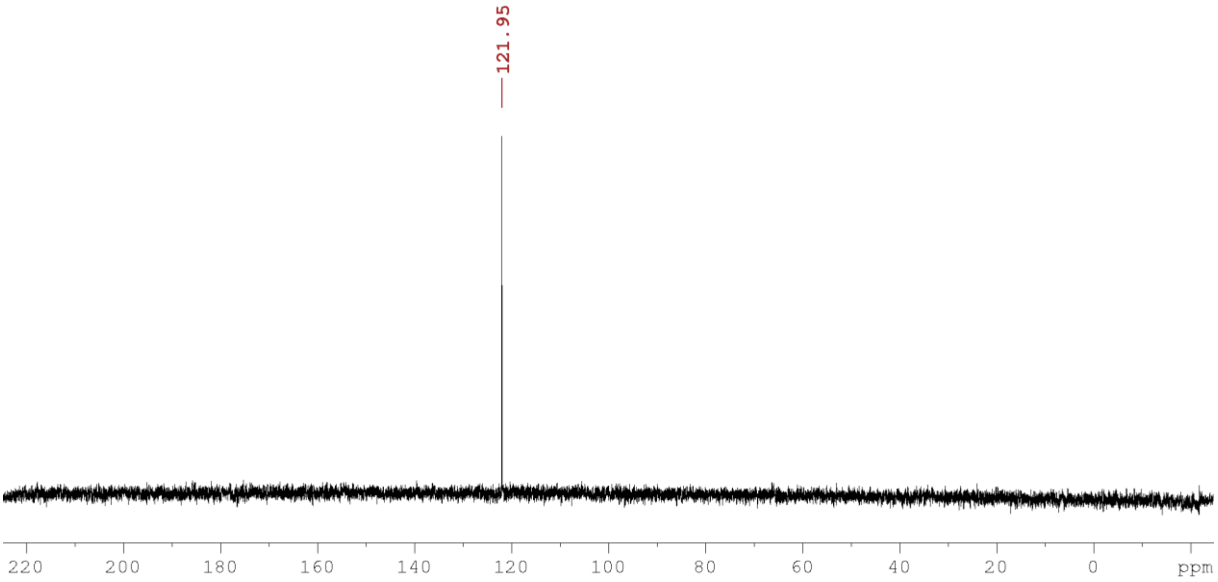

Figure S5. ^{29}Si{^{1}H} NMR spectrum (59.6 Hz) of [{Ni(CO)$_2$(*μ*-Dipp$_2$NHSi)}$_2$] in C_6D_6.

NMR Spectra of [Cr(CO)$_5$(Dipp$_2$NHSi)] (3)

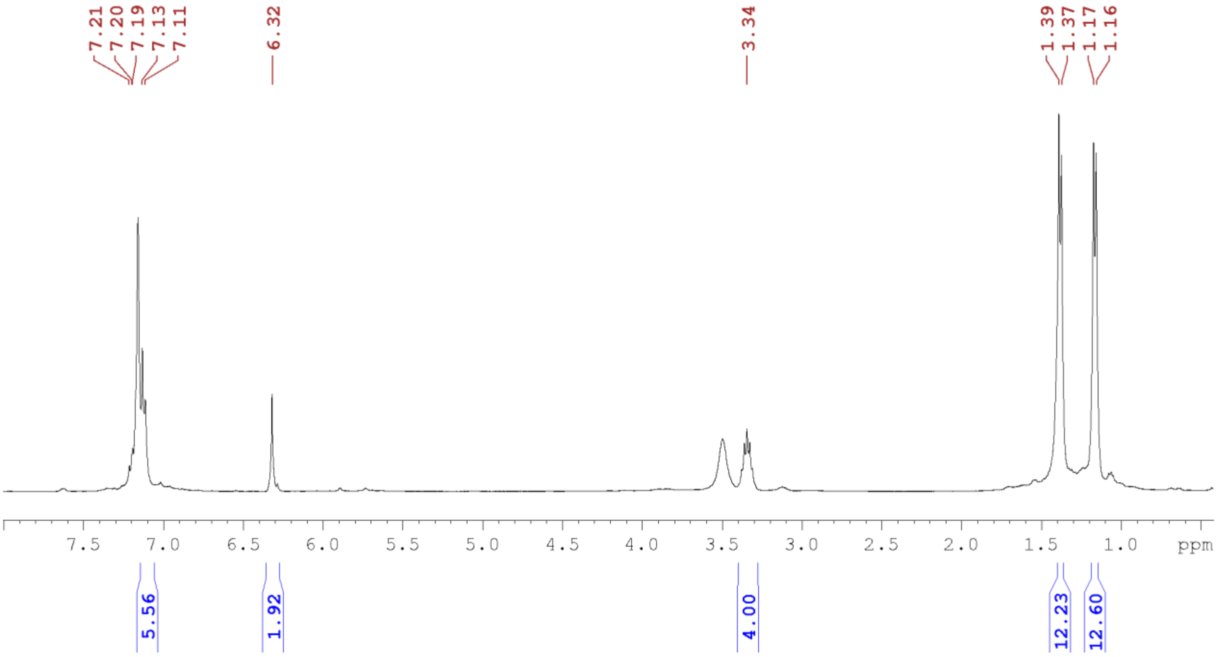

Figure S6. ^{1}H NMR spectrum (400.3 Hz) of [Cr(CO)$_5$(Dipp$_2$NHSi)] in C_6D_6.

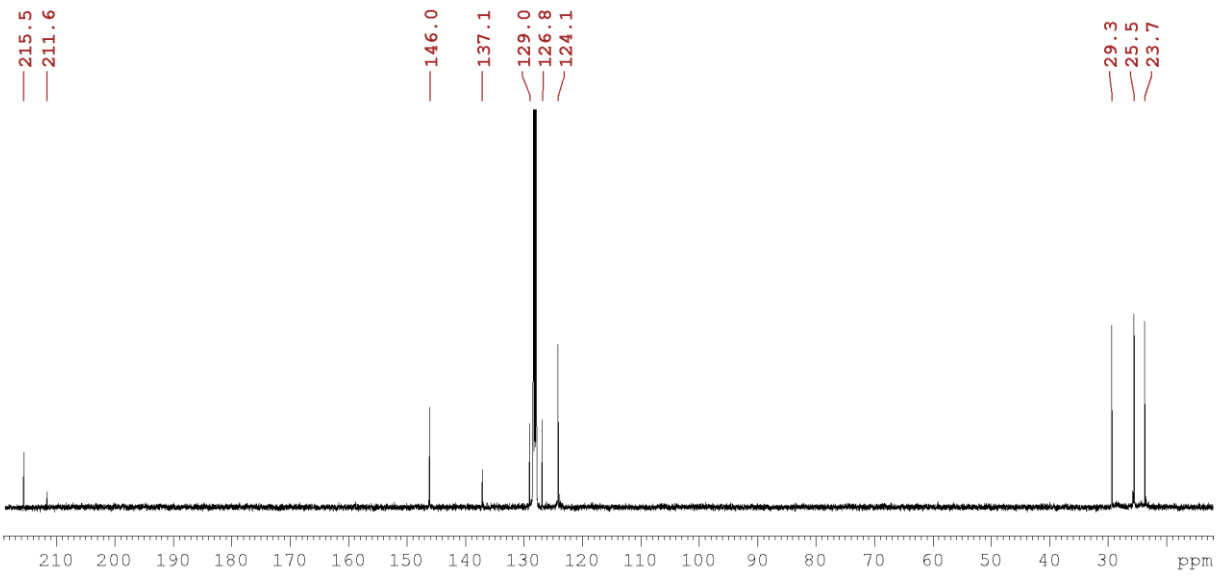

Figure S7. $^{13}C\{^1H\}$ NMR spectrum (100.7 Hz) of $[Cr(CO)_5(Dipp_2NHSi)]$ in C_6D_6.

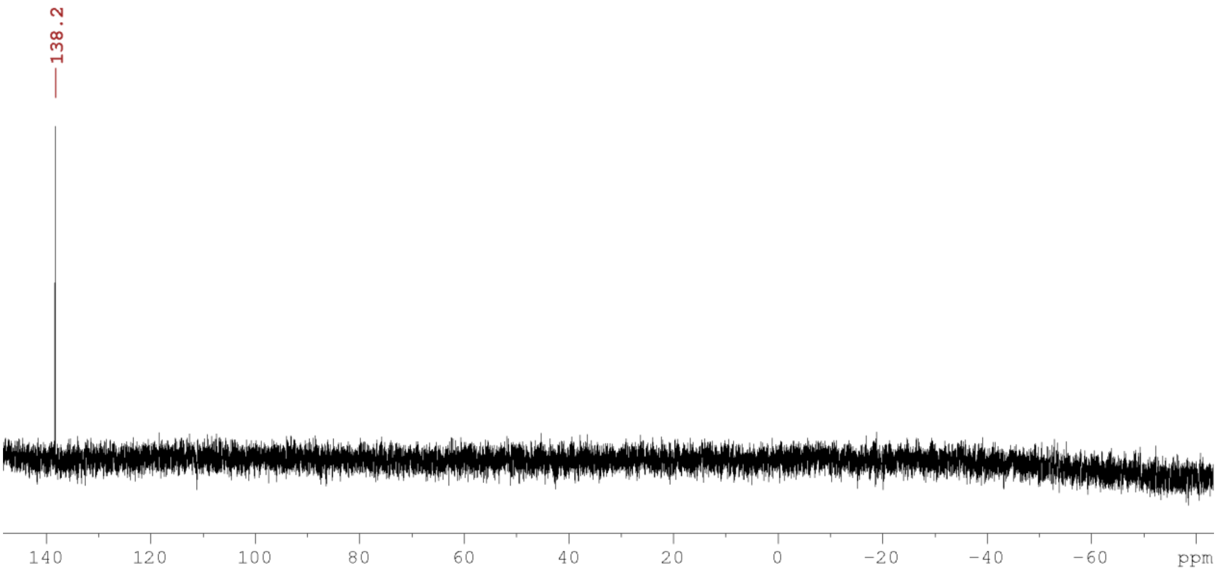

Figure S8. $^{29}Si\{^1H\}$ NMR spectrum (79.5 Hz) of $[Cr(CO)_5(Dipp_2NHSi)]$ in C_6D_6.

NMR Spectra of [Mo(CO)$_5$(Dipp$_2$NHSi)] (4)

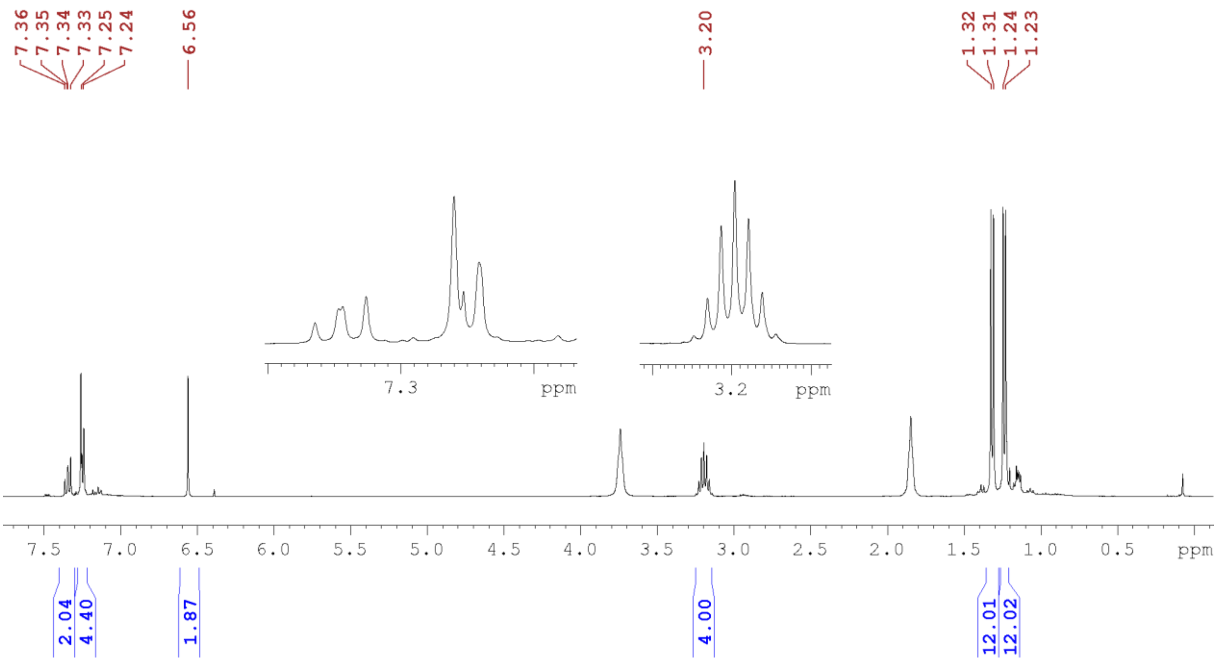

Figure S9. ^{1}H NMR spectrum (400.3 Hz) of [Mo(CO)$_5$(Dipp$_2$NHSi)] in $CDCl_3$.

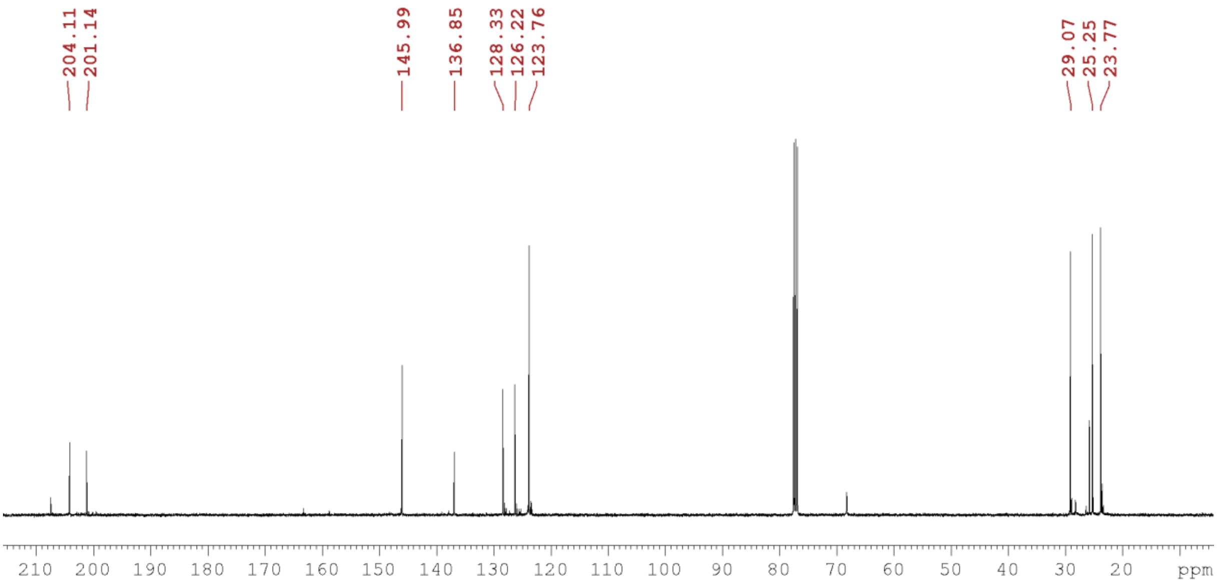

Figure S10. ^{13}C{^{1}H} NMR spectrum (100.7 Hz) of [Mo(CO)$_5$(Dipp$_2$NHSi)] in $CDCl_3$.

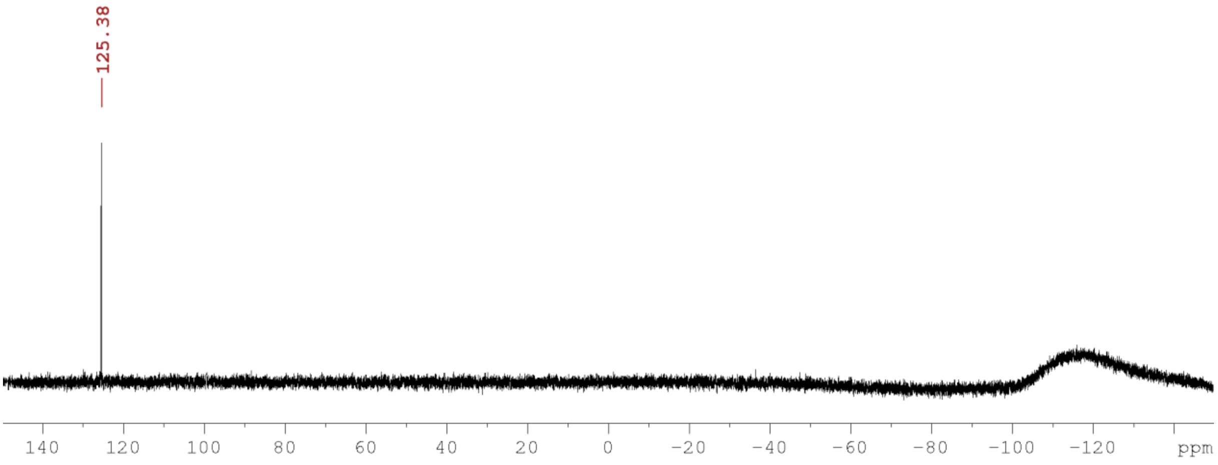

Figure S11. $^{29}Si\{^1H\}$ NMR spectrum (79.5 Hz) of [Mo(CO)$_5$(Dipp$_2$NHSi)] in $CDCl_3$.

NMR Spectra of [W(CO)$_5$(Dipp$_2$NHSi)] (5)

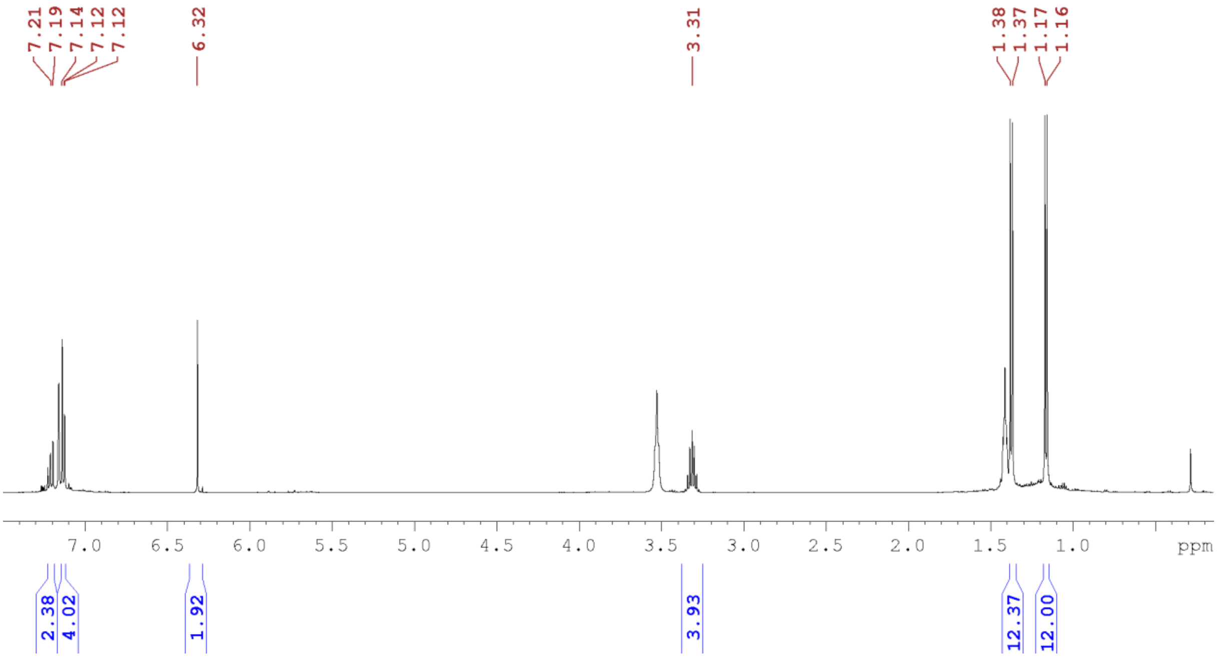

Figure S12. ^1H NMR spectrum (500.1 Hz) of [W(CO)$_5$(Dipp$_2$NHSi)] in C_6D_6.

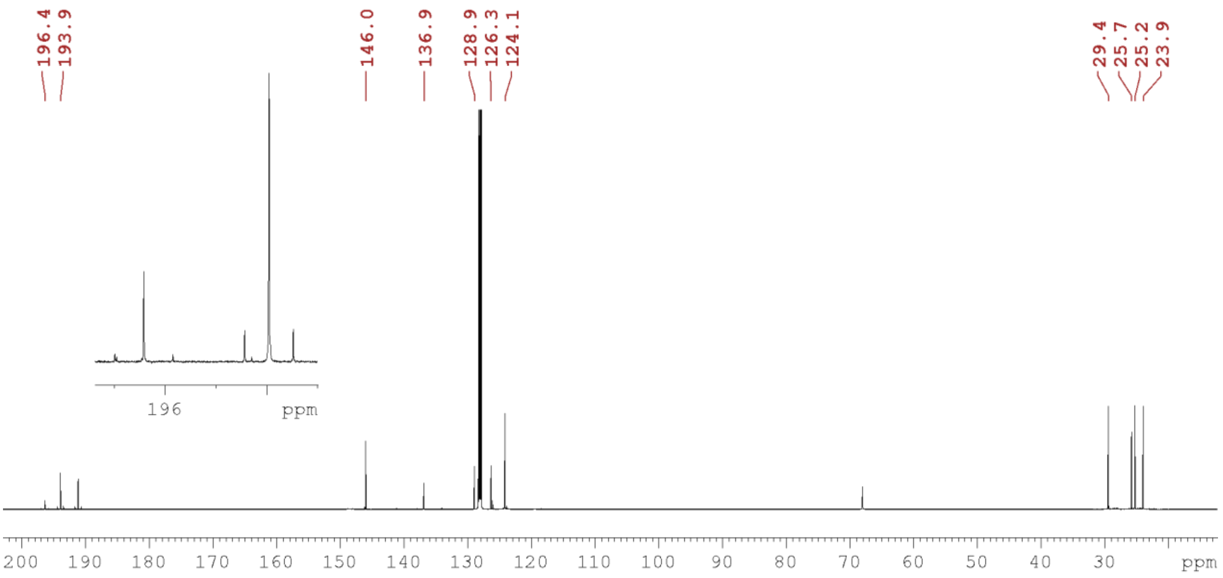

Figure S13. $^{13}C\{^1H\}$ NMR spectrum (125.8) of $[W(CO)_5(Dipp_2NHSi)]$ in C_6D_6.

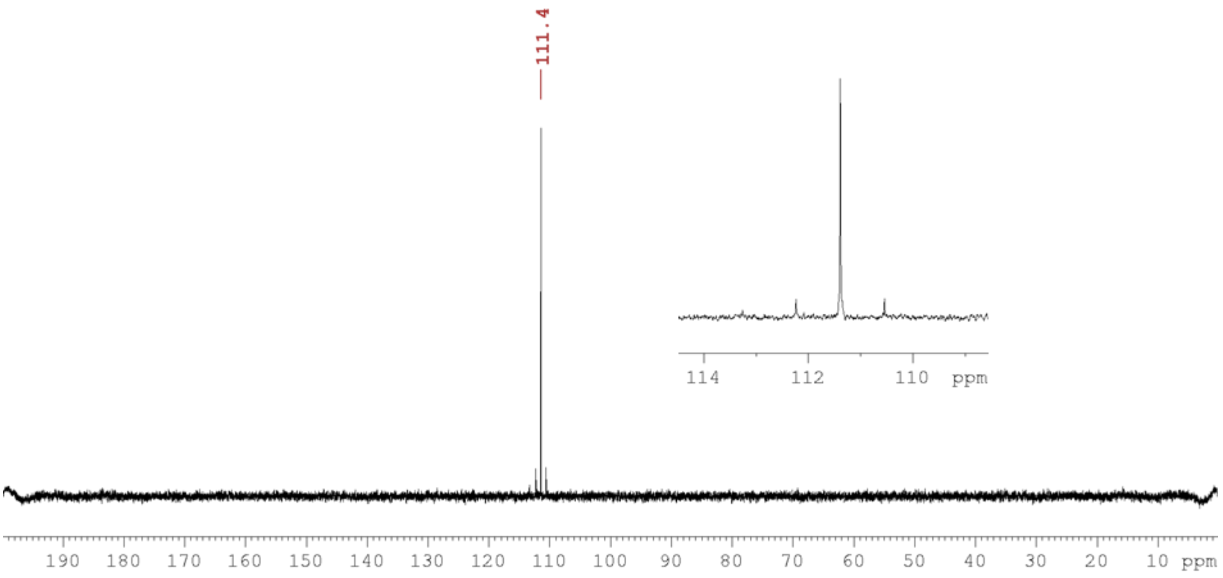

Figure S14. $^{29}Si\{^1H\}$ NMR spectrum (99.4 Hz) of $[W(CO)_5(Dipp_2NHSi)]$ in C_6D_6.

NMR Spectra of [W(CO)$_5$(*i*Pr$_2$Im)] (6)

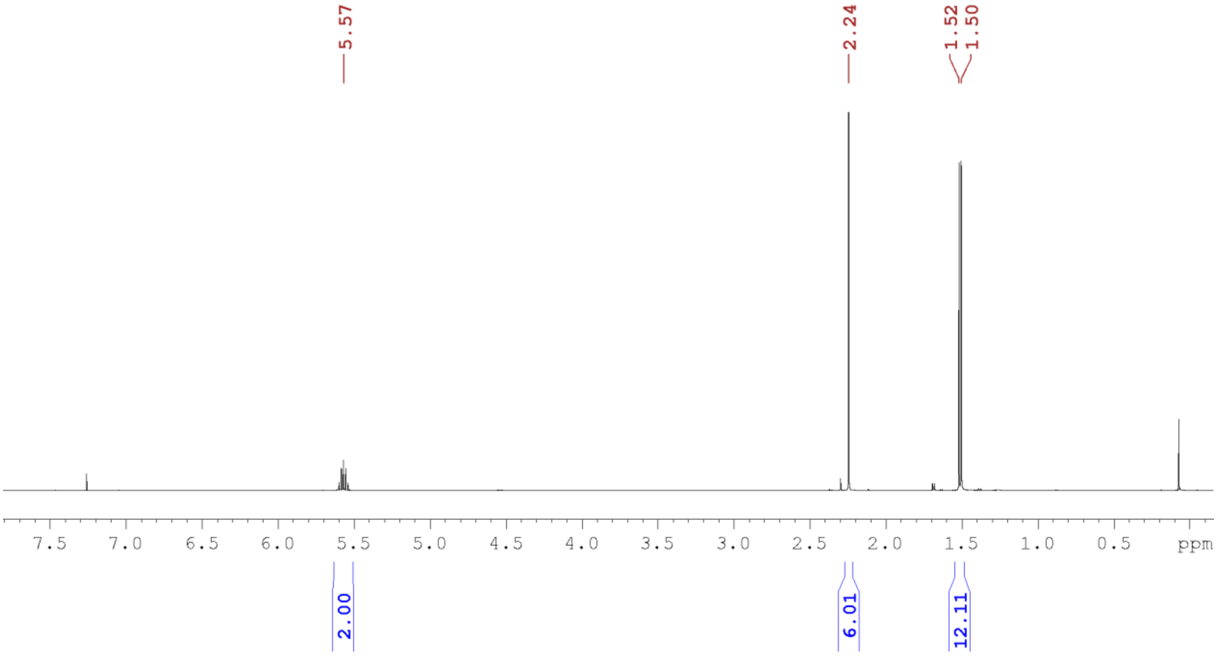

Figure S15. ^{1}H NMR spectrum (300.1 Hz) of [W(CO)$_5$(*i*Pr$_2$Im)] in d^8-THF.

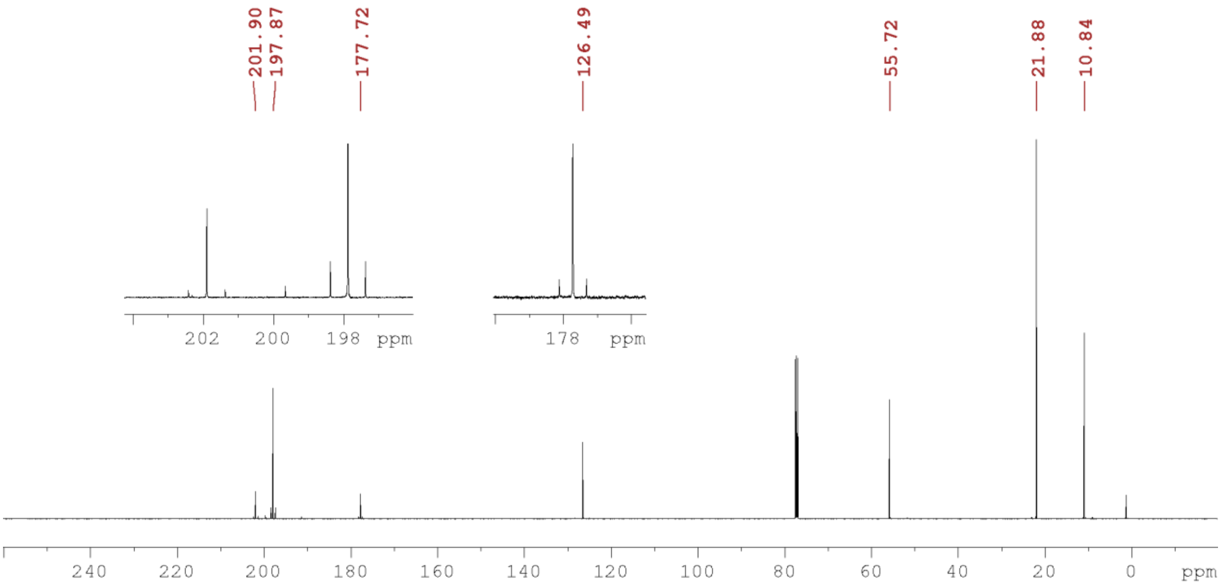

Figure S16. ^{13}C{^{1}H} NMR spectrum (75.5 Hz) of [W(CO)$_5$(*i*Pr$_2$Im)] in d^8-THF.

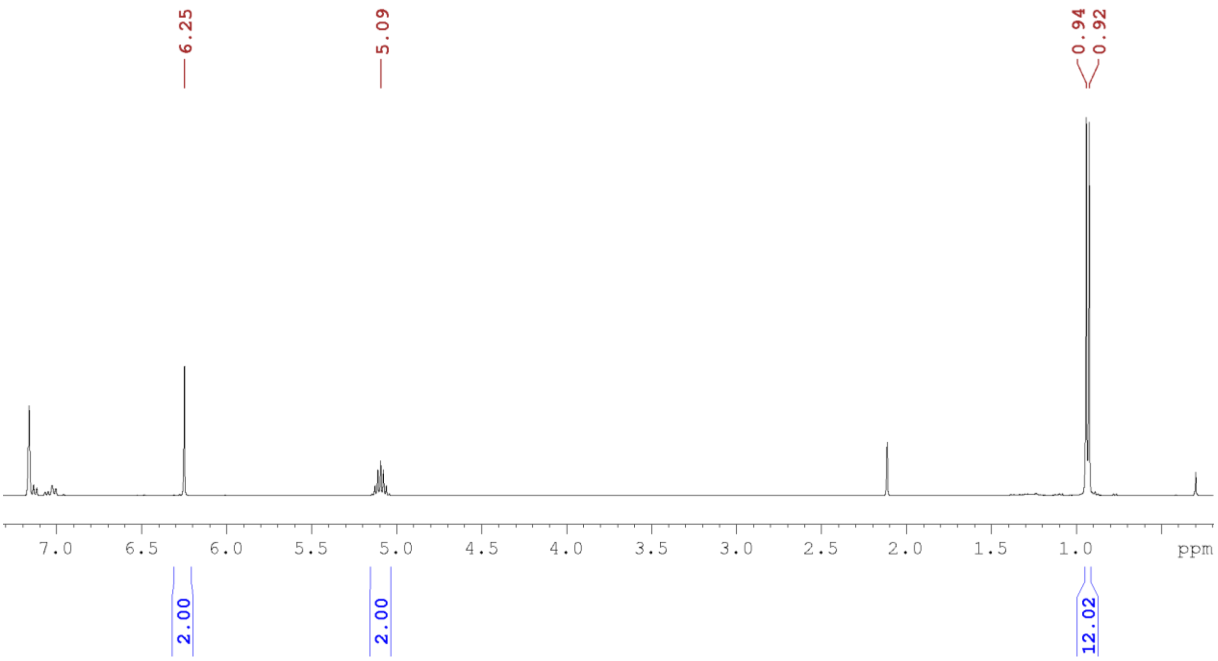

Figure S17. ^{1}H NMR spectrum (400.3 Hz) of [W(CO)$_5$(*i*Pr$_2$Im)] in C_6D_6.

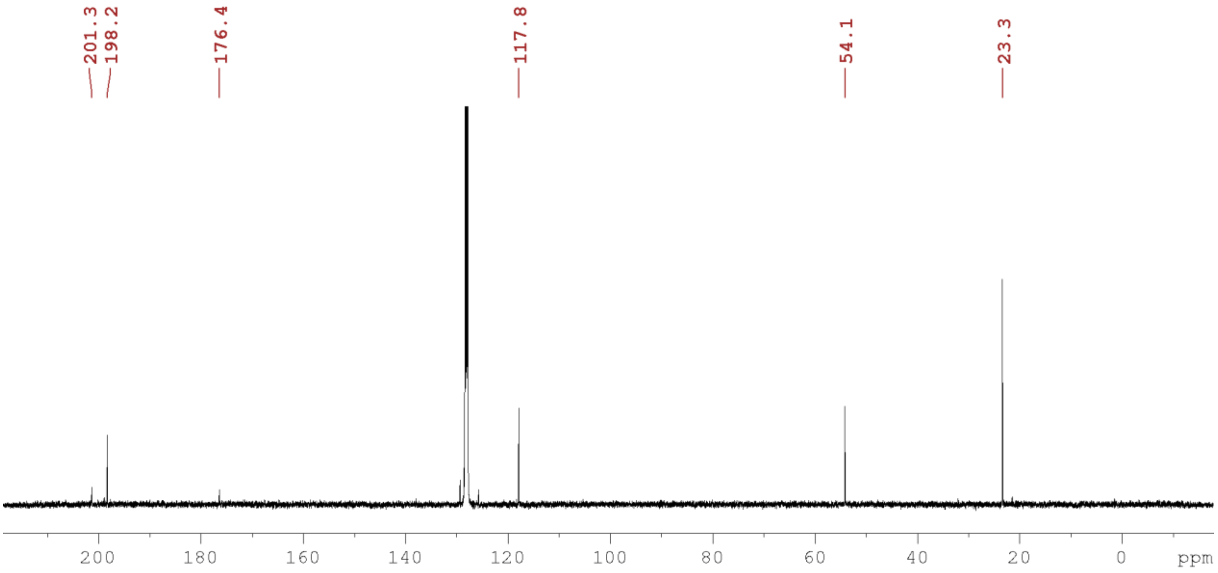

Figure S18. ^{13}C{^{1}H} NMR spectrum (100.7 Hz) of [W(CO)$_5$(*i*Pr$_2$Im)] in C_6D_6.

NMR Spectra of [W(CO)$_5$(*i*Pr$_2$Im^{Me})] (7)

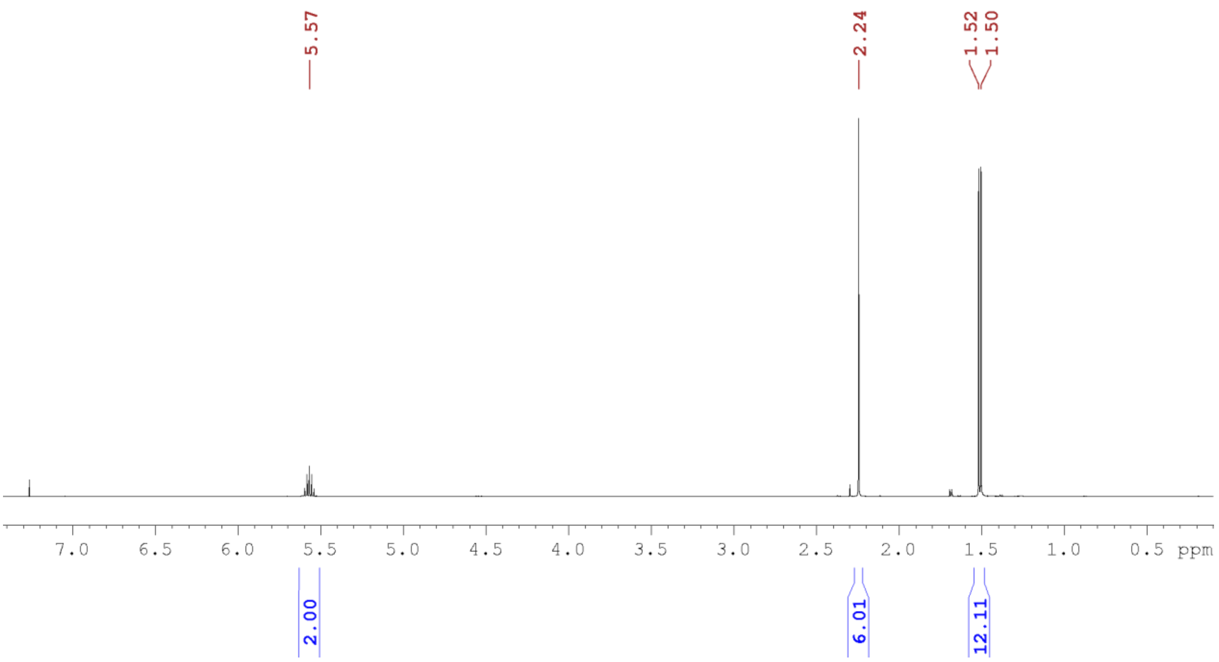

Figure S19. ^{1}H NMR spectrum (500.1 Hz) of [W(CO)$_5$(*i*Pr$_2$Im^{Me})] in CDCl$_3$.

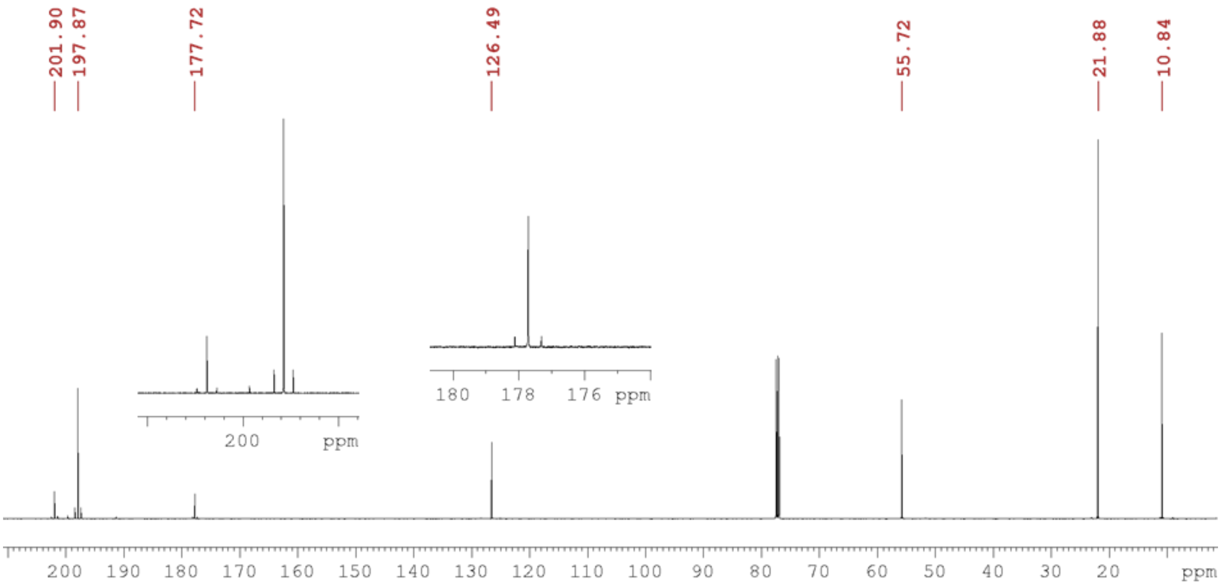

Figure S20. ^{13}C{^{1}H} NMR spectrum (125.8 Hz) of [W(CO)$_5$(*i*Pr$_2$Im^{Me})] in CDCl$_3$.

NMR Spectra of [W(CO)$_5$(Me$_2$Im^{Me})] (8)

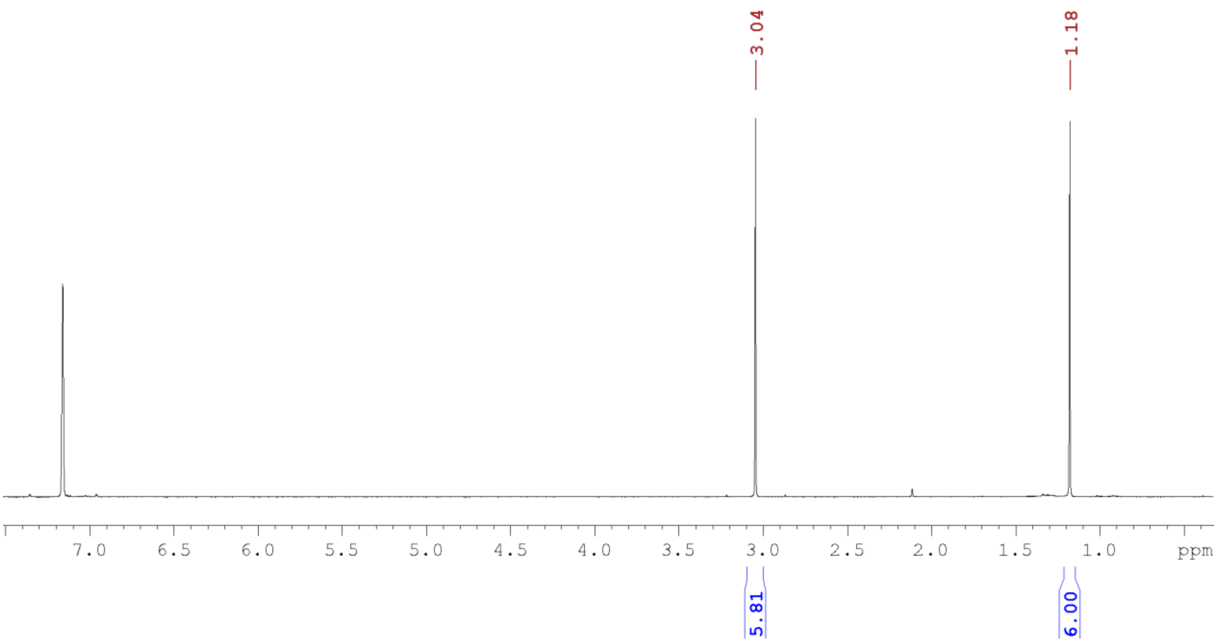

Figure S21. ^{1}H NMR spectrum (500.1 Hz) of [W(CO)$_5$(Me$_2$Im^{Me})] in C$_6$D$_6$.

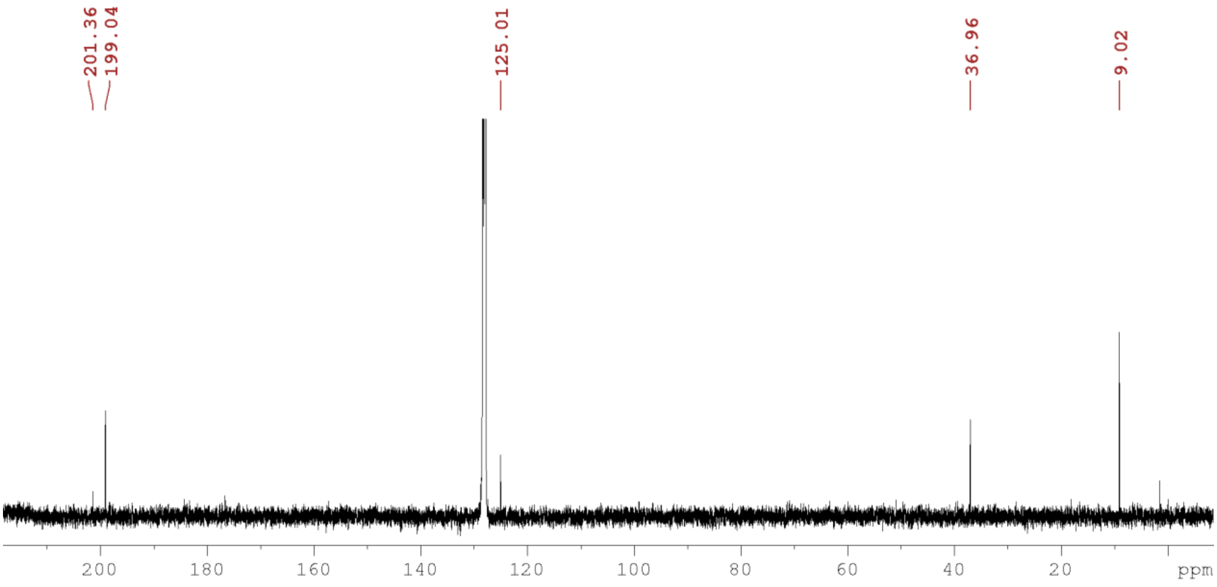

Figure S22. ^{13}C{^{1}H} NMR spectrum (125.8 Hz) of [W(CO)$_5$(Me$_2$Im^{Me})] in C$_6$D$_6$.

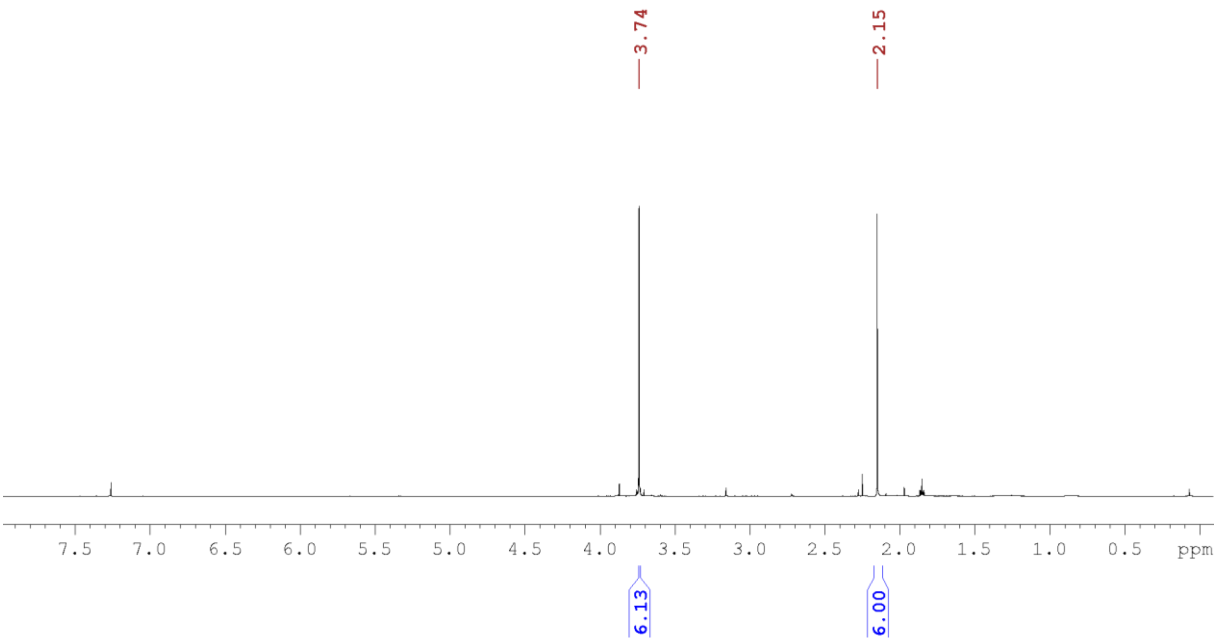

Figure S23. ^{1}H NMR spectrum (500.1 Hz) of [W(CO)$_5$(Me$_2$Im^{Me})] in $CDCl_3$.

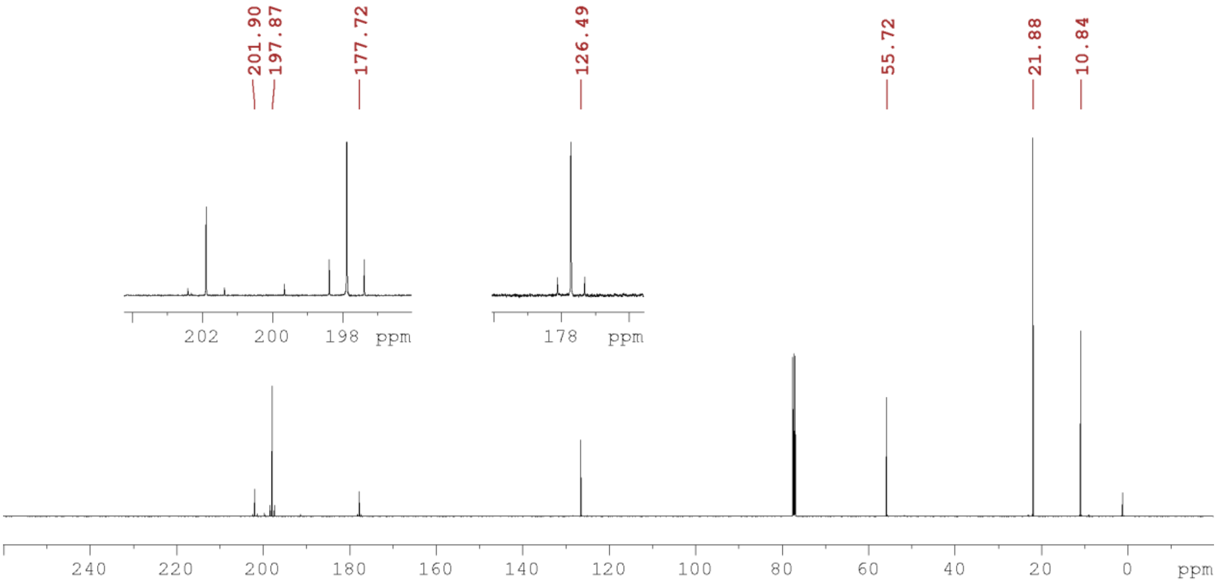

Figure S24. $^{13}C\{^{1}H\}$ NMR spectrum (125.8 Hz) of [W(CO)$_5$(Me$_2$Im^{Me})] in $CDCl_3$.

NMR Spectra of [Mn(CO)$_3$(Dipp$_2$NHSi)$_2$(Br)] (9)

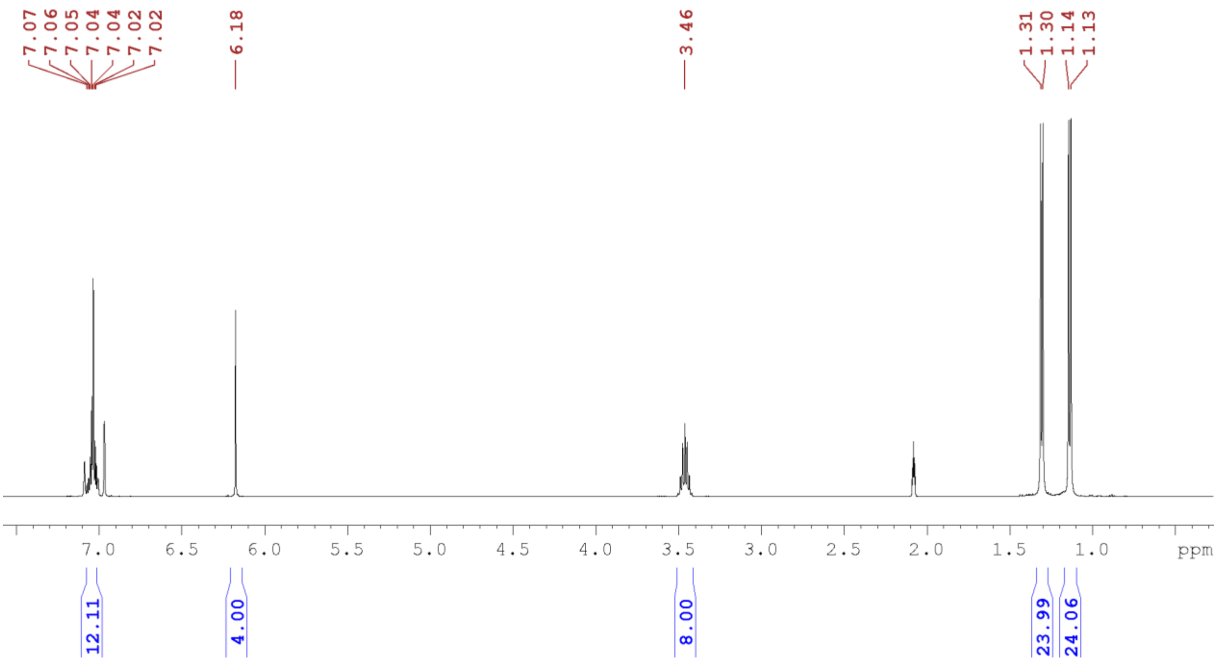

Figure S25. ^{1}H NMR spectrum (500.1 Hz) of [Mn(CO)$_3$(Dipp$_2$NHSi)$_2$(Br)] in d^8-toluene at 25 °C.

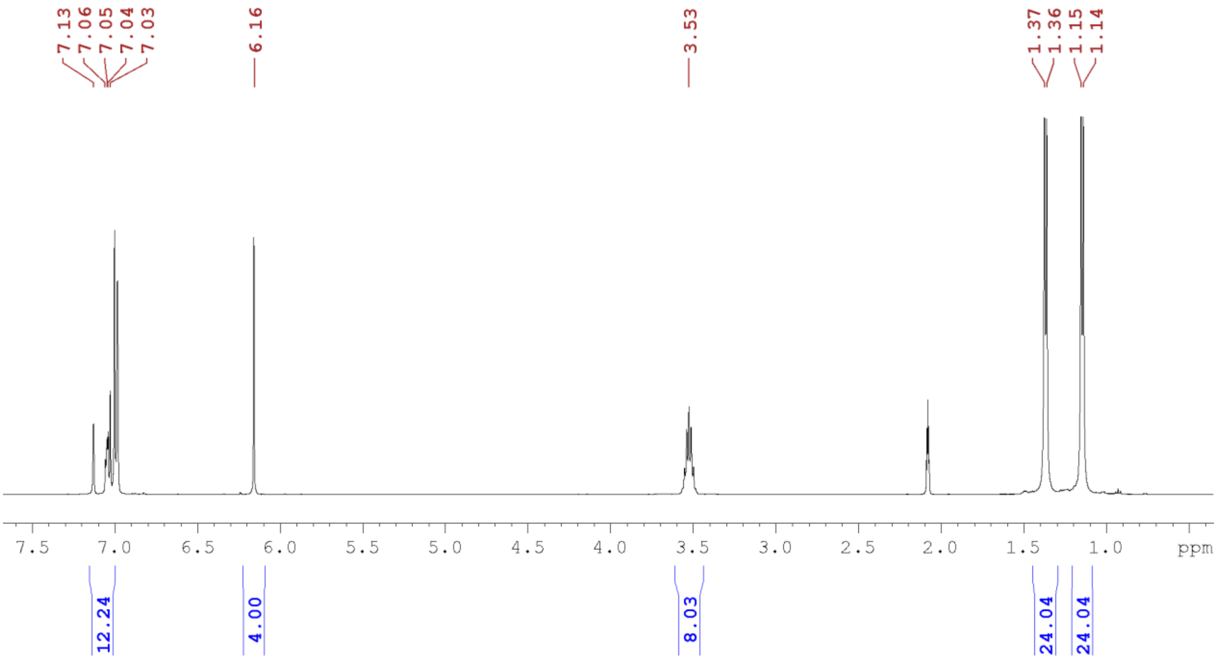

Figure S26. ^{1}H NMR spectrum (500.1 Hz) of [Mn(CO)$_3$(Dipp$_2$NHSi)$_2$(Br)] in d^8-toluene at -40 °C.

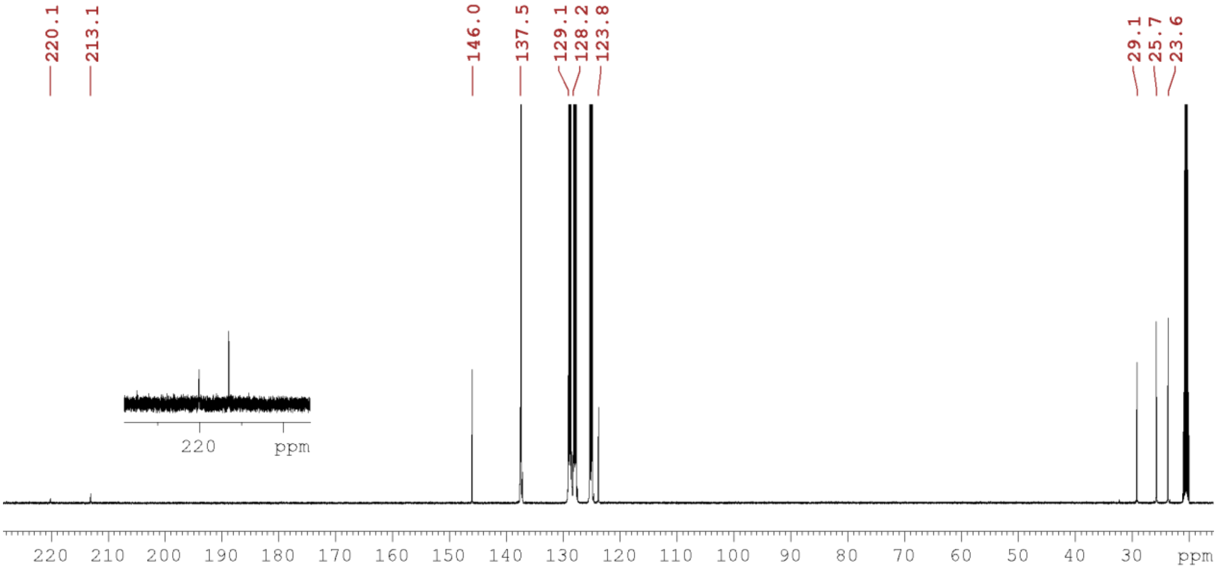

Figure S27. $^{13}C\{^{1}H\}$ NMR spectrum (125.8 Hz) of [Mn(CO)$_3$(Dipp$_2$NHSi)$_2$(Br)] in d^8-toluene at -40 °C.

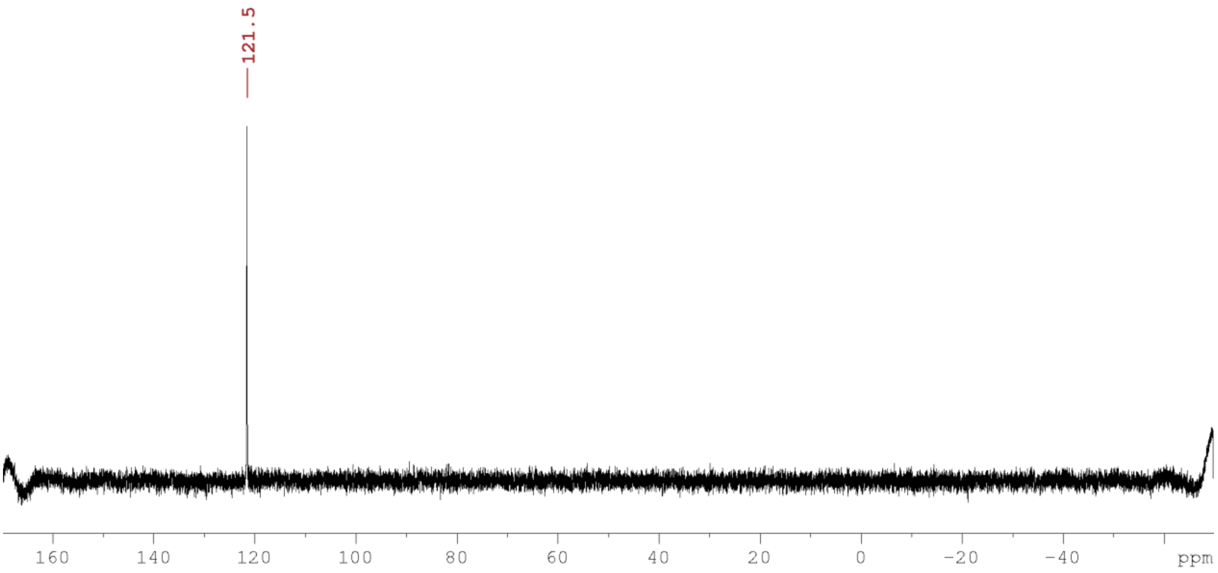

Figure S28. $^{29}Si\{^{1}H\}$ NMR spectrum (99.4 Hz) of [Mn(CO)$_3$(Dipp$_2$NHSi)$_2$(Br)] in d^8-toluene at -40 °C.

NMR Spectra of [(η^5-C_5H_5)Fe(CO)$_2$(Dipp$_2$NHSi-I)] (10)

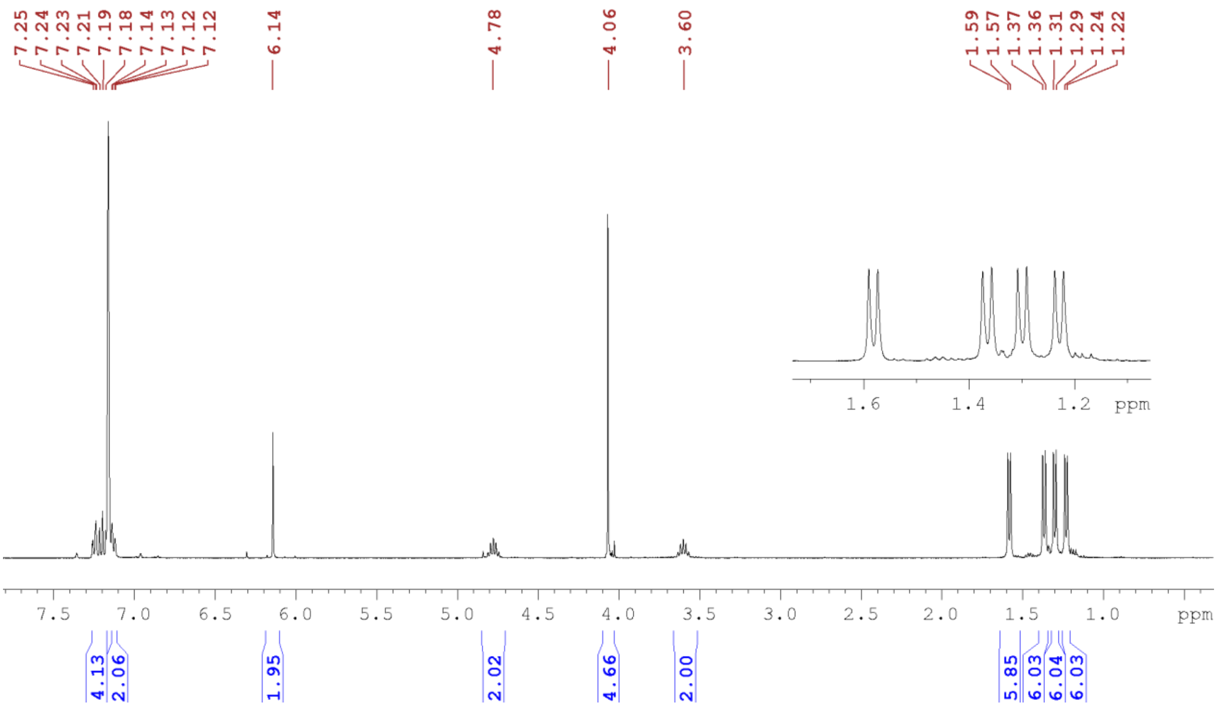

Figure S29. ^{1}H NMR spectrum (400.3 Hz) of [(η^5-C_5H_5)Fe(CO)$_2$(Dipp$_2$NHSi-I)] in C_6D_6.

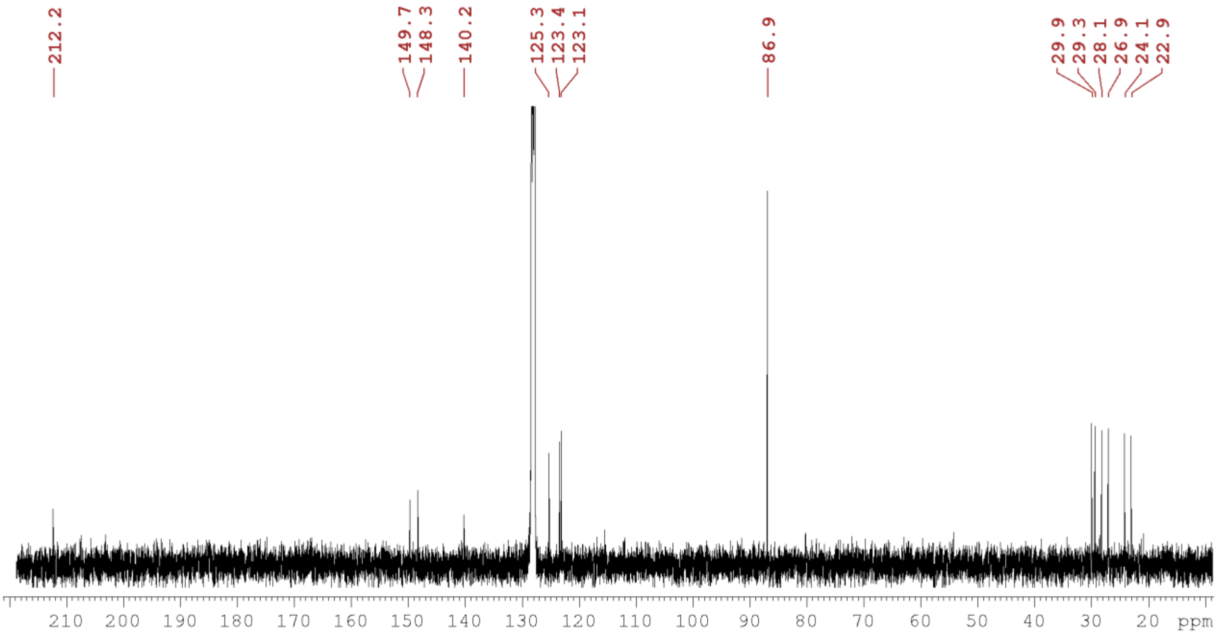

Figure S30. ^{13}C{^{1}H} NMR spectrum (100.7 Hz) of [(η^5-C_5H_5)Fe(CO)$_2$(Dipp$_2$NHSi-I)] in C_6D_6.

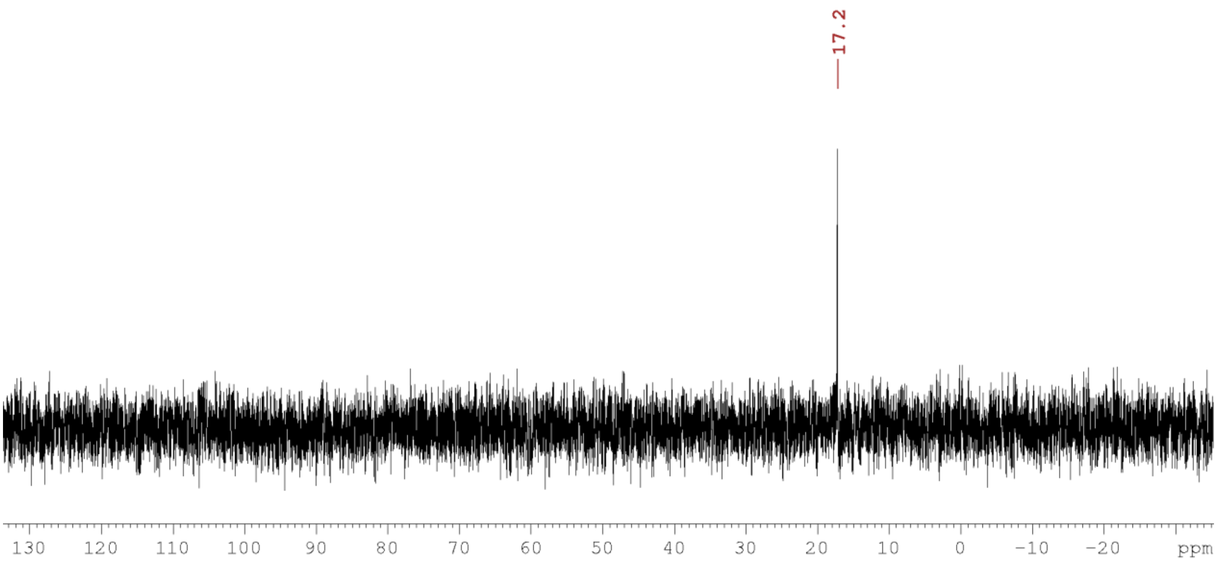

Figure S31. $^{29}Si\{^1H\}$ NMR spectrum (79.5 Hz) of [(η^5-C_5H_5)Fe$(CO)_2$($Dipp_2$NHSi-I)] in C_6D_6.

2. IR Spectra of 2 – 10

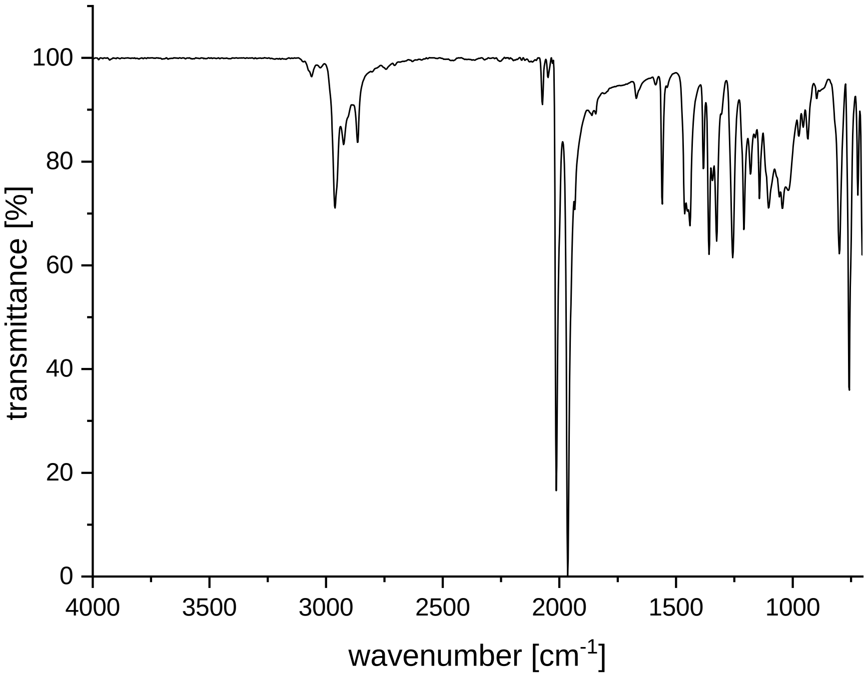

Figure S32. IR spectrum of [{Ni(CO)$_2$(*μ*-Dipp$_2$NHSi)}$_2$] **2**.

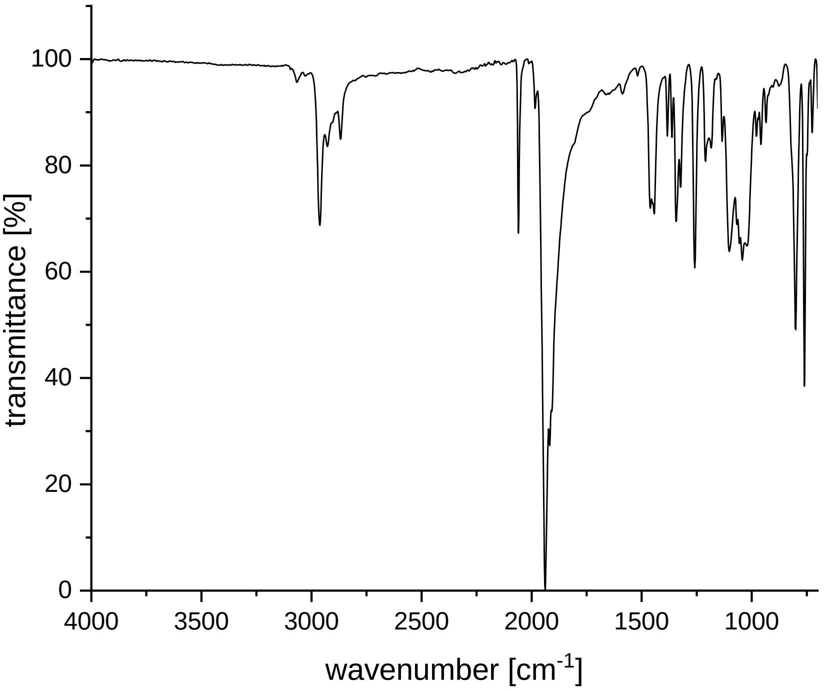

Figure S33. IR spectrum of [Cr(CO)$_5$(Dipp$_2$NHSi)] **3**.

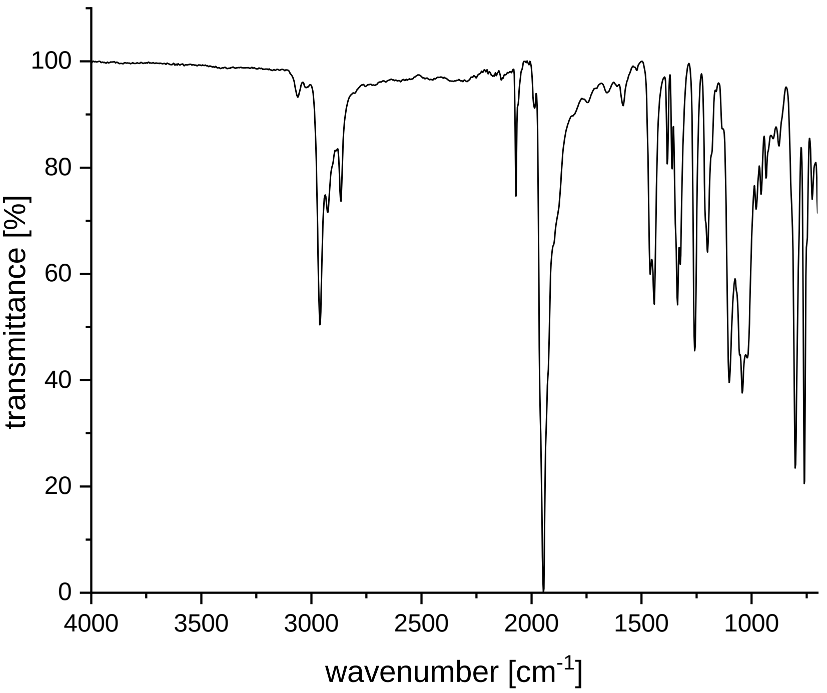

Figure S34. IR spectrum of [Mo(CO)$_5$(Dipp$_2$NHSi)] **4**.

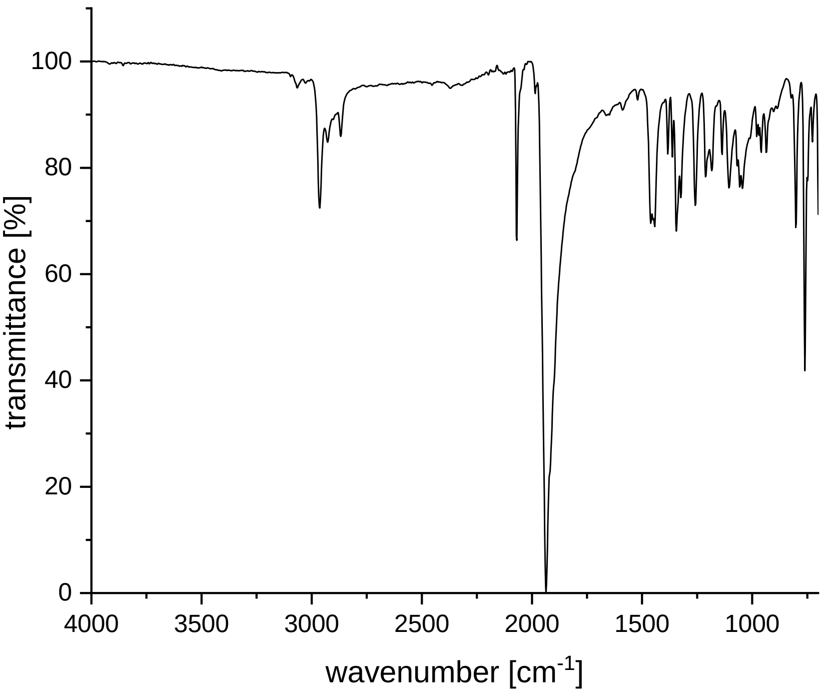

Figure S35. IR spectrum of [W(CO)$_5$(Dipp$_2$NHSi)] **5**.

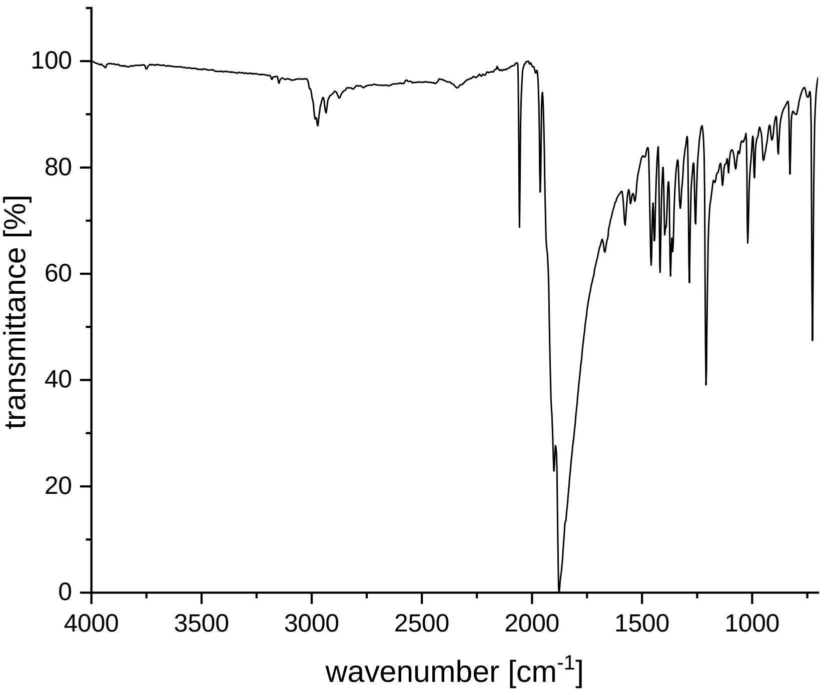

Figure S36. IR spectrum of [W(CO)$_5$(*i*Pr$_2$Im)] **6**.

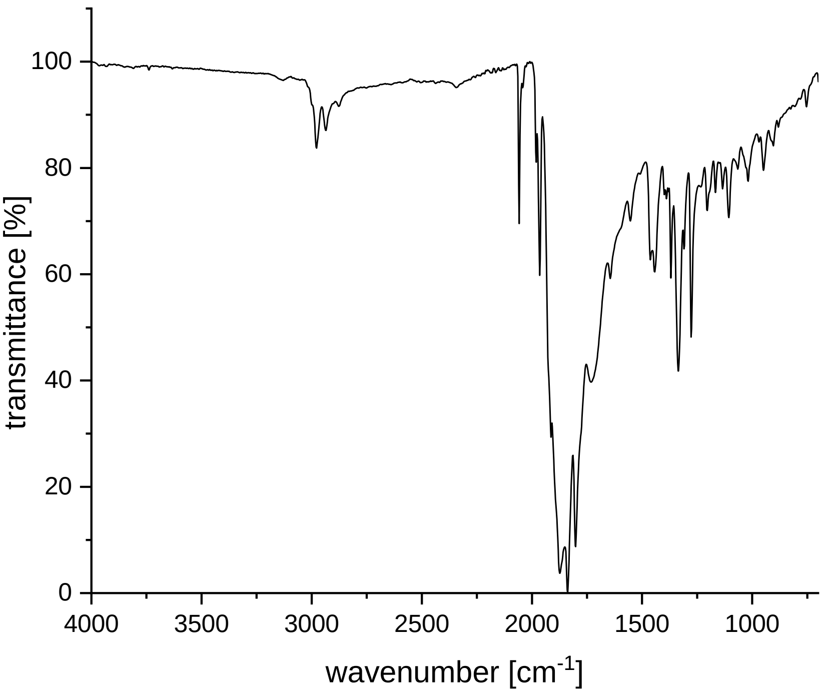

Figure S37. IR spectrum of [W(CO)$_5$(*i*Pr$_2$Im^{Me})] **7**.

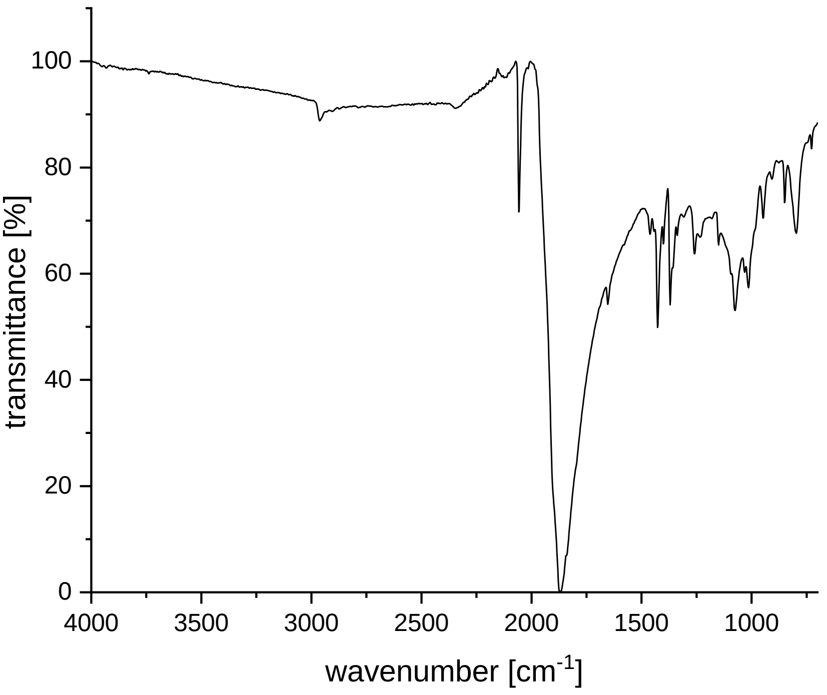

Figure S38. IR spectrum of [W(CO)$_5$(Me$_2$Im^{Me})] **8**.

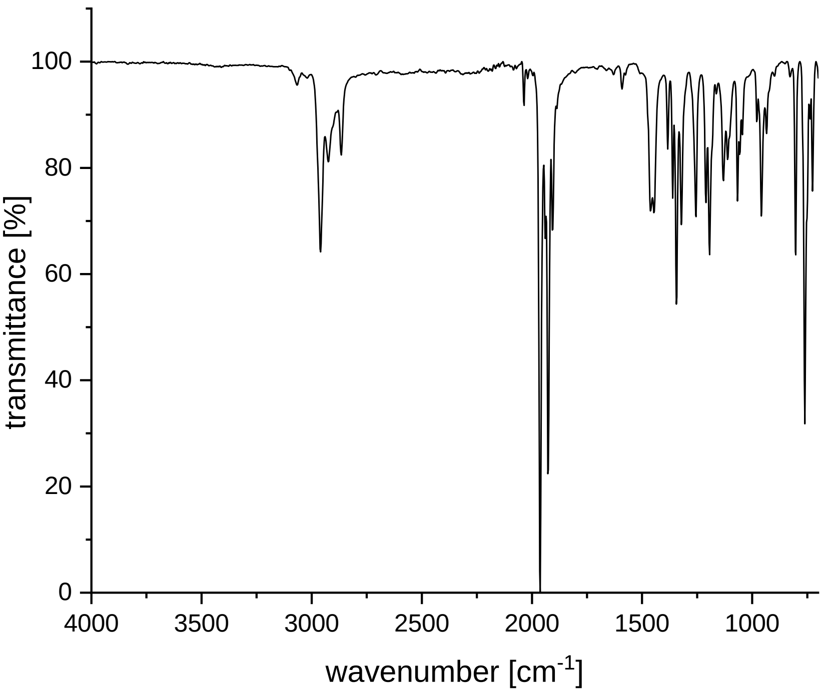

Figure S39. IR spectrum of [Mn(CO)$_3$(Dipp$_2$NHSi)$_2$(Br)] **9**.

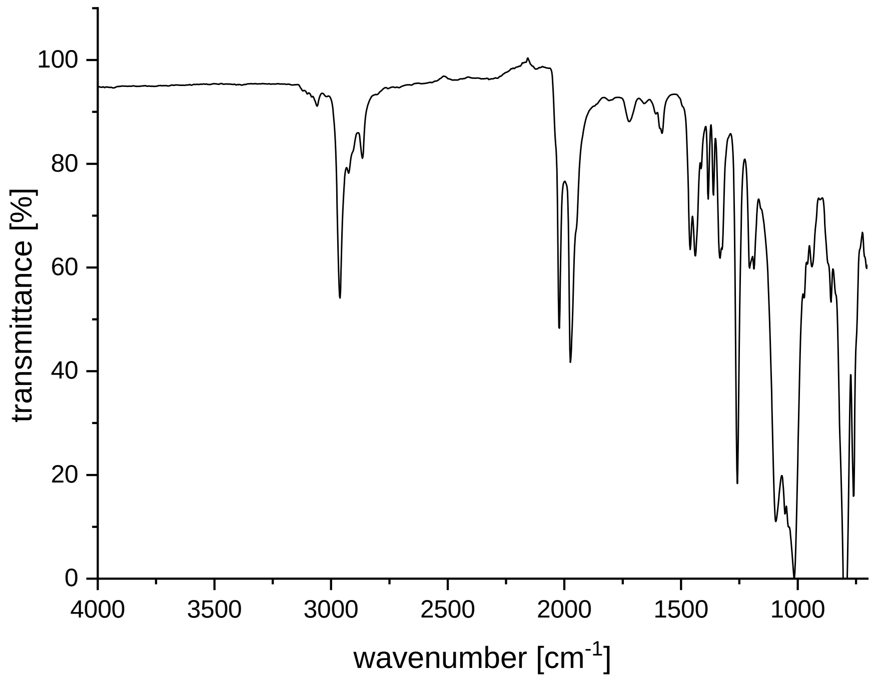

Figure S40. IR spectrum of [(η^5-C_5H_5)Fe(CO)$_2$(Dipp$_2$NHSi-I)] **10**.

3. Computational details

Coordinates of the optimized structures:

Me_2ImNHC^{Me} (def2-TZVPP/B3LYP-D3BJ)

Energy = -304.7470927177

C	0.0000000	0.0000000	-0.9722557
N	0.0000000	-1.0600902	-0.1160694
C	0.0000000	-0.6759629	1.2163015
N	0.0000000	1.0600902	-0.1160694
C	0.0000000	0.6759629	1.2163015
H	0.0000000	1.3766999	2.0322110
H	0.0000000	-1.3766999	2.0322110
C	0.0000000	2.4382448	-0.5638534
H	-0.8878175	2.9617090	-0.2057543
H	0.8878175	2.9617090	-0.2057543
H	0.0000000	2.4314548	-1.6494794
C	0.0000000	-2.4382448	-0.5638534
H	0.8878175	-2.9617090	-0.2057543
H	-0.8878175	-2.9617090	-0.2057543
H	0.0000000	-2.4314548	-1.6494794

Me_2NHSi (def2-TZVPP/B3LYP-D3BJ)

Energy = -556.1920957251

Si	0.0000000	0.0000000	1.5394837
N	0.0000000	1.2131158	0.2561336
N	0.0000000	-1.2131158	0.2561336
C	0.0000000	0.6765768	-1.0184298
H	0.0000000	1.3125563	-1.8904303
C	0.0000000	-0.6765768	-1.0184298
H	0.0000000	-1.3125563	-1.8904303
C	0.0000000	-2.6550794	0.4291641
H	-0.8862389	-3.1082993	-0.0217673
H	0.8862389	-3.1082993	-0.0217673

H	0.0000000	-2.8931674	1.4919997
C	0.0000000	2.6550794	0.4291641
H	0.8862389	3.1082993	-0.0217673
H	-0.8862389	3.1082993	-0.0217673
H	0.0000000	2.8931674	1.4919997

Dipp$_2$NHSi (def2-TZVPP/B3LYP-D3BJ)

Energy = -1411.289935965

Si	0.0000000	0.0000000	-1.1114541
N	0.0000000	1.2093465	0.1803776
N	0.0000000	-1.2093465	0.1803776
C	0.0000000	0.6755754	1.4615432
H	0.0000000	1.3217287	2.3244279
C	0.0000000	-0.6755754	1.4615432
H	0.0000000	-1.3217287	2.3244279
C	0.0000000	-2.6277565	-0.0053500
C	1.2261554	-3.3036389	-0.1064892
C	-1.2261554	-3.3036389	-0.1064892
C	1.2011820	-4.6777374	-0.3385783
C	-1.2011820	-4.6777374	-0.3385783
C	0.0000000	-5.3601923	-0.4576751
H	2.1319340	-5.2206514	-0.4302765
H	-2.1319340	-5.2206514	-0.4302765
H	0.0000000	-6.4265915	-0.6413301
C	0.0000000	2.6277565	-0.0053500
C	1.2261554	3.3036389	-0.1064892
C	-1.2261554	3.3036389	-0.1064892
C	1.2011820	4.6777374	-0.3385783
C	-1.2011820	4.6777374	-0.3385783
C	0.0000000	5.3601923	-0.4576751
H	2.1319340	5.2206514	-0.4302765
H	-2.1319340	5.2206514	-0.4302765
H	0.0000000	6.4265915	-0.6413301

C	2.5475520	-2.5627789	-0.0086360
H	2.3429613	-1.5801295	0.4138366
C	-2.5475520	-2.5627789	-0.0086360
H	-2.3429613	-1.5801295	0.4138366
C	3.5464868	-3.2582824	0.9219319
H	3.1170466	-3.4210506	1.9110546
H	3.8618067	-4.2263057	0.5299587
H	4.4417344	-2.6449403	1.0358340
C	3.1504034	-2.3467740	-1.4037820
H	3.3727837	-3.3023933	-1.8826276
H	2.4608456	-1.8013063	-2.0487019
H	4.0784512	-1.7757109	-1.3377826
C	-3.1504034	-2.3467740	-1.4037820
H	-4.0784512	-1.7757109	-1.3377826
H	-2.4608456	-1.8013063	-2.0487019
H	-3.3727837	-3.3023933	-1.8826276
C	-3.5464868	-3.2582824	0.9219319
H	-3.8618067	-4.2263057	0.5299587
H	-3.1170466	-3.4210506	1.9110546
H	-4.4417344	-2.6449403	1.0358340
C	-2.5475520	2.5627789	-0.0086360
H	-2.3429613	1.5801295	0.4138366
C	-3.5464868	3.2582824	0.9219319
H	-3.1170466	3.4210506	1.9110546
H	-3.8618067	4.2263057	0.5299587
H	-4.4417344	2.6449403	1.0358340
C	-3.1504034	2.3467740	-1.4037820
H	-3.3727837	3.3023933	-1.8826276
H	-2.4608456	1.8013063	-2.0487019
H	-4.0784512	1.7757109	-1.3377826
C	2.5475520	2.5627789	-0.0086360
H	2.3429613	1.5801295	0.4138366
C	3.1504034	2.3467740	-1.4037820

H	2.4608456	1.8013063	-2.0487019
H	3.3727837	3.3023933	-1.8826276
H	4.0784512	1.7757109	-1.3377826
C	3.5464868	3.2582824	0.9219319
H	3.8618067	4.2263057	0.5299587
H	3.1170466	3.4210506	1.9110546
H	4.4417344	2.6449403	1.0358340

cAAC^{Me} (def2-TZVPP/B3LYP-D3BJ)

Energy = -835.9285730592

C	1.8171789	0.7928584	-0.4277512
C	1.9360574	1.4782747	0.9250993
C	1.9061722	3.0103927	0.6400046
C	2.2208570	3.1803210	-0.8547027
H	0.9020120	3.4043573	0.8510758
H	2.6150082	3.5679349	1.2659055
C	0.7776386	1.0441377	1.8324298
H	-0.1912245	1.3034168	1.3834064
H	0.7969678	-0.0422395	1.9867719
H	0.8461287	1.5409897	2.8118722
C	3.2724857	1.0237650	1.5452692
H	3.2979487	-0.0693189	1.6385893
H	4.1307244	1.3266214	0.9304189
H	3.3947393	1.4662411	2.5447418
C	1.2991314	4.1743602	-1.5601543
H	1.5278940	5.1897716	-1.2090945
H	1.4462001	4.1533542	-2.6481217
H	0.2431822	3.9759086	-1.3454189
C	3.6747346	3.5801534	-1.1257329
H	3.9075430	3.5202273	-2.1970053
H	3.8297899	4.6195781	-0.8062489
H	4.3838958	2.9496995	-0.5775714
N	1.9692870	1.7282176	-1.3313718

C	1.9437097	1.4345276	-2.7454307
C	0.7135371	1.5105956	-3.4293349
C	3.1325236	1.0473481	-3.3948705
C	0.7179503	1.3004881	-4.8135612
C	3.0861183	0.8487783	-4.7811051
C	1.8976037	0.9995455	-5.4907691
H	-0.2193867	1.3569764	-5.3662348
H	3.9924894	0.5522873	-5.3083059
H	1.8839173	0.8495379	-6.5705080
C	-0.6010510	1.6568421	-2.6818313
H	-0.3846647	2.0875494	-1.6972376
C	-1.6103954	2.5711732	-3.3840797
H	-2.5028475	2.7010321	-2.7566950
H	-1.1841402	3.5634495	-3.5845872
H	-1.9465837	2.1497534	-4.3414493
C	-1.1834981	0.2544271	-2.4248669
H	-2.1206552	0.3245906	-1.8542171
H	-1.3961093	-0.2580561	-3.3740066
H	-0.4692006	-0.3528286	-1.8529580
C	4.3892296	0.6940656	-2.6181580
H	4.3156775	1.1542473	-1.6273927
C	5.6799909	1.1956889	-3.2746120
H	5.6486376	2.2791549	-3.4531338
H	6.5411306	0.9805222	-2.6272633
H	5.8675125	0.7022164	-4.2382968
C	4.4209853	-0.8287469	-2.3910610
H	5.3041896	-1.1112814	-1.8003746
H	3.5208243	-1.1473034	-1.8490662
H	4.4622398	-1.3654288	-3.3497590

Dipp$_2$Im

Energy = -1159.838897988

C	0.0000000	0.0000000	0.2569872

N	-0.5081914	0.9291228	-0.6044221
C	-0.3253276	0.5912333	-1.9409526
H	-0.6682402	1.2131249	-2.7474288
C	0.3253276	-0.5912333	-1.9409526
H	0.6682402	-1.2131249	-2.7474288
N	0.5081914	-0.9291228	-0.6044221
C	1.1684473	-2.1292499	-0.1843345
C	0.4155085	-3.3065266	-0.0758450
C	2.5374293	-2.0831607	0.0969163
C	1.0782975	-4.4639834	0.3253716
C	3.1568189	-3.2667109	0.4984436
C	2.4369072	-4.4452702	0.6090593
H	0.5287410	-5.3891922	0.4233828
H	4.2131056	-3.2633523	0.7301489
H	2.9343478	-5.3543105	0.9209865
C	-1.1684473	2.1292499	-0.1843345
C	-0.4155085	3.3065266	-0.0758450
C	-2.5374293	2.0831607	0.0969163
C	-1.0782975	4.4639834	0.3253716
C	-3.1568189	3.2667109	0.4984436
C	-2.4369072	4.4452702	0.6090593
H	-0.5287410	5.3891922	0.4233828
H	-4.2131056	3.2633523	0.7301489
H	-2.9343478	5.3543105	0.9209865
C	3.3280130	-0.7909093	0.0227116
H	2.6976064	-0.0396609	-0.4499096
C	-1.0838434	-3.2991822	-0.3113559
H	-1.3085154	-2.4764168	-0.9902238
C	4.5944403	-0.9296141	-0.8289128
H	5.3046749	-1.6299277	-0.3869091
H	4.3582355	-1.2825492	-1.8336106
H	5.0962523	0.0355072	-0.9160746
C	3.6542634	-0.2881706	1.4353728

H	2.7405039	-0.1434505	2.0104026
H	4.2841032	-1.0038974	1.9672160
H	4.1869056	0.6631207	1.3901159
C	-1.8147296	-3.0133850	1.0093485
H	-1.4769497	-2.0725022	1.4415137
H	-2.8922836	-2.9530620	0.8455315
H	-1.6243454	-3.8107840	1.7305471
C	-1.6053550	-4.5810715	-0.9647044
H	-1.0653344	-4.8104604	-1.8842999
H	-1.5189504	-5.4414828	-0.2995925
H	-2.6623721	-4.4681967	-1.2098274
C	-3.3280130	0.7909093	0.0227116
H	-2.6976064	0.0396609	-0.4499096
C	1.0838434	3.2991822	-0.3113559
H	1.3085154	2.4764168	-0.9902238
C	-4.5944403	0.9296141	-0.8289128
H	-5.3046749	1.6299277	-0.3869091
H	-4.3582355	1.2825492	-1.8336106
H	-5.0962523	-0.0355072	-0.9160746
C	-3.6542634	0.2881706	1.4353728
H	-2.7405039	0.1434505	2.0104026
H	-4.2841032	1.0038974	1.9672160
H	-4.1869056	-0.6631207	1.3901159
C	1.6053550	4.5810715	-0.9647044
H	1.0653344	4.8104604	-1.8842999
H	1.5189504	5.4414828	-0.2995925
H	2.6623721	4.4681967	-1.2098274
C	1.8147296	3.0133850	1.0093485
H	1.6243454	3.8107840	1.7305471
H	1.4769497	2.0725022	1.4415137
H	2.8922836	2.9530620	0.8455315

Dipp$_2$Im^{H2}

Energy = -1161.038663952

C	0.0000000	0.0000000	0.2550945
N	-0.4603086	0.9677252	-0.5536278
C	-0.4250121	0.6340772	-1.9969925
H	0.0103156	1.4472878	-2.5765801
C	0.4250121	-0.6340772	-1.9969925
H	-0.0103156	-1.4472878	-2.5765801
N	0.4603086	-0.9677252	-0.5536278
C	1.1284697	-2.1480979	-0.1140372
C	0.3983458	-3.3452728	-0.0420382
C	2.4905755	-2.0933990	0.2062940
C	1.0655504	-4.4991532	0.3597884
C	3.1181687	-3.2725526	0.6088176
C	2.4158789	-4.4642589	0.6812801
H	0.5284131	-5.4341651	0.4303564
H	4.1672289	-3.2557270	0.8720199
H	2.9191287	-5.3699857	0.9936819
C	-1.1284697	2.1480979	-0.1140372
C	-0.3983458	3.3452728	-0.0420382
C	-2.4905755	2.0933990	0.2062940
C	-1.0655504	4.4991532	0.3597884
C	-3.1181687	3.2725526	0.6088176
C	-2.4158789	4.4642589	0.6812801
H	-0.5284131	5.4341651	0.4303564
H	-4.1672289	3.2557270	0.8720199
H	-2.9191287	5.3699857	0.9936819
C	3.2671402	-0.7910765	0.1726084
H	2.6307707	-0.0342633	-0.2830031
C	-1.0944672	-3.3501479	-0.3144737
H	-1.3011096	-2.5496389	-1.0258406
C	4.5429269	-0.8938945	-0.6705024
H	5.2567570	-1.5961533	-0.2375109

H	4.3212136	-1.2286841	-1.6849043
H	5.0346328	0.0785574	-0.7315950
C	3.5754583	-0.3170703	1.5989982
H	2.6545495	-0.1945213	2.1678411
H	4.2067842	-1.0386232	2.1209355
H	4.0990078	0.6401641	1.5809882
C	-1.8575178	-3.0117129	0.9751266
H	-1.5242022	-2.0585810	1.3831346
H	-2.9306776	-2.9519223	0.7830903
H	-1.6895109	-3.7831669	1.7292283
C	-1.6055085	-4.6534502	-0.9313231
H	-1.0437229	-4.9207779	-1.8274243
H	-1.5392468	-5.4875486	-0.2313005
H	-2.6558911	-4.5473487	-1.2065187
C	-3.2671402	0.7910765	0.1726084
H	-2.6307707	0.0342633	-0.2830031
C	1.0944672	3.3501479	-0.3144737
H	1.3011096	2.5496389	-1.0258406
C	-4.5429269	0.8938945	-0.6705024
H	-5.2567570	1.5961533	-0.2375109
H	-4.3212136	1.2286841	-1.6849043
H	-5.0346328	-0.0785574	-0.7315950
C	-3.5754583	0.3170703	1.5989982
H	-2.6545495	0.1945213	2.1678411
H	-4.2067842	1.0386232	2.1209355
H	-4.0990078	-0.6401641	1.5809882
C	1.6055085	4.6534502	-0.9313231
H	1.0437229	4.9207779	-1.8274243
H	1.5392468	5.4875486	-0.2313005
H	2.6558911	4.5473487	-1.2065187
C	1.8575178	3.0117129	0.9751266
H	1.6895109	3.7831669	1.7292283
H	1.5242022	2.0585810	1.3831346

H 2.9306776 2.9519223 0.7830903

H -1.4402455 0.4612617 -2.3640643

H 1.4402455 -0.4612617 -2.3640643

Coordinates of the optimized structures (ADF/TZ2P/BLYP/ZORA/D3(BJ) in [kcal/mol])

NHC^{Me}	C_{2v}	NOIMAG	[-1974.5]	NHSi^{Me}	C_{2v}	NOIMAG	[-[illegible]24.76]	PPh_3	C_{3v}	NOIMA	[-[illegible]96.8]
C	0.000000	0.000000	-0.983311	Si	0.000000	0.000000	1.560835	P	0.00000	0.00000	2.02005
N	0.000000	-1.067638	-0.115510	N	0.000000	1.222355	0.257167	C	1.70368	0.00000	2.76796
C	0.000000	-0.681284	1.227759	N	0.000000	-1.222355	0.257167	C	2.77741	0.00000	1.86007
N	0.000000	1.067638	-0.115510	C	0.000000	0.682421	-024756	C	1.99153	0.00000	4.14364
C	0.000000	0.681284	1.227759	H	0.000000	1.320544	-902222	C	4.10056	0.00000	2.31038
H	0.000000	1.384714	2.048595	C	0.000000	-0.682421	-024756	H	2.55187	0.00000	0.79433
H	0.000000	-1.384714	2.048595	H	0.000000	-1.320544	-902222	C	3.31451	0.00000	4.59600
C	0.000000	2.457724	-0.566676	C	0.000000	-2.677522	0.430736	H	1.19606	0.00000	4.87874
H	-0.893186	2.981040	-0.204829	H	-0.891632	-3.129953	-023747	C	4.37340	0.00000	3.68200
H	0.893186	2.981040	-0.204829	H	0.891632	-3.129953	-023747	H	4.91604	0.00000	1.59009
H	0.000000	2.447448	-1.657759	H	0.000000	-2.914300	1.499386	H	3.51662	0.00000	5.66518
C	0.000000	-2.457724	-0.566676	C	0.000000	2.677522	0.430736	H	5.40165	0.00000	4.03711
H	0.893186	-2.981040	-0.204829	H	0.891632	3.129953	-023747	C	-851840	1.47543	2.76796
H	-0.893186	-2.981040	-0.204829	H	-0.891632	3.129953	-023747	C	-995766	1.72471	4.14364
H	0.000000	-2.447448	-1.657759	H	0.000000	2.914300	1.499386	C	-388705	2.40530	1.86007
								C	-657257	2.87045	4.59600
								H	-598035	1.03582	4.87874
								C	-050283	3.55119	2.31038
								H	-275940	2.20999	0.79433
								C	-186704	3.78748	3.68200
								H	-758314	3.04548	5.66518
								H	-458020	4.25741	1.59009
								H	-700827	4.67796	4.03711
								C	-851840	-475430	2.76796

C	-995766	-724717	4.14364
C	-388705	-405308	1.86007
C	-657257	-870453	4.59600
H	-598035	-035826	4.87874
C	-050283	-551195	2.31038
H	-275940	-209992	0.79433
C	-186704	-787482	3.68200
H	-758314	-045489	5.66518
H	-458020	-257416	1.59009
H	-700827	-677969	4.03711

**[Ni(NHC^{Me})]**	**C_{2v}**	**NOIMAG**	**[-2049.0]**	**[Ni(NHSi^{Me})]**	**C_{2v}**	**NOIMAG**	**[-1987.0]**	**[Ni(PPh$_3$)**	**C_{3v}**	**NOIMA**	**[-[illegible]60.6]**
Ni	0.000000	0.000000	0.000000	Ni	0.000000	0.000000	0.000000	Ni	0.00000	0.00000	0.00000
C	0.000000	0.000000	1.752969	Si	0.000000	0.000000	2.002338	P	0.00000	0.00000	2.02005
N	1.090108	0.000000	2.619785	N	1.232193	0.000000	3.267885	C	1.70368	0.00000	2.76796
C	0.681490	0.000000	3.950161	C	0.681779	0.000000	4.553893	C	2.77741	0.00000	1.86007
N	-1.090108	0.000000	2.619785	N	-1.232193	0.000000	3.267885	C	1.99153	0.00000	4.14364
C	-0.681490	0.000000	3.950161	C	-0.681779	0.000000	4.553893	C	4.10056	0.00000	2.31038
H	-1.382415	0.000000	4.771970	H	-1.321773	0.000000	5.428804	H	2.55187	0.00000	0.79433
H	1.382415	0.000000	4.771970	H	1.321773	0.000000	5.428804	C	3.31451	0.00000	4.59600
C	-2.479934	0.000000	2.173362	C	-2.682986	0.000000	3.079783	H	1.19606	0.00000	4.87874
H	-2.999746	-0.895326	2.535184	H	-3.140582	-0.892518	3.528240	C	4.37340	0.00000	3.68200
H	-2.999746	0.895326	2.535184	H	-3.140582	0.892518	3.528240	H	4.91604	0.00000	1.59009
H	-2.466024	0.000000	1.079267	H	-2.899969	0.000000	2.007033	H	3.51662	0.00000	5.66518
C	2.479934	0.000000	2.173362	C	2.682986	0.000000	3.079783	H	5.40165	0.00000	4.03711
H	2.999746	0.895326	2.535184	H	3.140582	0.892518	3.528240	C	-851840	1.47543	2.76796
H	2.999746	-0.895326	2.535184	H	3.140582	-0.892518	3.528240	C	-995766	1.72471	4.14364
H	2.466024	0.000000	1.079267	H	2.899969	0.000000	2.007033	C	-388705	2.40530	1.86007
								C	-657257	2.87045	4.59600
								H	-598035	1.03582	4.87874
								C	-050283	3.55119	2.31038

H	- 275940	2.20999	0.79433
C	- 186704	3.78748	3.68200
H	- 758314	3.04548	5.66518
H	- 458021	4.25741	1.59009
H	- 700827	4.67796	4.03711
C	- 851840	- 475430	2.76796
C	- 995766	- 724717	4.14364
C	- 388705	- 405308	1.86007
C	- 657257	- 870453	4.59600
H	- 598035	- 035826	4.87874
C	- 050283	- 551195	2.31038
H	- 275940	- 209992	0.79433
C	- 186704	- 787482	3.68200
H	- 758314	- 045489	5.66518
H	- 458021	- 257416	1.59009
H	- 700827	- 677969	4.03711

$[Ni(NHC^{Me})(CO)_3]$	**Cs**	**NOIMAG**	**[-3172.19]**	$[Ni(NHSi^{Me})(CO)_3]$	**Cs**	**NOIMAG**	**[-3114.6]**	$[Ni(PPh_3)(CO)_3$	C_{3v}	**NOIMA**	**[-)79.0]**
Ni	0.000000	0.000000	0.000000	Ni	0.000000	0.000000	0.000000	Ni	0.00000	0.00000	0.00000
C	-0.585630	-0.861910	1.473230	C	-0.472660	-0.887850	1.500640	P	0.00000	0.00000	2.20000
C	-0.489690	1.737410	-0.000090	C	-0.501090	1.741910	0.000000	C	1.70368	0.00000	2.94790
C	-0.585630	-0.862070	-1.473140	C	-0.472660	-0.887850	- 500640	C	2.77741	0.00000	2.04001
O	-0.993310	-1.419400	-2.404540	O	-0.748700	-1.455500	2.468710	C	1.99153	0.00000	4.32358
O	-0.790970	2.856070	-0.000150	O	-0.800340	2.857040	0.000000	C	4.10056	0.00000	2.49032
O	-0.993310	-1.419140	2.404700	O	-0.748700	-1.455500	- 468710	H	2.55187	0.00000	0.97427
C	1.997110	0.000000	0.000000	Si	2.219320	0.000000	0.000000	C	3.31451	0.00000	4.77595
H	5.001640	-0.001480	1.387080	N	3.474380	-0.024170	1.230200	H	1.19606	0.00000	5.05868
N	2.849080	0.003400	1.077440	N	3.474380	-0.024170	- 230200	C	4.37340	0.00000	3.86194
N	2.849080	0.003290	-1.077440	C	4.763990	-0.047670	0.684260	H	4.91604	0.00000	1.77004
C	4.185380	-0.006150	-0.680550	H	5.635430	-0.061640	1.328180	H	3.51662	0.00000	5.84512
C	2.411130	0.005600	-2.475700	C	4.763990	-0.047670	- 684260	H	5.40165	0.00000	4.21705

H	1.318743	0.004266	-2.501636	H	5.635430	-0.061640	-328180	C	-851840	1.47543	2.94790
H	2.788760	0.891175	-2.995966	C	3.297920	-0.020100	-684520	C	-995766	1.72471	4.32358
H	2.792595	-0.887077	-2.974382	H	3.768850	0.863351	-133971	C	-388706	2.40530	2.04001
C	2.411130	0.005870	2.475700	H	3.733244	-0.921139	-134829	C	-657256	2.87045	4.77595
H	2.788767	0.891517	2.995862	H	2.229406	0.001005	-915830	H	-598034	1.03582	5.05868
H	1.318743	0.004551	2.501641	C	3.297920	-0.020100	2.684520	C	-050283	3.55119	2.49032
H	2.792596	-0.886747	2.974485	H	3.733244	-0.921139	3.134829	H	-275940	2.20999	0.97427
H	5.001640	-0.001620	-1.387080	H	3.768850	0.863351	3.133971	C	-186704	3.78748	3.86194
C	4.185380	-0.006080	0.680550	H	2.229406	0.001005	2.915830	H	-758314	3.04548	5.84512
								H	-458021	4.25741	1.77004
								H	-700827	4.67797	4.21705
								C	-851840	-475430	2.94790
								C	-995766	-724717	4.32358
								C	-388706	-405309	2.04001
								C	-657256	-870452	4.77595
								H	-598034	-035826	5.05868
								C	-050283	-551194	2.49032
								H	-275940	-209992	0.97427
								C	-186704	-787482	3.86194
								H	-758314	-045489	5.84512
								H	-458021	-257416	1.77004
								H	-700827	-677970	4.21705
								C	1.81019	0.00000	-639999
								C	-905097	-567673	-639999
								C	-905096	1.56767	-640000
								O	2.86180	0.00000	-011799
								O	-430902	-478394	-011799
								O	-430901	2.47839	-011801

**[W(NHC^{Me})(CO)$_5$]**	**C_{2v}**	**NOIMAG**	**[-4007.1]**	**[W(NHSi^{Me})(CO)$_5$]**	**C_{2v}**	**NOIMAG**	**[-3951.6]**
W	0.000000	0.000000	0.000000	W	0.000000	0.000000	0.000000
C	0.000000	0.000000	2.050465	C	0.000000	0.000000	2.058630

C	2.059266	0.000000	-0.027902	C	2.064946	0.000000	-000688
C	-2.033068	-0.327433	-0.027902	C	-2.063178	-0.085427	-000688
C	-0.159325	1.991275	0.339374	C	-0.042110	2.034924	0.032760
C	-0.054007	0.674996	-1.935425	C	-0.001371	0.066237	-057563
O	-0.015496	0.193667	3.198984	O	-0.000552	0.026690	3.218276
O	3.217484	-0.057397	-0.053316	O	3.222854	-0.007598	-001195
O	-3.167425	-0.568262	-0.053316	O	-3.219781	-0.140921	-001195
O	-0.250757	3.134021	0.534133	O	-0.066098	3.194100	0.051421
O	-0.098885	1.235882	-2.955345	O	-0.002695	0.130226	-215750
C	0.182183	-2.276962	-0.388064	Si	0.052184	-2.521727	-040597
N	0.235769	-2.946693	-1.595952	N	0.077662	-3.752929	-291911
N	0.264577	-3.306745	0.530174	N	0.078482	-3.792552	1.170438
C	0.345898	-4.323110	-1.424533	C	0.104496	-5.049620	-762202
H	0.400389	-5.004151	-2.260443	H	0.122308	-5.910341	-419826
C	0.364012	-4.549509	-0.087633	C	0.104949	-5.071528	0.599264
H	0.437463	-5.467516	0.475750	H	0.123190	-5.952963	1.228841
C	0.256572	-3.206692	1.991826	C	0.075616	-3.654032	2.633361
H	1.186935	-3.619591	2.394847	H	0.977416	-4.104725	3.065813
H	0.173215	-2.164875	2.276833	H	0.053746	-2.597194	2.902884
H	-0.596302	-3.762270	2.394847	H	-0.806768	-4.141647	3.065813
C	0.189714	-2.371089	-2.942451	C	0.073823	-3.567410	-749618
H	-0.675949	-2.766828	-3.483289	H	-0.808853	-4.040867	-196947
H	0.103527	-1.293901	-2.866314	H	0.051785	-2.502447	-984982
H	1.107288	-2.624149	-3.483289	H	0.975331	-4.003946	-196947
